# Supplementary material for: Effects of the COVID-19 pandemic on the mental health of clinically extremely vulnerable children and children living with clinically extremely vulnerable people in Wales: a data linkage study
Source: BMJ Open. 2023 Jun 16;13(6):e067882. doi: 10.1136/bmjopen-2022-067882 (PMC10276955; doi:10.1136/bmjopen-2022-067882)
Supplement: Supplementary data [file bmjopen-2022-067882supp001.pdf]

Appendix

SAIL databank additional information

SAIL databank information governance policies and procedures

The SAIL databank (www.saildatabank.com) is an internationally recognised, remotely-accessible, privacy-protecting data safe haven designed to support observational, interventional, and policy-relevant research to improve population health, well-being, and services.[1–6] SAIL contains anonymised, linkable, routinely collected health, administrative, and social care data for the population of Wales, UK, from multiple sources at individual, household, and ecological levels.[1–6] Data are anonymised using a split-file process, which has been described in detail elsewhere.[1–3] Within each dataset, identifiable and non-identifiable data are separated, and identifiable data are sent to a trusted third party (TTP), Digital Health and Care Wales (DHCW; previously known as the NHS Wales Informatics Service). The TTP uniquely matches identities based on name, NHS number, date of birth, and Unique Property Reference Number (UPRN), using the Matching Algorithm for Consistent Results in Anonymised Linkage, which has an accuracy of 99.85%.[1,2] Individuals and residences are then assigned unique identifiers: for individuals this is called an Anonymised Linking Field (ALF) and for residences a Residential Anonymised Linking Field (RALF). The anonymised and non-identifiable data components are then recombined within SAIL and the linking fields are further encrypted and used to anonymously link between datasets. This enables data from multiple sources, including general practice (GP) data, hospital admissions, outpatient data, and demographic details to be linked at the individual and household level, while preserving anonymity.

References

1. Lyons RA, Jones KH, John G, et al. The SAIL databank: linking multiple health and social care datasets. BMC Med Inform Decis Mak2009;9:3.doi:10.1186/1472-6947-9-3
2. Ford DV, Jones KH, Verplancke JP, et al. The SAIL Databank: building a national architecture for e-health research and evaluation. BMC Health Serv Res2009;9:157.doi:10.1186/1472-6963-9-157
3. Rodgers SE, Lyons RA, Dsilva R, et al. Residential Anonymous Linking Fields (RALFs): a novel information infrastructure to study the interaction between the environment and individuals health. J Public Health2009;31:582–8.doi:10.1093/pubmed/fdp041
4. Rodgers SE, Demmler JC, Dsilva R, Lyons RA. Protecting health data privacy while using residence-based environment and demographic data. *Health Place*. 2012; 18: 209-217.
5. Jones, K. H., Ford, D. V., Thompson, S., & Lyons, R. A. (2019). A profile of the SAIL databank on the UK secure research platform. *International Journal of Population Data Science*, *4*(2). https://doi.org/10.23889/ijpds.v4i2.1134
6. Jones, K. H., Ford, D. V., Jones, C., Dsilva, R., Thompson, S., Brooks, C. J., Heaven, M. L., Thayer, D. S., McNerney, C. L., & Lyons, R. A. (2014). A case study of the secure anonymous information linkage (SAIL) gateway: A privacy-protecting remote access system for health-related research and evaluation. *Journal of Biomedical Informatics*, *50*, 196–204. https://doi.org/10.1016/j.jbi.2014.01.003

Ethical Approval

The Information Governance Review Panel (IGRP) is an independent panel of representatives from various government, regulatory, and professional organisations, who review all proposals for SAIL data access to ensure that they are appropriate with respect to Information Governance, and in the public interest.[1] Data were analysed within the SAIL secure research environment, and appropriate disclosure control procedures were followed to ensure that no personally identifiable data or small numbers (n<5) were removed from the environment. All data within SAIL are treated in accordance with the Data Protection Act 2018 and SAIL complies with the principles of the General Data Protection Regulation (GDPR).

References

1. Ford DV, Jones KH, Verplancke JP, et al. The SAIL Databank: building a national architecture for e-health research and evaluation. BMC Health Serv Res2009;9:157.doi:10.1186/1472-6963-9-157

ICD-10 codes for cancers of the blood or bone marrow

ICD-10 code	Description
C81	Hodgkin lymphoma
C82	Follicular lymphoma
C83	Non-follicular lymphoma
C84	Mature T/NK-cell lymphomas
C85	Other and unspecified types of non-Hodgkin lymphoma
C86	Other specified types of T/NK-cell lymphoma
C88	Malignant immunoproliferative diseases
C90	Multiple myeloma and malignant plasma cell neoplasms
C91	Lymphoid leukaemia
C92	Myeloid leukaemia
C93	Monocytic leukaemia
C94	Other leukaemias of specified cell type
C95	Leukaemia of unspecified cell type
C96	Other and unspecified malignant neoplasms of lymphoid, haematopoietic and related tissue
D470	Histiocytic and mast cell tumours of uncertain and unknown behaviour
D475	Chronic eosinophilic leukaemia [hypereosinophilic syndrome]
D477	Other specified neoplasms of uncertain or unknown behaviour of lymphoid, haematopoietic and related tissue
D479	Neoplasm of uncertain or unknown behaviour of lymphoid, haematopoietic and related tissue, unspecified
D595	Paroxysmal nocturnal haemoglobinuria [Marchiafava-Micheli]
D71X	Functional disorders of polymorphonuclear neutrophils
D730	Hyposplenism
D760	Langerhans' cell histiocytosis, not elsewhere classified
D761	Haemophagocytic lymphohistiocytosis
D898	Other specified disorders involving the immune mechanism, not elsewhere classified
D899	Disorder involving the immune mechanism, unspecified
L412	Lymphomatoid papulosis
P615	Transient neonatal neutropenia

ICD-10 codes for respiratory illnesses

ICD-10 code	Description
E84	Cystic Fibrosis
J84	Other interstitial pulmonary diseases
J620	Pneumoconiosis due to talc dust
J630	Aluminosis (of lung)
J631	Bauxite fibrosis (of lung)
J633	Graphite fibrosis (of lung)
J634	Siderosis
J635	Stannosis

J660	Byssinosis
J661	Flax-dresser disease
J662	Cannabinosis
J668	Airway disease due to other specific organic dusts
J670	Farmer lung
J671	Bagassosis
J678	Hypersensitivity pneumonitis due to other organic dusts
J684	Chronic respiratory conditions due to chemicals, gases, fumes and vapours
J688	Other respiratory conditions due to chemicals, gases, fumes and vapours
J698	Pneumonitis due to other solids and liquids
J701	Chronic and other pulmonary manifestations due to radiation
J703	Chronic drug-induced interstitial lung disorders
J840	Alveolar and parietoalveolar conditions
J983	Compensatory emphysema
J991	Respiratory disorders in other diffuse connective tissue disorders
M313	Wegener granulomatosis
P250	Interstitial emphysema originating in the perinatal period
Q334	Congenital bronchiectasis

OPCS-4 codes for immunosuppression therapy

OPCS-4 code	Description
X353	Active Inflammatory thyroid eye disease patients currently on weekly intravenous steroid infusion treatment (12 weekly injections regime) or immunospressant
X374	Intramuscular Immunotherapy
X385	Subcutaneous Immunotherapy
X891	Monoclonal antibodies Band 1
X892	Monoclonal antibodies Band 2
X893	Patients receiving maintenance treatment with rituximab, obinotuzimab or ofatumumab
X894	Somatostatin analogues Band 1
X895	Allergic emergency drugs Band 1
X961	Patients previously treated for haematological malignancy requiring IV immunoglobulin replacement
X962	Allergen immunotherapy drugs Band 1
X963	Poison management drugs Band 1

Pre-COVID-19 study cohorts

Figure 3 shows a flow diagram of inclusion criteria for the two 2019 study cohorts: children in the general population, and clinically extremely vulnerable children with blood or bone cancer or respiratory illnesses, or receiving immunosuppression therapy.

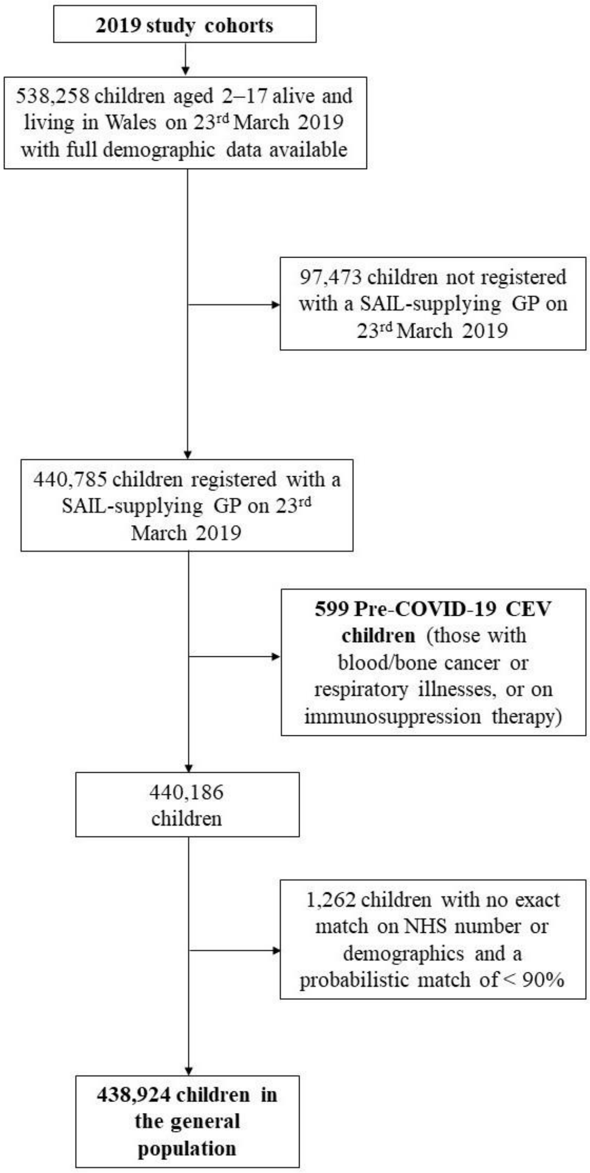

Figure 3. Flow diagram of the inclusion criteria for the creation of the 2019 study cohorts

Cowley LE, *et al. BMJ Open* 2024; 13:e067882. doi: 10.1136/bmjopen-2022-067882

Sensitivity Analysis

We undertook a sensitivity analysis to confirm the validity of creating a cohort of pre-COVID-19 CEV children based on just three of the categories included in the COVID-19 Shielded People List (CVSP). We created a cohort of clinically extremely vulnerable (CEV) children who were added to the CVSP in 2020 for the same three reasons only (respiratory illnesses, blood/bone cancer, and immunosuppression therapy) and a cohort of children in the general population (figure 1). Demographic characteristics of the children in each cohort are presented in Table 1. We plotted the Kaplan-Meier survival curve for each cohort and estimated the hazard ratio of having a record for anxiety or depression during the COVID-19 pandemic (March 23rd 2020–January 31st 2021) using Cox regression (figure 2).

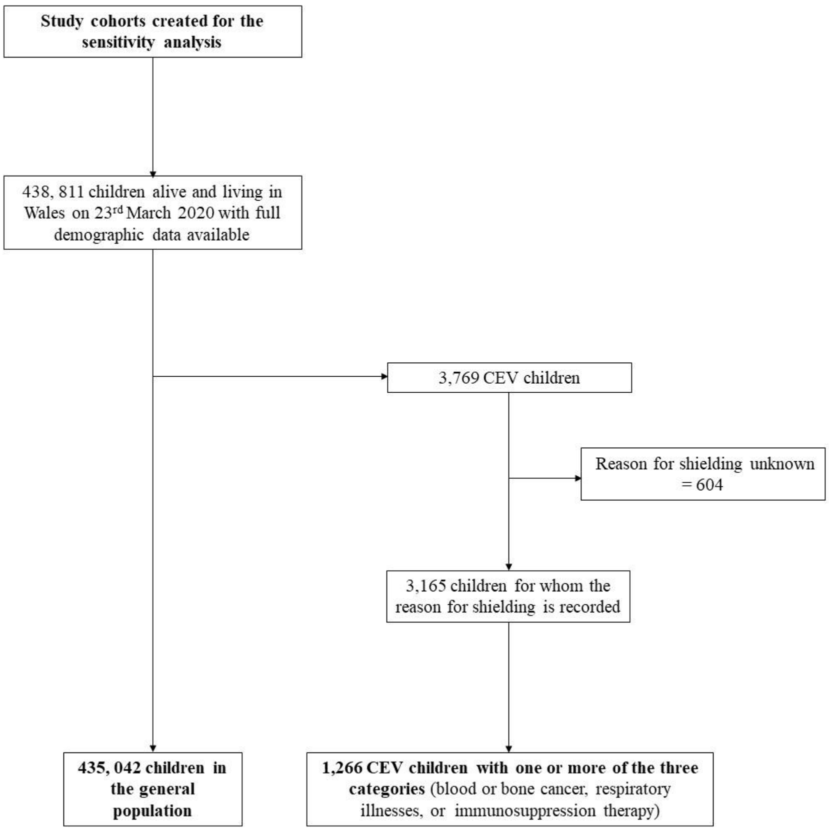

Figure 1. Flow diagram of the inclusion criteria for the creation of the study cohorts for the sensitivity analysis.

		General population 2020	CEV children with one or more of three categories 2020	Chi² P value
N		435,042	1,266	
**Sex (%)**	Male	222,558 (51.2)	712 (56.2)	<0.001
	Female	212,484 (48.8)	554 (43.8)	
**Age group (%)**	2–7	157,691 (36.2)	385 (30.4)	<0.001
	8–12	143,988 (33.1)	396 (31.3)	
	13–17	133,363 (30.7)	485 (38.3)	
**Deprivation quintile (WIMD 2019) (%)**	1 (most deprived)	111,133 (25.5)	329 (26.0)	0.262
	2	92,037 (21.2)	257 (20.3)	
	3	76,717 (17.6)	210 (16.6)	
	4	73,277 (16.8)	203 (16.0)	
	5 (least deprived)	81,878 (18.8)	267 (21.1)	
**Rural/Urban area (%)**	Rural	116,126 (26.7)	336 (26.5)	0.928
	Urban	318,916 (73.3)	930 (73.5)	
**Any history of anxiety or depression**	NO	415,925 (95.6)	1,166 (92.1)	<0.001
	YES	19,117 (4.4)	100 (7.9)	

Table 1. Demographic characteristics of children in the general population and clinically extremely vulnerable children added to the COVID-19 Shielded People List for one or more of three reasons (blood or bone cancer, respiratory illnesses, or immunosuppression therapy)

The results showed a similar pattern to those obtained when analysing the full CEV cohort in that CEV children with one or more of the three categories were at greater risk of having a record for anxiety or depression during the COVID-19 pandemic (March 23rd 2020–January 31st 2021) compared to children in the general population, thereby validating our approach.

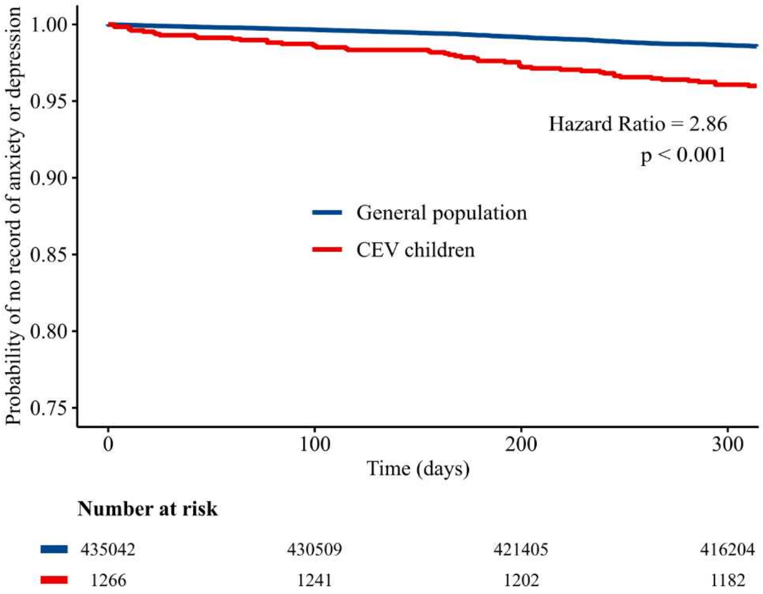

Figure 2. Kaplan-Meier survival curves for each cohort, showing the time to first record of anxiety or depression during the COVID-19 pandemic

CEV, clinically extremely vulnerable

Read v2 and ICD-10 diagnosis codes used to identify records of anxiety or depression in primary or secondary care

Read v2 codes for depression diagnosis

Read v2 code	Description
Eu32.	[X]Depressive episode
Eu320	[X]Mild depressive episode
Eu321	[X]Moderate depressive episode
Eu322	[X]Severe depressive episode without psychotic symptoms
Eu324	[X]Mild depression
Eu32y	[X]Other depressive episodes
Eu32z	[X]Depressive episode, unspecified
Eu33.	[X]Recurrent depressive disorder
Eu330	[X]Recurrent depressive disorder, current episode mild
Eu331	[X]Recurrent depressive disorder, current episode moderate
Eu332	[X]Recurrent depressive disorder, current episode severe without psychotic symptoms
Eu334	[X]Recurrent depressive disorder, currently in remission
Eu33y	[X]Other recurrent depressive disorders
Eu33z	[X]Recurrent depressive disorder, unspecified
Eu341	[X]Dysthymia
E118.	Seasonal affective disorder
E135.	Agitated depression
E2B..	Depressive disorder NEC
E2B1.	Chronic depression
E291.	Prolonged depressive reaction
E204.	Neurotic depression reactive type
E2B0.	Postviral depression
E112.	Single major depressive episode
E1120	Single major depressive episode, unspecified
E1121	Single major depressive episode, mild
E1122	Single major depressive episode, moderate
E1123	Single major depressive episode, severe, without psychosis
E1125	Single major depressive episode, partial or unspecied remission
E1126	Single major depressive episode, in full remission
E112z	Single major depressive episode NOS
E113.	Recurrent major depressive episode
E1130	Recurrent major depressive episodes, unspecified
E1131	Recurrent major depressive episodes, mild
E1132	Recurrent major depressive episodes, moderate
E1133	Recurrent major depressive episodes, severe, no psychosis
E1135	Recurrent major depressive episodes, partial/unspecified remission
E1136	Recurrent major depressive episodes, in full remission
E1137	Recurrent depression
E113z	Recurrent major depressive episode NOS

Read v2 codes for depression symptoms

Read v2 code	Description
1B17.	Depressed
1B1U.	Symptoms of depression
1BQ..	Loss of capacity for enjoyment
1BT..	Depressed mood
1BU..	Loss of hope for the future
2257	O/E – depressed
1BP..	Loss of interest

Read v2 codes for antidepressant prescriptions

Read v2 code	Description
d71..	Amitriptyline hydrochloride
d72..	Butriptyline - discontinued
d73..	Clomipramine hydrochloride
d74..	Desipramine hydrochloride
d75..	Dosulepin Hydrochloride
d76..	Doxepin
d77..	Imipramine hydrochloride
d78..	Iprindole
d79..	Lofepramine
d7a..	Maprotiline hydrochloride
d7b..	Mianserin hydrochloride
d7c..	Nortriptyline
d7d..	Protriptyline hydrochloride
d7e..	Trazadone hydrochloride
d7f..	Trimipramine
d7g..	Viloxazine hydrochloride
d7h..	Amoxapine
d81..	Phenelzine
d83..	Isocarboxazid
d84..	Tranylcypromine
d85..	Moclobemide
d91..	Compound Antidepressants A-Z
da1..	Flupentixol [Antidepressant]
da2..	Tryptophan
da3..	Fluvoxamine Maleate
da4..	Fluoxetine hydrochloride
da5..	Sertraline hydrochloride
da6..	Paroxetine hydrochloride
da7..	Venlafaxine
da9..	Citalopram
daA..	Reboxetine
daB..	Mirtazapine

daC..	Escitalopram
daD..	Agomelatine
gde..	Duloxetine
d911.	Limbitrol 5 capsules - discontinued
d912.	Limbitrol 10 capsules - discontinued
d913.	Motipress tablets
x28CP	Discontinued
d914.	Motival tablets discontinued
d916.	Triptafen tablets - only one not discontinued?
d917.	Triftafen-M tablets - discontinued
d8…	Monoamine-oxidase
d82..	Iproniazid

Read v2 codes for anxiety diagnosis

Read v2 code	Description
Eu41.	[X]Other anxiety disorders
Eu410	[X]Panic disorder [episodic paroxysmal anxiety]
Eu411	[X]Generalized anxiety disorder
Eu413	[X]Other mixed anxiety disorders
Eu41y	[X]Other specified anxiety disorders
Eu41z	[X]Anxiety disorder, unspecified
E200.	Anxiety states
E2000	Anxiety state unspecified
E2001	Panic disorder
E2002	Generalised anxiety disorder
E2004	Chronic anxiety
E2005	Recurrent anxiety
E200z	Anxiety state NOS
E202	Phobic disorders
Eu40.	Phobic anxiety disorder
Eu930	[X]Separation anxiety disorder of childhood *
Eu931	[X]Phobic anxiety disorder of childhood*
Eu932	[X]Social anxiety disorder of childhood*
E2D0.	Disturbance of anxiety and fearfulness in childhood and adolescence*
E2D00	Childhood and adolescent overanxiousness disturbance*
E2D0z	Disturbance of anxiety and fearfulness in childhood and adolescence NOS*

Read v2 codes for anxiety symptoms

Read v2 code	Description
1B13.	Anxiousness
2258	O/E - anxious
1B12.	Nerves, nervousness
R2y2.	(D) nervousness

2259	O/E nervous
225J.	O/E panic attack
1B1V.	C/O panic attack

Read v2 codes for anxiety prescriptions – hypnotics

Read v2 code	Description
d11..	Chloral hydrate
d12..	Clomethiazole edisylate (hypnotic)
d13..	Dichloralphenazone - discontinued
d14..	Flumtrazepam - discontinued
d15..	Flurazepam
d16..	Loprazolam
d17..	Lormetazepam
d18..	Nitrazepam
d1a..	Temazepam (hynotic)
d1b..	Triazolam - discontinued
d1c..	Triclofos sodium
d1d..	Zopiclone
d1f..	Zolpidem
d1g..	Zaleplon
d1h..	Melatonin
d1i..	Dexmedetomidine

Read v2 codes for anxiety prescriptions – hypnotics

Read v2 code	Description
d21..	Diazepam
d22..	Alprazolam
d23..	Bromazepam
d24..	Chlordiazepoxide
d25..	Chlormezanone
d26..	Clobazam
d27..	Clorazepate dipotassium
d28..	Hydroxyzine hcl (anxiolytic)
d29..	Ketazolam - discontinued
d2a..	Lorazepam (anxiolytic)
d2b..	Medazepam - discontinued
d2c..	Meprobamate
d2d..	Oxazepam
d2e..	Prazepam - discontinued
d2f..	Buspirone hydrocholoride
d2g..	Flumazenil

Read v2 codes for mixed anxiety and depression diagnosis

Read v2 code	Description
E2003	Anxiety with depression
Eu412	[X]Mixed anxiety and depressive disorder

ICD-10 diagnosis codes for depression

ICD-10 code	Description
F321	Moderate depressive episode
F322	Severe depressive episode without psychotic symptoms
F328	Other depressive episodes
F329	Depressive episode, unspecified
F33	Recurrent depressive disorder
F330	Recurrent depressive disorder, current episode mild
F331	Recurrent depressive disorder, current episode moderate
F332	Recurrent depressive disorder, current episode severe without psychotic symptoms
F334	Recurrent depressive disorder, currently in remission
F338	Other recurrent depressive disorders
F339	Recurrent depressive disorder, unspecified
F341	Dysthymia

ICD-10 diagnosis codes for anxiety and mixed anxiety and depression

ICD-10 code	Description
F40	Phobic anxiety disorders
F400	Agoraphobia
F401	Social phobias
F402	Specific (isolated) phobias
F408	Other phobic anxiety disorders
F41	Other anxiety disorders
F410	Panic disorder (episodic paroxysmal anxiety)
F411	Generalized anxiety disorder
F413	Other mixed anxiety disorders
F418	Other specified anxiety disorders
F419	Anxiety disorder, unspecified
F930	Separation anxiety disorder of childhood
F931	Phobic anxiety disorder of childhood
F932	Social anxiety disorder of childhood
F412	Mixed anxiety and depressive disorder

The RECORD statement – checklist of items, extended from the STROBE statement, that should be reported in observational studies using routinely collected health data.

	Item No.	STROBE items	Location in manuscript where items are reported	RECORD items	Location in manuscript where items are reported
**Title and abstract**					
	1	(a) Indicate the study's design with a commonly used term in the title or the abstract (b) Provide in the abstract an informative and balanced summary of what was done and what was found	(a) The study design is indicated as a data linkage study in the title (b) We have provided an informative and balanced summary in the abstract	RECORD 1.1: The type of data used should be specified in the title or abstract. When possible, the name of the databases used should be included.<br><br>RECORD 1.2: If applicable, the geographic region and timeframe within which the study took place should be reported in the title or abstract.<br><br>RECORD 1.3: If linkage between databases was conducted for the study, this should be clearly stated in the title or abstract.	1.1 The type of data used is specified in the abstract. It was not possible to name all of the databases used in the abstract.<br>1.2 We have reported the geographic region within which the study took place in the title and the abstract, and the timeframe in the abstract<br>1.3 It is clearly stated in the title and the abstract that linkage between databases was conducted for the study
**Introduction**					
Background rationale	2	Explain the scientific background and rationale	The scientific background and rationale for the		

		for the investigation being reported	investigation is explained in the introduction		
Objectives	3	State specific objectives, including any prespecified hypotheses	The objectives are stated in the introduction		
**Methods**					
Study Design	4	Present key elements of study design early in the paper	The study design is reported at the beginning of the methods section, under the subheading "study design and data source"		
Setting	5	Describe the setting, locations, and relevant dates, including periods of recruitment, exposure, follow-up, and data collection	The setting, locations, and relevant dates are described in the "study population and setting" section		
Participants	6	*(a) Cohort study* - Give the eligibility criteria, and the sources and methods of selection of participants. Describe methods of follow-up<br>*Case-control study* - Give the eligibility criteria, and the sources and methods of case ascertainment and control selection. Give the rationale for the choice of cases and controls	(a) Eligibility criteria, and the sources and methods of selection and follow-up of participants are given in the "study population and setting" section<br>(b) N/A this was not a matched study	RECORD 6.1: The methods of study population selection (such as codes or algorithms used to identify subjects) should be listed in detail. If this is not possible, an explanation should be provided.<br><br>RECORD 6.2: Any validation studies of the codes or algorithms used to select the population should be referenced. If validation was conducted for this study and not	6.1 We have described in detail the methods of study population selection in the "study population and setting" section and the "measures" section<br><br>6.2 We have referenced the validation studies of the anxiety and

		Cross-sectional study - Give the eligibility criteria, and the sources and methods of selection of participants<br><br>*(b) Cohort study* - For matched studies, give matching criteria and number of exposed and unexposed<br>*Case-control study* - For matched studies, give matching criteria and the number of controls per case		published elsewhere, detailed methods and results should be provided.<br><br>RECORD 6.3: If the study involved linkage of databases, consider use of a flow diagram or other graphical display to demonstrate the data linkage process, including the number of individuals with linked data at each stage.	depression codes we have used in our study in the "measures" section<br><br>6.3 We have provided a flow diagram with the number of individuals included in each cohort at each stage of linkage (Figure 1)
Variables	7	Clearly define all outcomes, exposures, predictors, potential confounders, and effect modifiers. Give diagnostic criteria, if applicable.	We have clearly defined all relevant measures in the "measures" section	RECORD 7.1: A complete list of codes and algorithms used to classify exposures, outcomes, confounders, and effect modifiers should be provided. If these cannot be reported, an explanation should be provided.	7.1 We have provided a list of codes used in the study in the appendix
Data sources/ measurement	8	For each variable of interest, give sources of data and details of methods of assessment (measurement). Describe comparability of assessment methods if there is more than one group	We have described all data sources and measures in the "study design and data sources" section and the "measures" section		

Bias	9	Describe any efforts to address potential sources of bias	We have explained that we used a conservative approach to identify cohort participants in the "study population and setting" section		
Study size	10	Explain how the study size was arrived at	We have included a flowchart (Figure 1) to describe the creation of each of the study cohorts and explained the process of cohort creation, referred to in the "study population and setting" section		
Quantitative variables	11	Explain how quantitative variables were handled in the analyses. If applicable, describe which groupings were chosen, and why	Handling of quantitative variables is described in the "measures" section		
Statistical methods	12	(a) Describe all statistical methods, including those used to control for confounding<br>(b) Describe any methods used to examine subgroups and interactions<br>(c) Explain how missing data were addressed<br>(d) *Cohort study* - If applicable, explain how	(a) Statistical methods are described in the "statistical analysis" section<br>(b) N/A<br>(c) N/A<br>(d) N/A<br>(e) Sensitivity analyses are described in the appendix		

Cowley LE, *et al. BMJ Open* 2024; 13:e067882. doi: 10.1136/bmjopen-2022-067882

		loss to follow-up was addressed<br>*Case-control study* - If applicable, explain how matching of cases and controls was addressed<br>*Cross-sectional study* - If applicable, describe analytical methods taking account of sampling strategy<br>(e) Describe any sensitivity analyses			
Data access and cleaning methods		..		RECORD 12.1: Authors should describe the extent to which the investigators had access to the database population used to create the study population.<br><br>RECORD 12.2: Authors should provide information on the data cleaning methods used in the study.	12.1 We described the extent to which the investigators had access to the database population in the "data access and cleaning methods" section<br>12.2 We provided information on the data cleaning methods in the "data access and cleaning methods" section
Linkage		..		RECORD 12.3: State whether the study included person-level, institutional-level, or other data linkage across two or more databases. The methods of	12.3 The methods of linkage and quality evaluation are detailed in the "study design and data

				linkage and methods of linkage quality evaluation should be provided.	source" section and the "study population and setting" section and the flowchart (Figure 1)
**Results**					
Participants	13	(a) Report the numbers of individuals at each stage of the study (*e.g.*, numbers potentially eligible, examined for eligibility, confirmed eligible, included in the study, completing follow-up, and analysed)<br>(b) Give reasons for non-participation at each stage.<br>(c) Consider use of a flow diagram	(a) Numbers of individuals at each study stage are provided in the flow diagrams (Figure 1)<br>(b) Reasons for exclusion are provided on the flowchart (Figure 1)<br>(c) We used a flowchart to illustrate selection of our study cohorts (Figure 1)	RECORD 13.1: Describe in detail the selection of the persons included in the study (*i.e.,* study population selection) including filtering based on data quality, data availability and linkage. The selection of included persons can be described in the text and/or by means of the study flow diagram.	13.1 We described in detail the selection of the persons included in the study in the "study population and setting" section and Figure 1
Descriptive data	14	(a) Give characteristics of study participants (*e.g.*, demographic, clinical, social) and information on exposures and potential confounders<br>(b) Indicate the number of participants with missing data for each variable of interest	(a) Characteristics of study participants are given in Table 2<br>(b) N/A<br>(c) N/A		

		(c) *Cohort study* - summarise follow-up time (*e.g.*, average and total amount)			
Outcome data	15	*Cohort study* - Report numbers of outcome events or summary measures over time<br>*Case-control study* - Report numbers in each exposure category, or summary measures of exposure<br>*Cross-sectional study* - Report numbers of outcome events or summary measures	Numbers of outcome events are reported in the results section in Table 4		
Main results	16	(a) Give unadjusted estimates and, if applicable, confounder-adjusted estimates and their precision (e.g., 95% confidence interval). Make clear which confounders were adjusted for and why they were included<br>(b) Report category boundaries when continuous variables were categorized	(a) We report unadjusted and adjusted estimates in the results section, and have explained why we adjusted for each factor<br>(b) N/A<br>(c) N/A		

		(c) If relevant, consider translating estimates of relative risk into absolute risk for a meaningful time period			
Other analyses	17	Report other analyses done—e.g., analyses of subgroups and interactions, and sensitivity analyses	We report on sensitivity analyses in the appendix		
**Discussion**					
Key results	18	Summarise key results with reference to study objectives	Key results are summarised at the beginning of the discussion		
Limitations	19	Discuss limitations of the study, taking into account sources of potential bias or imprecision. Discuss both direction and magnitude of any potential bias	Limitations are discussed in the 'Strengths and limitations' section including potential underestimation of our cohorts	RECORD 19.1: Discuss the implications of using data that were not created or collected to answer the specific research question(s). Include discussion of misclassification bias, unmeasured confounding, missing data, and changing eligibility over time, as they pertain to the study being reported.	We discussed the limitations of the Shielded Patient List in the 'Strengths and Limitations' section
Interpretation	20	Give a cautious overall interpretation of results considering objectives, limitations, multiplicity of analyses, results from	We have given a cautious and balanced interpretation of the results with reference to the previous literature in the discussion		

Cowley LE, *et al. BMJ Open* 2024; 13:e067882. doi: 10.1136/bmjopen-2022-067882

		similar studies, and other relevant evidence			
Generalisability	21	Discuss the generalisability (external validity) of the study results	Discussed in the "strengths and limitations" section		
**Other Information**					
Funding	22	Give the source of funding and the role of the funders for the present study and, if applicable, for the original study on which the present article is based	We have provided the funding source, and the role of the funders in the "Role of the funders" section		
Accessibility of protocol, raw data, and programming code		..		RECORD 22.1: Authors should provide information on how to access any supplemental information such as the study protocol, raw data, or programming code.	We have provided information on how to access data within the SAIL databank

*Reference: Benchimol EI, Smeeth L, Guttmann A, Harron K, Moher D, Petersen I, Sørensen HT, von Elm E, Langan SM, the RECORD Working Committee. The REporting of studies Conducted using Observational Routinely-collected health Data (RECORD) Statement. *PLoS Medicine* 2015; in press.

*Checklist is protected under Creative Commons Attribution (CC BY) license.

Demographic characteristics of the 2019 study population

		**General population 2019**	**CEV children 2019**	Chi^2 P value
N		438,924	599	
**Sex (%)**	Male	224,754 (51.2)	347 (57.9)	<0.001
	Female	214,170 (48.8)	252 (42.1)	
**Age group (%)**	2–7	162,730 (37.1)	231 (38.6)	0.412
	8–12	143,844 (32.8)	181 (30.2)	
	13–17	132,350 (30.2)	187 (31.2)	
**Deprivation quintile (WIMD 2019) (%)**	1 (most deprived)	111,381 (25.4)	134 (22.4)	0.003
	2	93,046 (21.2)	137 (22.9)	
	3	77,600 (17.7)	88 (14.7)	
	4	74,288 (16.9)	94 (15.7)	
	5 (least deprived)	82,609 (18.8)	146 (24.4)	
**Rural/Urban area (%)**	Rural	118,418 (27.0)	165 (27.5)	0.790
	Urban	320,506 (73.0)	434 (72.5)	
**Any history of anxiety or depression**	NO	421,659 (96.1)	565 (94.3)	0.037
	YES	17,265 (3.9)	34 (5.7)	

Cowley LE, *et al. BMJ Open* 2024; 13:e067882. doi: 10.1136/bmjopen-2022-067882
