# Supplementary material for: Effects of the COVID-19 pandemic on the mental health of clinically extremely vulnerable children and children living with clinically extremely vulnerable people in Wales: a data linkage study
Source: BMJ Open. 2023 Jun 16;13(6):e067882. doi: 10.1136/bmjopen-2022-067882 (PMC10276955; doi:10.1136/bmjopen-2022-067882)
Supplement: Author's manuscript [file bmjopen-2022-067882.draft_revisions.pdf]

BMJ Open is committed to open peer review. As part of this commitment we make the peer review history of every article we publish publicly available.

When an article is published we post the peer reviewers' comments and the authors' responses online. We also post the versions of the paper that were used during peer review. These are the versions that the peer review comments apply to.

The versions of the paper that follow are the versions that were submitted during the peer review process. They are not the versions of record or the final published versions. They should not be cited or distributed as the published version of this manuscript.

BMJ Open is an open access journal and the full, final, typeset and author-corrected version of record of the manuscript is available on our site with no access controls, subscription charges or pay-per-view fees (http://bmjopen.bmj.com).

If you have any questions on BMJ Open's open peer review process please email info.bmjopen@bmj.com

BMJ Open

Effects of the COVID-19 pandemic on the mental health of clinically extremely vulnerable children and children living with clinically extremely vulnerable people in Wales: A data linkage study

Journal:	*BMJ Open*
Manuscript ID	bmjopen-2022-067882
Article Type:	Original research
Date Submitted by the Author:	01-Sep-2022

Complete List of Authors:	Cowley, Laura; Swansea University, Population Data Science<br>Hodgson, Karen; Public Health Wales, Research and Evaluation Division<br>Song, Jiao; Public Health Wales, Health Protection Division<br>Whiffen, Tony; Welsh Government, Administrative Data Research Unit<br>Tan, Jacinta; University of Oxford, Department of Psychiatry<br>John, Ann; Swansea University, Population Data Science<br>Bandyopadhyay, Amrita; Swansea University, National Centre for Population Health and Wellbeing Research<br>Davies, Alisha; Public Health Wales, Research and Evaluation Division

Keywords:	COVID-19, MENTAL HEALTH, Child & adolescent psychiatry < PSYCHIATRY, PUBLIC HEALTH, Depression & mood disorders < PSYCHIATRY, Anxiety disorders < PSYCHIATRY

SCHOLARONE™
Manuscripts

I, the Submitting Author has the right to grant and does grant on behalf of all authors of the Work (as defined in the below author licence), an exclusive licence and/or a non-exclusive licence for contributions from authors who are: i) UK Crown employees; ii) where BMJ has agreed a CC-BY licence shall apply, and/or iii) in accordance with the terms applicable for US Federal Government officers or employees acting as part of their official duties; on a worldwide, perpetual, irrevocable, royalty-free basis to BMJ Publishing Group Ltd ("BMJ") its licensees and where the relevant Journal is co-owned by BMJ to the co-owners of the Journal, to publish the Work in this journal and any other BMJ products and to exploit all rights, as set out in our licence.

The Submitting Author accepts and understands that any supply made under these terms is made by BMJ to the Submitting Author unless you are acting as an employee on behalf of your employer or a postgraduate student of an affiliated institution which is paying any applicable article publishing charge ("APC") for Open Access articles. Where the Submitting Author wishes to make the Work available on an Open Access basis (and intends to pay the relevant APC), the terms of reuse of such Open Access shall be governed by a Creative Commons licence – details of these licences and which Creative Commons licence will apply to this Work are set out in our licence referred to above.

Other than as permitted in any relevant BMJ Author's Self Archiving Policies, I confirm this Work has not been accepted for publication elsewhere, is not being considered for publication elsewhere and does not duplicate material already published. I confirm all authors consent to publication of this Work and authorise the granting of this licence.

For peer review only

Effects of the COVID-19 pandemic on the mental health of clinically extremely vulnerable children and children living with clinically extremely vulnerable people in Wales: A data linkage study

Laura E Cowley[1], PhD, Karen Hodgson[2], PhD, Jiao Song[3], PhD, Tony Whiffen[4], MSc, Jacinta Tan[5], PhD, Prof Ann John[1], PhD, Amrita Bandyopadhyay[6], MSc, Prof Alisha R Davies[2,6], PhD

Affiliations:
[1] Population Data Science, Swansea University Medical School, Wales, United Kingdom
[2] Research and Evaluation Division, Public Health Data, Knowledge and Research Directorate, Public Health Wales, Cardiff, Wales, United Kingdom
[3] Communicable Disease Surveillance Centre, Health Protection Division, Public Health Wales, Cardiff, Wales, United Kingdom
[4] Administrative Data Research Unit, Welsh Government, Wales, United Kingdom
[5] Department of Psychiatry, University of Oxford
[6] National Centre for Population Health and Wellbeing Research, Swansea University Medical School, Wales, United Kingdom

Address correspondence to: Dr Laura Cowley, Data Science Building, Singleton Park, Swansea, SA2 8PP, Wales, United Kingdom. E-mail: l.e.cowley@swansea.ac.uk

Author email addresses: l.e.cowley@swansea.ac.uk; Karen.Hodgson@wales.nhs.uk; Jiao.Song@wales.nhs.uk; Anthony.Whiffen@gov.wales; Jacinta.Tan@oxfordhealth.nhs.uk; A.John@swansea.ac.uk; A.Bandyopadhyay@swansea.ac.uk; Alisha.davies@wales.nhs.uk

Word count: 3,614

For peer review only - http://bmjopen.bmj.com/site/about/guidelines.xhtml

Abstract

Objectives: To determine whether clinically extremely vulnerable (CEV) children or children living with a CEV person in Wales were at greater risk of presenting with anxiety or depression in primary or secondary care during the COVID-19 pandemic compared with children in the general population, and to compare patterns of anxiety and depression during the pandemic (23rd March 2020–31st January 2021, referred to as 2020/21) and before the pandemic (March 23rd 2019–January 31st 2020, referred to as 2019/20), between CEV children and the general population.

Design: Population-based cross-sectional cohort study using anonymised, linked, routinely collected health and administrative data held in the Secure Anonymised Information Linkage Databank. CEV individuals were identified using the COVID-19 Shielded Patient List.

Setting: Primary and secondary healthcare settings covering 80% of the population of Wales.

Participants: Children aged 2–17 in Wales: CEV (3,769); living with a CEV person (20,033); or neither (415,009).

Primary outcome measure: First record of anxiety or depression in primary or secondary healthcare in 2019/20 and 2020/21, identified using Read and ICD-10 codes.

Results: A Cox regression model adjusted for demographics and history of anxiety or depression revealed that only CEV children were at greater risk of presenting with anxiety or depression during the pandemic compared with the general population (Hazard Ratio=2.27, 95% Confidence Interval=1.94–2.66, $p<0.001$). Compared with the general population, the risk amongst CEV children was higher in 2020/21 (Risk Ratio 3.04) compared with 2019/20 (Risk Ratio 1.90). In 2020/21, the cumulative incidence of anxiety or depression increased slightly amongst CEV children, but declined amongst the general population.

Conclusions: Differences in the cumulative incidences of recorded anxiety or depression in healthcare between CEV children and the general population were largely driven by a reduction in presentations to healthcare services by children in the general population during the pandemic.

Strengths and limitations of this study

- Strengths of this study include its novelty, national focus and clinical relevance; to date this is the first population-based study examining the effects of the COVID-19 pandemic on healthcare use for anxiety or depression amongst clinically extremely vulnerable (CEV) children and children living with a CEV person in Wales
- We compared 2020/21 data with pre-pandemic 2019/20 data for CEV children and children in the general population, to place the impact of the COVID-19 pandemic in the context of longer-term patterns of healthcare use
- We used a novel approach and linked multiple datasets to identify a cohort of children living with a CEV person in Wales during the COVID-19 pandemic
- There was heterogeneity within the Shielded Patient List that was used to create the cohorts of children identified as CEV or living with a CEV person, in terms of the type and severity of individuals' underlying conditions; the manner in which people

For peer review only - http://bmjopen.bmj.com/site/about/guidelines.xhtml

were added to the list; the time point that people were added to the list; and the extent to which people followed the shielding guidance

- Routinely collected healthcare data does not capture self-reported health, and is likely to underestimate the burden of common mental disorders in the population

Keywords: COVID-19, mental health, child and adolescent psychiatry, public health, depression and mood disorders, anxiety disorders

For peer review only

For peer review only - http://bmjopen.bmj.com/site/about/guidelines.xhtml

Introduction

In March 2020, Welsh Government and the Welsh National Health Service sought to protect people deemed clinically extremely vulnerable (CEV) to severe illness or death from COVID-19, advising them to 'shield' at home i.e. to remain indoors and minimise contact with others.[1] To identify CEV people, a Shielded Patient List (SPL) was created, using an algorithm based on clinical code lists and applied centrally to patients' electronic health records.[2] Additionally, health professionals could add people to the list based on their clinical judgement. Shielding was in place from 23rd March 2020 until 16th August 2020 and re-introduced from 22nd December 2020 until 1st April 2021.[1] CEV children were encouraged to return to school at the end of August 2020 taking into account the low rate of severe disease and death from COVID-19 amongst children, balanced against the harms of a lack of schooling and socialization.[3]

Studies have highlighted the detrimental impact of the COVID-19 pandemic on the mental health of CEV adults, with CEV individuals more likely to report increased depressive symptoms and anxiety[4] and to have a clinical record of anxiety or depression during the pandemic compared with those who were not CEV.[1] Meanwhile, studies have reported decreased diagnoses of mental health conditions in primary care across the population as a whole during the pandemic[5,6] (attributed to a reluctance to seek healthcare, or reduced access to services, rather than a decrease in need). However, there is limited evidence on how children accessed healthcare during the pandemic for their mental health, and no studies focusing on CEV children, or children living with a CEV person.

Children are particularly vulnerable to indirect impacts of the pandemic.[7] Drawing on evidence from longitudinal surveys,[8–10] the Department for Education concluded that

For peer review only - http://bmjopen.bmj.com/site/about/guidelines.xhtml

children's mental health declined during the pandemic, reporting that rates of probable mental health disorders were higher from 2020 than before.[11] The data also highlighted variation in mental health trajectories; children with long-term health conditions were more likely to experience poor mental health during the pandemic.[11] However, the extent to which these trends are attributable to the pandemic, or are a continuation of pre-existing upward trends is unclear.

Almost 5,000 CEV children were living in Wales by July 2020; approximately 3.9% of the Welsh CEV population.[12] Children with chronic illnesses are at increased risk of behavioural and emotional problems and psychiatric disorders compared with their peers,[13] but CEV children may be particularly susceptible to mental health difficulties relative to non-CEV children since the pandemic, due to additional restrictions imposed by shielding guidance, potentially exacerbating loneliness and isolation.[14] CEV children may have also experienced heightened health anxiety due to their potential higher risk of severe illness from COVID-19. Additionally, there were almost 14,400 school-aged children living with a CEV person in June 2020[15] who may be at greater risk of mental health difficulties due to both restrictions to protect the vulnerable members of their household, and fears of causing harm.[16]

We investigated the impact of the COVID-19 pandemic on use of healthcare services for anxiety or depression in Wales, for CEV children, children living with a CEV person, and children in the general population, using routinely collected population-level linked data. The primary aim was to determine whether CEV children or children living with a CEV person were more likely to have a record for anxiety or depression in primary or secondary care during the pandemic compared with children in the general population. The secondary aim was to compare patterns of anxiety or depression in 2019/2020 and 2020/2021 between CEV

For peer review only - http://bmjopen.bmj.com/site/about/guidelines.xhtml

children and the general population, to place the impact of COVID-19 and the shielding guidance in the context of longer-term patterns of healthcare use.

Methods

Study design and data source

This is a population-based cross-sectional cohort study using anonymised, linked, routinely collected health and administrative data for the population of Wales, UK, held in the Secure Anonymised Information Linkage (SAIL) databank (www.saildatabank.com). Within the SAIL databank, encrypted linkage fields are used to link data anonymously from various sources at individual and household level (appendix pp 1–2); known as Anonymised Linking Fields (ALFs) for individuals and Residential Anonymised Linking Fields (RALFs) for residences.[17,18] We used these to link multiple datasets in this study (Table 1). General Practices (GPs) opt-in to providing data to SAIL; currently, SAIL contains primary care data for around 80% of the Welsh population, and the available data are representative of the entire Welsh population with respect to age, sex, and deprivation.[19] The SAIL databank was interrogated using DB2 structured query language (SQL).

Table 1. Datasets used in this study

Dataset	Description
Welsh Demographic Service (WDS) dataset	A register containing demographic information about all individuals registered at a Welsh General Practice (GP)
COVID-19 Shielded People List (CVSP)	A dataset containing information about clinically extremely vulnerable individuals in Wales, including reasons for shielding
Welsh Index of Multiple Deprivation 2019 (WIMD 2019)	A dataset containing deprivation scores corresponding to all Lower Super Output Areas (LSOAs; geographic units comprised of around 1,600 individuals) in Wales[24]
Rural Urban Classification (RUC) dataset	A dataset containing information on urban/rural categories corresponding to all LSOAs in Wales
Annual District Death Extract (ADDE)	A register containing details of all deaths of Welsh residents, including information regarding date and cause of death

For peer review only - http://bmjopen.bmj.com/site/about/guidelines.xhtml

Outpatient Dataset (OPD)	A dataset containing attendance information for all hospital outpatient appointments in Wales
National Community Child Health Database (NCCHD)	A register of children born in Wales, containing data collected at birth, including a maternal ALF to link children with their biological mothers
Patient Episode Database for Wales (PEDW)	A dataset containing attendance and clinical information for all hospital admissions in Wales, including data regarding diagnoses
Care Homes Data (CHD)	A dataset containing residential information about care homes in Wales
Welsh Longitudinal General Practice (WLGP) dataset	A dataset containing attendance and clinical information for all GP interactions including symptoms, diagnoses, and prescriptions

Ethical Approval

This study used anonymised data and therefore did not require National Research Ethics Committee approval. Approval to access and link the data within SAIL was granted by the Information Governance Review Panel under project number 1265 (appendix p 3).

Patient and Public Involvement

The study protocol was presented at the SAIL consumer panel meeting prior to study commencement. This panel consists of members of the public with an interest in data and its uses to improve services and healthcare. The panel provided advice and feedback on the study design from a public perspective.

Data Access and Cleaning Methods

All authors had full access to all the data in the study. Data cleaning included deduplication and restructuring of the SPL prior to cohort creation and analysis, and was undertaken by LEC.

Role of the funding source

For peer review only - http://bmjopen.bmj.com/site/about/guidelines.xhtml

For peer review only

The funders had no role in the study design, data collection, data analysis, interpretation, writing of the report, or the decision to submit the paper for publication.

Study population and setting

We created three study cohorts for 2020: 1) CEV children 2) children living with at least one CEV person and 3) a general population group of children who were *not* identified as CEV or living with a CEV person, along with two further cohorts for 2019 for comparison purposes. Figure 1 shows a flow diagram of the inclusion criteria for each cohort. We included all children aged 2–17 years who were alive, living in Wales and registered with a SAIL-supplying GP on 23rd March 2020; and who had either an exact match on NHS number or demographics (name, date of birth, gender code, and postcode), or a probabilistic match of 90% or greater.[17] We excluded those for whom full demographic or residence data were not available.

Cohort 1: Clinically extremely vulnerable children

Cohort 1 consisted of all children who were identified as CEV (either by algorithm[2] or health professionals) between 23rd March 2020 and 16th August 2020 (N=3,769).

Cohort 2: Children living with at least one clinically extremely vulnerable person

To identify children living with a CEV person, we first identified the RALF for all CEV people in Wales as of 23rd March 2020, including dates of residence. To minimize bias, we then adopted a conservative approach and included 1) children who were recorded as residents at the same RALF on 23rd March 2020 and had an entry date of residence within 6 months of the entry date of the CEV person 2) children born to mothers who were recorded as resident at the same RALF as the CEV person, or 3) children who shared the same

For peer review only - http://bmjopen.bmj.com/site/about/guidelines.xhtml

maternal ALF as a younger CEV child. We excluded 1) care homes[20] and 2) RALFs containing more than ten people[21], with a final 20,033 children in cohort 2.

Cohort 3: Children in the general population

Cohort 3 consisted of all other children who were alive, living in Wales and registered with a SAIL-supplying GP on 23rd March 2020 (N=415,009).

Clinically extremely vulnerable children in Wales in 2019 (pre-COVID-19 CEV children)

To explore longer-term patterns of anxiety or depression amongst children with the conditions included within the shielding guidance, we created a cohort of pre-COVID-19 "CEV" children who had similar health concerns to CEV children in a period prior to 2020. We included children who were alive, living in Wales, and registered with a SAIL-supplying GP one year prior to the introduction of shielding (23rd March 2019) and who had one or more of three of the health condition categories warranting inclusion on the SPL (respiratory illnesses, blood or bone cancer, and immunosuppression therapy). These categories were chosen as they only required the patient to have one of the listed codes within a given time period and therefore children with these conditions could be identified with a high degree of certainty. We used International Classification of Diseases (ICD) version 10 diagnostic codes and OPCS Classification of Interventions and Procedures version 4 codes for these categories, which were taken from the SPL documentation and provided in the appendix (pp 4–5). For comparison purposes we also created a general population cohort of children aged 2–17 who were alive, living in Wales, and registered with a SAIL-supplying GP as of 23rd March 2019. A flow diagram of inclusion criteria for these cohorts is provided in the appendix (p 6). We performed a sensitivity analysis to confirm the validity of this approach (appendix pp 7–9).

For peer review only - http://bmjopen.bmj.com/site/about/guidelines.xhtml

For peer review only

Measures

Outcome of interest: Anxiety or depression

The outcome of interest was the first record of anxiety or depression in primary or secondary healthcare data during the COVID-19 pandemic (i.e. 23rd March 2020–31st January 2021, referred to as 2020/21), and pre-pandemic (March 23rd 2019–January 31st 2020, referred to as 2019/20). We used validated Read v2 codes to identify children with primary care records for anxiety[22] or depression[23] (including diagnoses, symptoms, and prescriptions) in the WLGP dataset, and ICD-10 diagnostic codes to identify children with hospital admissions or outpatient appointments for anxiety or depression in the PEDW and OPD datasets, respectively. Code lists were reviewed by a clinician with expertise in child psychiatry (JT) and are provided in the appendix (pp 10–14).

Covariates: History of anxiety or depression

We used the same process to identify children in the study population with a 'recent' or 'past' history of anxiety or depression, defined as any record in the year prior to the pandemic (23rd March 2019–23rd March 2020), and any record occurring any time before 23rd March 2019 respectively.[1]

Covariates: Demographics

We calculated age and determined Lower-layer Super Output Areas (LSOA) as of 23rd March 2020. LSOA codes were derived from the WDS dataset based on the child's RALF, and used to ascertain deprivation quintiles and urban/rural classification by linking to the WIMD 2019[24] and RUC datasets.

For peer review only - http://bmjopen.bmj.com/site/about/guidelines.xhtml

Statistical analysis

We used R version 4.1.1 for statistical analyses. P-values of <0.05 were considered statistically significant.

Examining risk of anxiety or depression between different populations in 2020/21

We tested the hypothesis that there was no difference in the risk of having a record of anxiety or depression in 2020/21 between the three cohorts (CEV children, children living with a CEV person, and children in the general population). We plotted Kaplan-Meier survival curves for each cohort. We used Cox regression to calculate unadjusted and adjusted hazard ratios (HRs) with 95% confidence intervals (CI). We report three models examining the risk of anxiety or depression compared with the general population; (1) unadjusted, (2) adjusted for demographic factors (age group, sex, deprivation and rurality) and (3) adjusted for demographic factors and previous history of anxiety or depression (no history, recent history, past history, or both recent and past history).

Examining risk of anxiety or depression between different populations in 2019/20

We tested the hypothesis that there was no difference in the risk of having a record of anxiety or depression in 2019/20 between two cohorts ("CEV" children and all other children living in Wales in 2019). As above, we calculated unadjusted and adjusted HRs, and calculated the change in cumulative incidences of depression and anxiety for these two groups between 2019 and 2020.

Study reporting

This study is reported in accordance with the Reporting of Studies conducted using Observational Routinely-collected data (RECORD) guidelines[25] (appendix pp 15–23).

For peer review only - http://bmjopen.bmj.com/site/about/guidelines.xhtml

Results

Descriptive statistics and demographic characteristics of the study population

Demographic characteristics of the 2020/21 study population are presented in Table 2, and for the 2019/20 study population in the appendix (p 24). For both years, there were greater proportions of boys, older children (aged 13–17), children living in the least and most deprived quintiles and children with a history of anxiety or depression in the CEV children than for the general population. In 2020/21, a greater proportion of children living with a CEV person were older (aged 13–17), and had a history of anxiety or depression, compared with the general population (Table 2). The conditions leading to children being identified as CEV in 2020/21 are shown in Table 3.

For peer review only - http://bmjopen.bmj.com/site/about/guidelines.xhtml

Table 2. Characteristics of clinically extremely vulnerable (CEV) children, children living with a CEV person, and a general population group of children neither CEV or living with a CEV person, 2020/21, Wales

		General population	Clinically extremely vulnerable children	χ^2 statistic, degrees of freedom, and p value, general population v. clinically extremely vulnerable children	Children living with a clinically extremely vulnerable person	χ^2 statistic, degrees of freedom, and p value, general population v. children living with a clinically extremely vulnerable person
N		415,009	3,769		20,033	
**Sex (%)**	Male	212,311 (51·2)	2,184 (57·9)	χ^2=68·6, df=1, p<0·001	10,247 (51·2)	χ^2=0·0, df=1, p=0·989
	Female	202,698 (48·8)	1,585 (42·1)		9,786 (48·8)	
**Age group (years) (%)**	2–7	150,999 (36·4)	1,141 (30·3)	χ^2=84·0, df=2, p<0·001	6,692 (33·4)	χ^2=93·7, df=2, p<0·001
	8–12	137,310 (33·1)	1,247 (33·1)		6,678 (33·3)	
	13–17	126,700 (30·5)	1,381 (36·6)		6,663 (33·3)	
**Deprivation quintile (WIMD 2019) (%)**	1	106,075 (25·6)	1,014 (26·9)	χ^2=11·1, df=4, p=0·025	5,058 (25·2)	χ^2=10·8, df=4, p=0·029
	2	87,930 (21·2)	773 (20·5)		4,107 (20·5)	
	3	73,106 (17·6)	635 (16·8)		3,611 (18·0)	
	4	69,789 (16·8)	589 (15·6)		3,488 (17·4)	
	5	78,109 (18·8)	758 (20·1)		3,769 (18·8)	
**Rural/Urban area (%)**	Rural	110,682 (26·7)	971 (25·8)	χ^2=1·5, df=1, p=0·217	5,444 (27·2)	χ^2=2·5, df=1, p=0·116
	Urban	304,327 (73·3)	2,798 (74·2)		14,589 (72·8)	
**Any history of anxiety or depression (%)**	YES	17,986 (4·3)	368 (9·8)	χ^2=261·5, df=1, p<0·001	1,131 (5·6)	χ^2=78·0, df=1, p<0·001
	NO	397,023 (95·7)	3,401 (90·2)		18,902 (94·4)	

For peer review only - http://bmjopen.bmj.com/site/about/guidelines.xhtml

Table 3. Proportion of clinically extremely vulnerable children with different conditions contributing to underlying reasons to shield

Reason for shielding	Number and percentage of clinically extremely vulnerable children (n = 3,769)
Rare diseases	1,227 (32·6%)
Organ disease	735 (19·5%)
Respiratory illness	660 (17·5%)
Immunosuppression therapy	491 (13%)
Cancer	147 (3·9%)
Transplant	132 (3·5%)
Renal dialysis	10 (0·3%)
GP referred *(reason unknown)*	582 (15·4%)
Other *(reason unknown)*	22 (0·6%)

In children whose reasons for shielding were known (3,165/3,769) 6.3% (198/3,165) had more than one condition

Risk of anxiety or depression in children in 2020/21

Numbers of censored children in each group (due to death, migration or registration with a non-SAIL supplying GP) and numbers with a record for anxiety or depression are presented in Table 4. Of those with a record, 5,768/6,251 (92.3%) presented to primary care while 483/6,251 (7.7%) presented to secondary care.

Table 4. Number of children who were censored, with no event, or who had a record of anxiety or depression during 2020/21

	General population	Clinically extremely vulnerable children	Children living with a clinically extremely vulnerable person
N	415009	3769	20033
Died (%)	12 (0·003)	6 (0·2)	0 (0·0)
Moved out of Wales (%)	7297 (1·8)	68 (1·8)	245 (1·2)
Moved to a non SAIL-supplying GP (%)	5664 (1·4)	31 (0·8)	262 (1·3)

For peer review only - http://bmjopen.bmj.com/site/about/guidelines.xhtml

No event (%)	396267 (95·5)	3505 (93·0)	19203 (95·9)
Anxiety or depression during the pandemic 2020/21 (%)	5,769 (1·4)	159 (4·2)	323 (1·6)

Unadjusted and adjusted hazard ratios are presented in Table 5. In the unadjusted model (1), both CEV children and children living with a CEV person were at significantly greater risk of anxiety or depression compared with the general population (HR=3·09, 95% CI=2·64–3·61, p<0·001 and HR=1·16, 95% CI=1·04–1·30, p<0·05, respectively). For CEV children, the hazard ratio remained significant in both model (2) and (3). However, for children living with a CEV person, the hazard ratio was no longer significant in either model (2) or (3). Full results of the adjusted models are included in the appendix, pp. 25–26. The unadjusted survival curves for each cohort are shown in Figure 2.

For peer review only

For peer review only - http://bmjopen.bmj.com/site/about/guidelines.xhtml

Table 5

Unadjusted and adjusted hazard ratios for having a record of anxiety or depression between 23rd March 2020 and 31st January 2021 (2020/21)

Model	Clinically extremely vulnerable children compared to the general population			Children living with a clinically extremely vulnerable person compared to the general population		
	Hazard Ratio	95% CI	P value	Hazard Ratio	95% CI	P value
Unadjusted (model 1)	3·09	2·64–3·61	p <0·001	1·16	1·04–1·30	p <0·05
Adjusted for demographics: age, sex, deprivation, rurality (model 2)	2·81	2·40–3·29	p <0·001	1·09	0·97–1·22	p = 0·14
Adjusted for demographics and previous clinical history of anxiety or depression (model 3)	2·27	1·94–2·66	p <·001	1·02	0·91–1·14	p = 0·746

For peer review only

Risk of anxiety or depression in children in 2019/2020

Unadjusted and adjusted hazard ratios for anxiety or depression among the "CEV" children as compared with the general population between March 23rd 2019–January 31st 2020 are presented in Table 6. In 2019/20, in the unadjusted model (1) there was an increased risk of anxiety or depression amongst the "CEV" children compared with the general population (HR=1·94, 95% CI=1·31–2·87, p<0·001). This remained evident in the adjusted models (2) and (3). Full results of the adjusted models are included in the appendix, pp. 27–28. The unadjusted survival curves for each cohort are shown in Figure 3.

For peer review only

For peer review only - http://bmjopen.bmj.com/site/about/guidelines.xhtml

Table 6

Unadjusted and adjusted hazard ratios for having a record of anxiety or depression between March 23rd 2019–January 31st 2020 (2019/2020)

**Model**	**"Clinically extremely vulnerable children" compared to the general population**		
	**Hazard Ratio**	**95% CI**	**P value**
**Unadjusted (model 1)**	1·94	1·31–2·87	p <0·001
**Adjusted for demographics: age, sex, deprivation, rurality (model 2)**	2·03	1·37–3·01	p <0·001
**Adjusted for demographics and previous clinical history of anxiety or depression (model 3)**	2·03	1·37–3·01	p <0·001

For peer review only - http://bmjopen.bmj.com/site/about/guidelines.xhtml

Difference in the risk of records of anxiety or depression between 2019/20 and 2020/21

In 2019/20, "CEV" children had increased risk of having recorded anxiety or depression compared with children in the general population, and in 2020/21 the risk ratio increased to 3.04 (Table 7). This reflects a marked decline in presentation amongst children in the general population over this period (from 2·19% to 1·39%), alongside a small increase for CEV children (from 4·17% to 4·22%).

For peer review only

For peer review only - http://bmjopen.bmj.com/site/about/guidelines.xhtml

Table 7. Risk of records of anxiety or depression in children (aged 2–17 years) who were clinically extremely vulnerable (CEV) and those in the general population, in 2019/20 and 2020/21

Time period	CEV children<br>*2020/21 - children identified through shielded patient list*<br>*2019/20 – comparable cohort of children with the conditions listed in the shielded patient list*			Children in the general population in Wales			Crude Risk Ratio
	**No. children with recorded anxiety or depression**	**Total No. of children**	**Cumulative Incidence (%)**	**No. children with recorded anxiety or depression**	**Total No. of children**	**Cumulative Incidence (%)**	
2019/20 (Pre COVID-19)	25	599	4·17	9,620	438,924	2·19	1·90
2020/21 (During COVID-19)	159	3,769	4·22	5,769	415,009	1·39	3·04
**Percentage point change over time points**			-0·05			0·8	

For peer review only - http://bmjopen.bmj.com/site/about/guidelines.xhtml

Discussion

In Wales, CEV children and children living with a CEV person were more likely to access health services for anxiety or depression during the pandemic than children in the general population. For CEV children, this pattern remained evident after adjusting for demographic differences and the likelihood of having a previous history of anxiety or depression. Although a small increase in risk was found for children living with a CEV person, after adjusting for demographic characteristics and previous history of anxiety and depression, this was no longer significant.

Given the detailed methodology used to identify CEV individuals in Wales,[2] we were able to develop a comparable cohort of children with a subset of health conditions before the pandemic, using routine healthcare data. This enabled us to examine patterns of presentation for anxiety and depression amongst CEV children outside of the context of the pandemic. We found this group were at greater risk of having a record for anxiety or depression compared with children in the general population in 2019/20, before COVID-19. In 2020/21, CEV children remained at higher risk, and the difference was greater, although this is explained by a marked decline amongst children in the general population presenting to healthcare services with anxiety or depression during this time.

The reduction in presentations for anxiety and depression amongst children in the general population most likely reflects reduced access to NHS services during the pandemic. Other evidence suggests increased demand and unmet need for mental health support in the UK, for children with and without pre-existing mental health problems, since 2020.[26,27] Findings from self-report surveys[8–10] and the current study suggest that the pandemic has widened the gap between need and access to mental healthcare for the general population of children in Wales,

For peer review only - http://bmjopen.bmj.com/site/about/guidelines.xhtml

but additional data are required to unpack the relationship between self-reported mental health needs and presentation to healthcare services.

Meanwhile, the relatively stable cumulative incidences of anxiety and depression for CEV children in 2019/20 and 2020/21 could indicate that this group did not experience an increase in mental health needs during the pandemic over and above past years, and that they had access to mental health support through existing care pathways for underlying conditions. Alternatively, if CEV children experienced the same increase in mental health needs as reported elsewhere amongst the general population, then these figures may mask unmet demand for mental health support among this group. A survey of adults supports the latter explanation, reporting increased anxiety and depressive symptoms amongst shielding individuals compared with non-shielding individuals.[4] However, we have found no UK surveys focusing on the mental health of CEV children.

Our finding that children living with a CEV person were at no greater risk of presenting with anxiety or depression during the pandemic compared with the general population (after controlling for other factors), could be interpreted in two ways. It is possible that the impact of the pandemic on the mental health of children living with a CEV person was not as great as for CEV children, but this seems unlikely given that research has suggested increased anxiety amongst children who were shielding their siblings.[16] Another explanation is that children living with a CEV person suffered from similar barriers to access to mental healthcare services to the general population, and did not have the same routes to access that CEV children did. This explanation is supported by research with shielding families, which suggests that they have felt left behind, and that children living with a CEV person may have 'fallen under the radar of educational and healthcare professionals'.[16]

For peer review only - http://bmjopen.bmj.com/site/about/guidelines.xhtml

Strengths and limitations

To our knowledge, this is the first population-based study examining the effects of the COVID-19 pandemic on healthcare use for anxiety or depression amongst CEV children and children living with a CEV person in Wales. Linkage of population-based routinely collected data is a valuable method for generating evidence with a high level of external validity and applicability for policy making. A strength of this study is the comparison of 2020/21 data with pre-pandemic 2019/20 data for CEV children and children in the general population. Another strength is the use of a novel approach using multiple linked datasets to identify a cohort of children living with a CEV person during the pandemic.

This study used the SPL to create cohorts of children identified as CEV and a cohort of children living with a CEV person. There was heterogeneity within the SPL in terms of the type and severity of individuals' underlying conditions; the manner in which people were added to the list (via the algorithm or clinical judgement); the time point that people were added to the list; and the extent to which people followed the shielding guidance. In addition, the impact of following shielding guidance is likely to have varied due to individual circumstances and the level of support received. The 2019 "CEV" cohort was a relatively small sample and for pragmatic reasons, only included children with a subset of the conditions included in the shielding guidance. To identify children living with a CEV person, we adopted a conservative approach and included children only if they were living with their mother. We took this approach in order to minimize bias and increase the generalisability of the findings, however this approach is likely to have underestimated the number of children living with a CEV person. Finally, this study focused on healthcare use using clinical codes. Routinely collected healthcare data does not capture self-reported health, and is likely to underestimate the burden of common mental disorders in the population.[28] Focusing on

For peer review only - http://bmjopen.bmj.com/site/about/guidelines.xhtml

healthcare use with routine data alone cannot tell us about the underlying reasons for changes in utilisation, or the scale of mental health need.

Implications for policy and practice

Our findings have implications for recovery planning to prevent, mitigate and respond to the mental health impacts of the pandemic. We have shown changes in presentation to primary and secondary healthcare services with anxiety and depression for CEV children and children in the general population during the pandemic, and there are concerns regarding potential increases in unmet mental health needs over time. As highlighted by UK organisations such as the Centre for Mental Health,[29] services face challenges in tackling this demand. This has been recognised by Welsh Government, who invested an additional £9.4m in children's mental health services in 2021.[30]

This novel linked data study contributes to our understanding of the direct and indirect impact of shielding on children's mental health in Wales during the COVID-19 pandemic. This evidence should be considered in light of additional, more detailed routine healthcare linkage studies, and national surveys, to provide a comprehensive understanding of the relationship between mental health support needs, expressed demands and care provision to better target services to those who need them the most.

Beyond the indirect impacts of the pandemic, our findings highlight the increased mental health needs of children with serious medical conditions. Given that these children are likely to have greater contact with healthcare services, signposting across services including mental health services, is likely to be beneficial.

For peer review only - http://bmjopen.bmj.com/site/about/guidelines.xhtml

Figure captions

Figure 1. Flow diagram of the inclusion criteria for the creation of three study cohorts: clinically extremely vulnerable children, children living with a clinically extremely vulnerable person, and children in the general population. CEV, clinically extremely vulnerable; CVSP, COVID-19 Shielded People List; SAIL, Secure Anonymised Information Linkage; GP, general practice; RALF, residential anonymised linking field; NHS, national health service.

Figure 2. Kaplan-Meier survival curves for each cohort, showing the time to first record of anxiety or depression between 23rd March 2020 and 31st January 2021 (2020/21)

Figure 3. Kaplan-Meier survival curves for each cohort, showing the time to first record of anxiety or depression between 23rd March 2019 and 31st January 2020 (2019/20)

Funding statement

This work was supported by the Adolescent Mental Health Data Platform (ADP). The ADP is funded by MQ Mental Health Research Charity (Grant Reference MQBF/3 ADP). This work was also supported by the National Centre for Population Health and Wellbeing Research (NCPHWR) which is funded by Health and Care Research Wales.

Acknowledgements

ADP and the authors would like to acknowledge the data providers who supplied the datasets enabling this research study. The views expressed are entirely those of the authors and should not be assumed to be the same as those of ADP or MQ Mental Health Research Charity. This study makes use of anonymised data held in the Secure Anonymised Information Linkage (SAIL) Databank. We would like to acknowledge all the data providers who make anonymised data available for research.

For peer review only

Author contributions

LEC: study design, literature search, data curation, data analysis, figures, data interpretation, writing – original draft, writing – review and editing; KH: data analysis, data interpretation, supervision, writing – review and editing; JS: conceptualisation, study design, methodology, data curation, data analysis, data interpretation, supervision, writing – review and editing; TW: methodology, writing – review and editing; JT: validation, writing – review and editing; AJ: methodology, funding acquisition, writing – review and editing; AB: methodology, writing – review and editing; ARD: conceptualisation, study design, data analysis, data interpretation, supervision, funding acquisition, writing – review and editing. LEC and JS verified the underlying data.

Competing interests' statement

Ann John is a member of the Welsh Government COVID-19 Technical Advisory Group and is also co-chair of the Scientific Pandemic Insights Group on Behaviours (SPI-B) which is a subgroup of the Scientific Advisory Group for Emergencies (SAGE) advising the UK government. None of the other authors have any competing interests.

Data sharing statement

The data used in this study are available in the SAIL Databank at Swansea University (Swansea, UK) via the Adolescent Mental Health Data Platform, but, as restrictions apply, they are not publicly available. All proposals to use SAIL data are subject to review by an independent Information Governance Review Panel (IGRP). Before any data can be accessed, approval must be given by the IGRP. The IGRP carefully considers each project to ensure proper and appropriate use of SAIL data. When access has been granted, it is gained through a privacy-protecting safe haven and remote access system referred to as the SAIL

For peer review only

For peer review only - http://bmjopen.bmj.com/site/about/guidelines.xhtml

Gateway. SAIL have established an application process to be followed by anyone who would like to access data via SAIL, details of which can be found at https://saildatabank.com/data/apply-to-work-with-the-data/

References

1 Davies A, Song J, Bentley L, *et al.* COVID-19 in Wales: The impact on levels of health care use and mental health of the clinically extremely vulnerable. Cardiff: Public Health Wales. 2021. https://phw.nhs.wales/services-and-teams/knowledge-directorate/research-and-evaluation/publications/covid-19-in-wales-the-impact-on-levels-of-health-care-use-and-mental-health-of-the-clinically-extremely-vulnerable/ (accessed March 2, 2022).

2 NHS Wales Informatics Service. COVID-19 high risk shielded patient list identification methodology. 2020. https://nwis.nhs.wales/coronavirus/coronavirus-content/coronavirus-documents/covid-19-high-risk-shielded-patient-list-identification-methodology-v3-2-17th-august/ (accessed Dec 10, 2020).

3 Department of Health and Social Care. Statement from the UK Chief Medical Officers on schools and childcare reopening. 2020. https://www.gov.uk/government/news/statement-from-the-uk-chief-medical-officers-on-schools-and-childcare-reopening (accessed Dec 12, 2020).

4 Di Gessa G, Price D. The impact of shielding during the COVID-19 pandemic on mental health: evidence from the English Longitudinal Study of Ageing. *Br J Psychiatry* 2022; published online April 4. https://doi.org/10.1192/bjp.2022.44.

5 Mansfield KE, Mathur R, Tazare J, *et al.* Indirect acute effects of the COVID-19 pandemic on physical and mental health in the UK: a population-based study. *Lancet Digit Heal* 2021; **3**: e217–30.

6 Carr MJ, Steeg S, Webb RT, *et al.* Effects of the COVID-19 pandemic on primary care-recorded mental illness and self-harm episodes in the UK: a population-based cohort study. *Lancet Public Heal* 2021; **6**: e124–35.

7 Crawley E, Loades M, Feder G, Logan S, Redwood S, Macleod J. Wider collateral damage to children in the UK because of the social distancing measures designed to reduce the impact of COVID-19 in adults. *BMJ Paediatr Open* 2020; **4**: e000701.

8 Newlove-Delgado, T Williams T, Robertson K, McManus S, *et al.* Mental health of children and young people in England, 2021. Wave 2 follow up to the 2017 survey. 2021. https://files.digital.nhs.uk/97/B09EF8/mhcyp_2021_rep.pdf (accessed Dec 15, 2021).

9 Vizard T, Sadler K, Ford T, *et al.* Mental health of children and young people in England, 2020. Wave 1 follow up to the 2017 survey. 2020. https://files.digital.nhs.uk/AF/AECD6B/mhcyp_2020_rep_v2.pdf (accessed April 5, 2021).

10 Raw JAL, Waite P, Pearcey S, Shum A, Patalay P, Creswell C. Examining changes in parent-reported child and adolescent mental health throughout the UK's first COVID-19 national lockdown. *J Child Psychol Psychiatry* 2021; **62**: 1391–401.

11 Department for Education. State of the Nation 2021: Children and young people's

For peer review only - http://bmjopen.bmj.com/site/about/guidelines.xhtml

wellbeing. Research report. 2022. https://assets.publishing.service.gov.uk/government/uploads/system/uploads/attachment_data/file/1053302/State_of_the_Nation_CYP_Wellbeing_2022.pdf (accessed April 7, 2022).
12 Welsh Government. Shielded households (composition and characteristics during the coronavirus (COVID-19) pandemic: July 2020. 2020. https://gov.wales/shielded-households-composition-and-characteristics-during-coronavirus-covid-19-pandemic-july-2020 (accessed Nov 14, 2020).
13 Hysing M, Elgen I, Gillberg C, Lie SA, Lundervold AJ. Chronic physical illness and mental health in children. Results from a large-scale population study. *J Child Psychol Psychiatry* 2007; **48**: 785–92.
14 Griffiths H, O'Connor K, Phillips B, *et al.* Meeting the psychological needs of children in shielding families. 2020. https://www.bps.org.uk/sites/www.bps.org.uk/files/Policy/Policy - Files/Meeting the psychological needs of children in shielding families.pdf (accessed Nov 17, 2020).
15 Welsh Government. Children living in shielded households during the coronavirus (COVID-19) pandemic: June 2020. 2020. https://gov.wales/children-living-shielded-households-during-coronavirus-covid-19-pandemic-june-2020-html (accessed Nov 15, 2020).
16 Kassa C, Pavlopoulou G. Lonely lockdown. Life for siblings of disabled children in the UK. 2021. https://doi.org/10.14324/000.rp.10125424.
17 Lyons RA, Jones KH, John G, *et al.* The SAIL databank: linking multiple health and social care datasets. *BMC Med Inform Decis Mak* 2009; **9**: 3.
18 Rodgers S, Lyons R, Dsilva R, *et al.* Residential Anonymous Linking Fields (RALFs): a novel information infrastructure to study the interaction between the environment and individuals' health. *J Public Health* 2009; **31**: 582–8.
19 Schnier C, Wilkinson T, Akbari A, *et al.* The Secure Anonymised Information Linkage databank Dementia e-cohort (SAIL-DeC). *Int J Popul Data Sci* 2020; **5**: 1121.
20 Schultze A, Bates C, Cockburn J, *et al.* Identifying care home residents in electronic health records - an OpenSAFELY short data report. *Wellcome Open Res* 2021; **6**: 90.
21 Paranjothy S, Evans A, Bandyopadhyay A, *et al.* Risk of emergency hospital admission in children associated with mental disorders and alcohol misuse in the household: an electronic birth cohort study. *Lancet Public Health* 2018; **3**: e279–88.
22 John A, Marchant AL, McGregor JI, *et al.* Recent trends in the incidence of anxiety and prescription of anxiolytics and hypnotics in children and young people: an e-cohort study. *J Affect Disord* 2015; **183**: 134–41.
23 John A, Marchant AL, Fone DL, *et al.* Recent trends in primary-care antidepressant prescribing to children andyoung people: an e-cohort study. *Psychol Med* 2016; **46**: 3315.
24 Welsh Government. Welsh Index of Multiple Deprivation (WIMD) 2019: Results report. 2019. https://gov.wales/sites/default/files/statistics-and-research/2019-11/welsh-index-multiple-deprivation-2019-results-report-024.pdf (accessed Nov 20, 2020).
25 Benchimol EI, Smeeth L, Guttmann A, *et al.* The REporting of studies Conducted using Observational Routinely-collected health Data (RECORD) statement. *PLoS Med* 2015; **12**: e1001885.
26 Torjesen I. Covid-19: Only a third of children in need accessed mental health support in the pandemic. *BMJ* 2022; **376**: o335.
27 Young Minds. Coronavirus: Impact on young people with mental health needs. 2020. https://www.youngminds.org.uk/media/04apxfrt/youngminds-coronavirus-report-

For peer review only - http://bmjopen.bmj.com/site/about/guidelines.xhtml

summer-2020.pdf (accessed Nov 2, 2020).
28 Cornish RP, John A, Boyd A, Tilling K, Macleod J. Defining adolescent common mental disorders using electronic primary care data: A comparison with outcomes measured using the CIS-R. *BMJ Open* 2016; **6**: e013167.
29 Centre for Mental Health. Covid-19 and the nation's mental health: October 2020. 2020. https://www.centreformentalhealth.org.uk/publications/covid-19-and-nations-mental-health-october-2020 (accessed April 8, 2021).
30 Welsh Government. Pledge to support youth with extra £9.4m investment in children and young people mental health services. 2021. https://gov.wales/pledge-support-youth-extra-ps94m-investment-children-and-young-people-mental-health-services (accessed May 2, 2021).

For peer review only

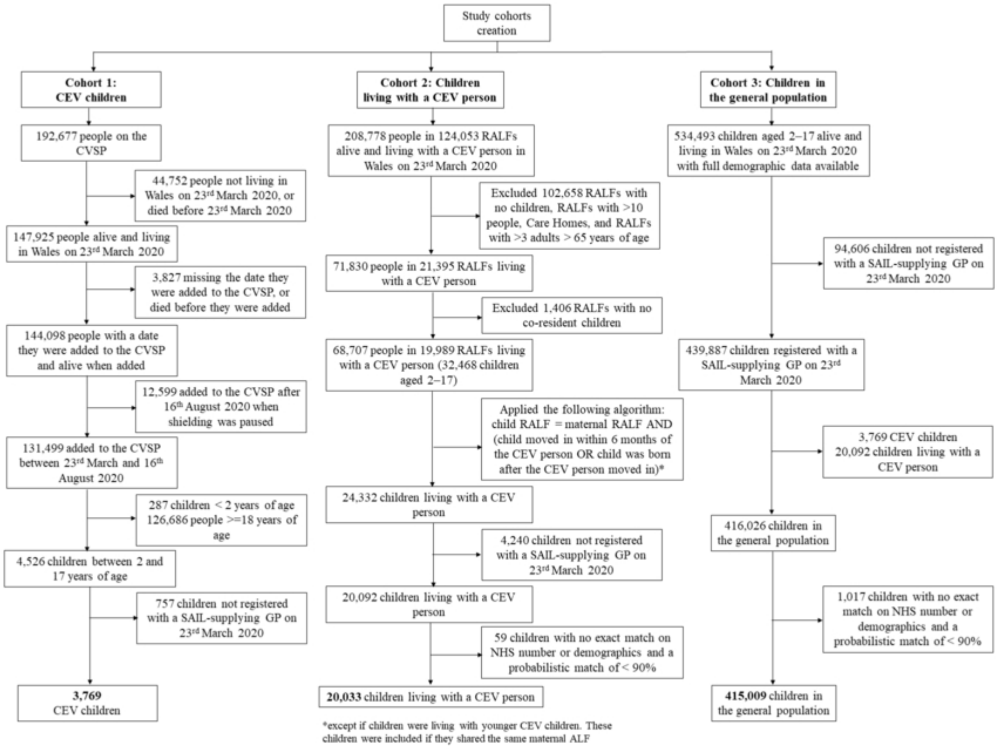

Figure 1. Flow diagram of the inclusion criteria for the creation of three study cohorts: clinically extremely vulnerable children, children living with a clinically extremely vulnerable person, and children in the general population. CEV, clinically extremely vulnerable; CVSP, COVID-19 Shielded People List; SAIL, Secure Anonymised Information Linkage; GP, general practice; RALF, residential anonymised linking field; NHS, national health service.

36x27mm (600 x 600 DPI)

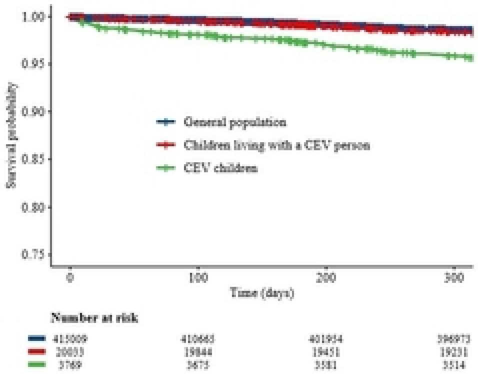

Figure 2. Kaplan-Meier survival curves for each cohort, showing the time to first record of anxiety or depression between 23rd March 2020 and 31st January 2021 (2020/21)

10x8mm (600 x 600 DPI)

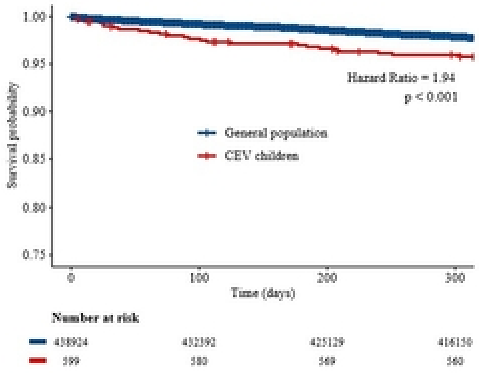

Figure 3. Kaplan-Meier survival curves for each cohort, showing the time to first record of anxiety or depression between 23rd March 2019 and 31st January 2020 (2019/20)

10x8mm (600 x 600 DPI)

Appendix

SAIL databank additional information

SAIL databank information governance policies and procedures

The SAIL databank (www.saildatabank.com) is an internationally recognised, remotely-accessible, privacy-protecting data safe haven designed to support observational, interventional, and policy-relevant research to improve population health, well-being, and services.[1–6] SAIL contains anonymised, linkable, routinely collected health, administrative, and social care data for the population of Wales, UK, from multiple sources at individual, household, and ecological levels.[1–6] Data are anonymised using a split-file process, which has been described in detail elsewhere.[1–3] Within each dataset, identifiable and non-identifiable data are separated, and identifiable data are sent to a trusted third party (TTP), Digital Health and Care Wales (DHCW; previously known as the NHS Wales Informatics Service). The TTP uniquely matches identities based on name, NHS number, date of birth, and Unique Property Reference Number (UPRN), using the Matching Algorithm for Consistent Results in Anonymised Linkage, which has an accuracy of 99.85%.[1,2] Individuals and residences are then assigned unique identifiers: for individuals this is called an Anonymised Linking Field (ALF) and for residences a Residential Anonymised Linking Field (RALF). The anonymised and non-identifiable data components are then recombined within SAIL and the linking fields are further encrypted and used to anonymously link between datasets. This enables data from multiple sources, including general practice (GP) data, hospital admissions, outpatient data, and demographic details to be linked at the individual and household level, while preserving anonymity.

References

For peer review only - http://bmjopen.bmj.com/site/about/guidelines.xhtml

1. Lyons RA, Jones KH, John G, et al. The SAIL databank: linking multiple health and social care datasets. BMC Med Inform Decis Mak2009;9:3.doi:10.1186/1472-6947-9-3
2. Ford DV, Jones KH, Verplancke JP, et al. The SAIL Databank: building a national architecture for e-health research and evaluation. BMC Health Serv Res2009;9:157.doi:10.1186/1472-6963-9-157
3. Rodgers SE, Lyons RA, Dsilva R, et al. Residential Anonymous Linking Fields (RALFs): a novel information infrastructure to study the interaction between the environment and individuals health. J Public Health2009;31:582–8.doi:10.1093/pubmed/fdp041
4. Rodgers SE, Demmler JC, Dsilva R, Lyons RA. Protecting health data privacy while using residence-based environment and demographic data. *Health Place*. 2012; 18: 209-217.
5. Jones, K. H., Ford, D. V., Thompson, S., & Lyons, R. A. (2019). A profile of the SAIL databank on the UK secure research platform. *International Journal of Population Data Science*, *4*(2). https://doi.org/10.23889/ijpds.v4i2.1134
6. Jones, K. H., Ford, D. V., Jones, C., Dsilva, R., Thompson, S., Brooks, C. J., Heaven, M. L., Thayer, D. S., McNerney, C. L., & Lyons, R. A. (2014). A case study of the secure anonymous information linkage (SAIL) gateway: A privacy-protecting remote access system for health-related research and evaluation. *Journal of Biomedical Informatics*, *50*, 196–204. https://doi.org/10.1016/j.jbi.2014.01.003

For peer review only - http://bmjopen.bmj.com/site/about/guidelines.xhtml

Ethical Approval

The Information Governance Review Panel (IGRP) is an independent panel of representatives from various government, regulatory, and professional organisations, who review all proposals for SAIL data access to ensure that they are appropriate with respect to Information Governance, and in the public interest.[1] Data were analysed within the SAIL secure research environment, and appropriate disclosure control procedures were followed to ensure that no personally identifiable data or small numbers (n<5) were removed from the environment. All data within SAIL are treated in accordance with the Data Protection Act 2018 and SAIL complies with the principles of the General Data Protection Regulation (GDPR).

References

1. Ford DV, Jones KH, Verplancke JP, et al. The SAIL Databank: building a national architecture for e-health research and evaluation. BMC Health Serv Res2009;9:157.doi:10.1186/1472-6963-9-157

For peer review only

ICD-10 codes for cancers of the blood or bone marrow

ICD-10 code	Description
C81	Hodgkin lymphoma
C82	Follicular lymphoma
C83	Non-follicular lymphoma
C84	Mature T/NK-cell lymphomas
C85	Other and unspecified types of non-Hodgkin lymphoma
C86	Other specified types of T/NK-cell lymphoma
C88	Malignant immunoproliferative diseases
C90	Multiple myeloma and malignant plasma cell neoplasms
C91	Lymphoid leukaemia
C92	Myeloid leukaemia
C93	Monocytic leukaemia
C94	Other leukaemias of specified cell type
C95	Leukaemia of unspecified cell type
C96	Other and unspecified malignant neoplasms of lymphoid, haematopoietic and related tissue
D470	Histiocytic and mast cell tumours of uncertain and unknown behaviour
D475	Chronic eosinophilic leukaemia [hypereosinophilic syndrome]
D477	Other specified neoplasms of uncertain or unknown behaviour of lymphoid, haematopoietic and related tissue
D479	Neoplasm of uncertain or unknown behaviour of lymphoid, haematopoietic and related tissue, unspecified
D595	Paroxysmal nocturnal haemoglobinuria [Marchiafava-Micheli]
D71X	Functional disorders of polymorphonuclear neutrophils
D730	Hyposplenism
D760	Langerhans' cell histiocytosis, not elsewhere classified
D761	Haemophagocytic lymphohistiocytosis
D898	Other specified disorders involving the immune mechanism, not elsewhere classified
D899	Disorder involving the immune mechanism, unspecified
L412	Lymphomatoid papulosis
P615	Transient neonatal neutropenia

ICD-10 codes for respiratory illnesses

ICD-10 code	Description
E84	Cystic Fibrosis
J84	Other interstitial pulmonary diseases
J620	Pneumoconiosis due to talc dust
J630	Aluminosis (of lung)
J631	Bauxite fibrosis (of lung)
J633	Graphite fibrosis (of lung)
J634	Siderosis
J635	Stannosis

For peer review only - http://bmjopen.bmj.com/site/about/guidelines.xhtml

J660	Byssinosis
J661	Flax-dresser disease
J662	Cannabinosis
J668	Airway disease due to other specific organic dusts
J670	Farmer lung
J671	Bagassosis
J678	Hypersensitivity pneumonitis due to other organic dusts
J684	Chronic respiratory conditions due to chemicals, gases, fumes and vapours
J688	Other respiratory conditions due to chemicals, gases, fumes and vapours
J698	Pneumonitis due to other solids and liquids
J701	Chronic and other pulmonary manifestations due to radiation
J703	Chronic drug-induced interstitial lung disorders
J840	Alveolar and parietoalveolar conditions
J983	Compensatory emphysema
J991	Respiratory disorders in other diffuse connective tissue disorders
M313	Wegener granulomatosis
P250	Interstitial emphysema originating in the perinatal period
Q334	Congenital bronchiectasis

OPCS-4 codes for immunosuppression therapy

OPCS-4 code	Description
X353	Active Inflammatory thyroid eye disease patients currently on weekly intravenous steroid infusion treatment (12 weekly injections regime) or immunospressant
X374	Intramuscular Immunotherapy
X385	Subcutaneous Immunotherapy
X891	Monoclonal antibodies Band 1
X892	Monoclonal antibodies Band 2
X893	Patients receiving maintenance treatment with rituximab, obinotuzimab or ofatumumab
X894	Somatostatin analogues Band 1
X895	Allergic emergency drugs Band 1
X961	Patients previously treated for haematological malignancy requiring IV immunoglobulin replacement
X962	Allergen immunotherapy drugs Band 1
X963	Poison management drugs Band 1

For peer review only - http://bmjopen.bmj.com/site/about/guidelines.xhtml

Pre-COVID-19 study cohorts

Figure 3 shows a flow diagram of inclusion criteria for the two 2019 study cohorts: children in the general population, and clinically extremely vulnerable children with blood or bone cancer or respiratory illnesses, or receiving immunosuppression therapy.

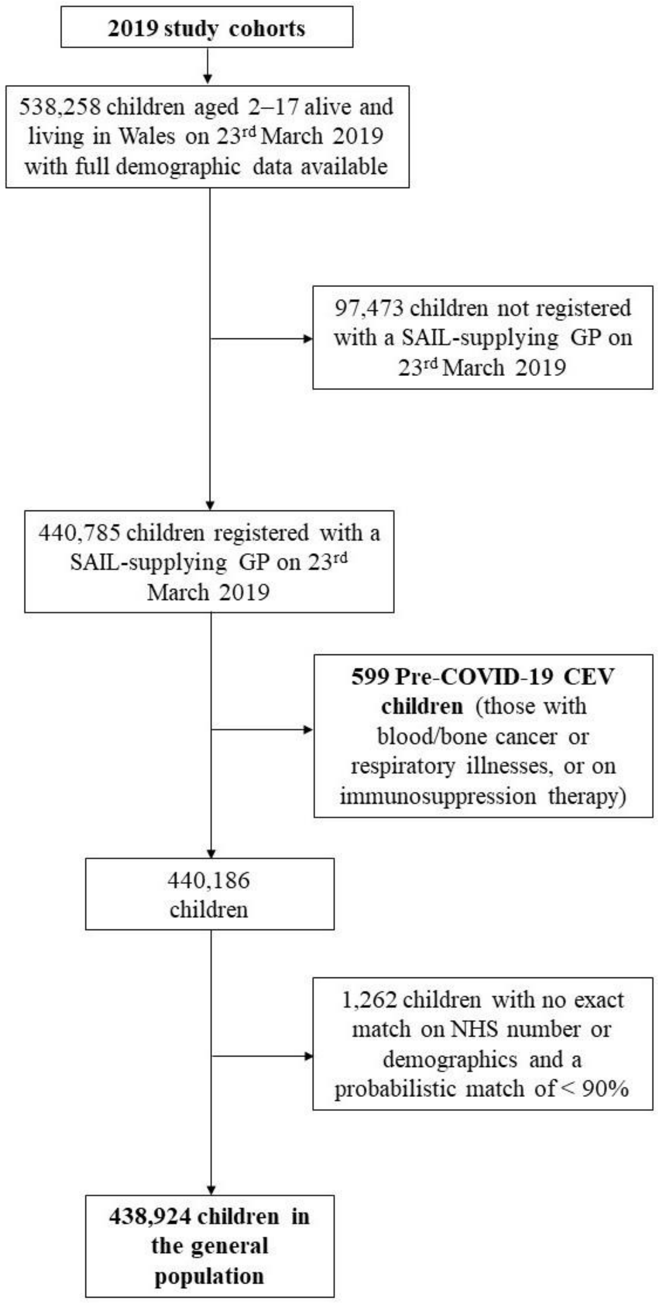

Figure 3. Flow diagram of the inclusion criteria for the creation of the 2019 study cohorts

For peer review only - http://bmjopen.bmj.com/site/about/guidelines.xhtml

Sensitivity Analysis

We undertook a sensitivity analysis to confirm the validity of creating a cohort of pre-COVID-19 CEV children based on just three of the categories included in the COVID-19 Shielded People List (CVSP). We created a cohort of clinically extremely vulnerable (CEV) children who were added to the CVSP in 2020 for the same three reasons only (respiratory illnesses, blood/bone cancer, and immunosuppression therapy) and a cohort of children in the general population (figure 1). Demographic characteristics of the children in each cohort are presented in Table 1. We plotted the Kaplan-Meier survival curve for each cohort and estimated the hazard ratio of having a record for anxiety or depression during the COVID-19 pandemic (March 23rd 2020–January 31st 2021) using Cox regression (figure 2).

For peer review only

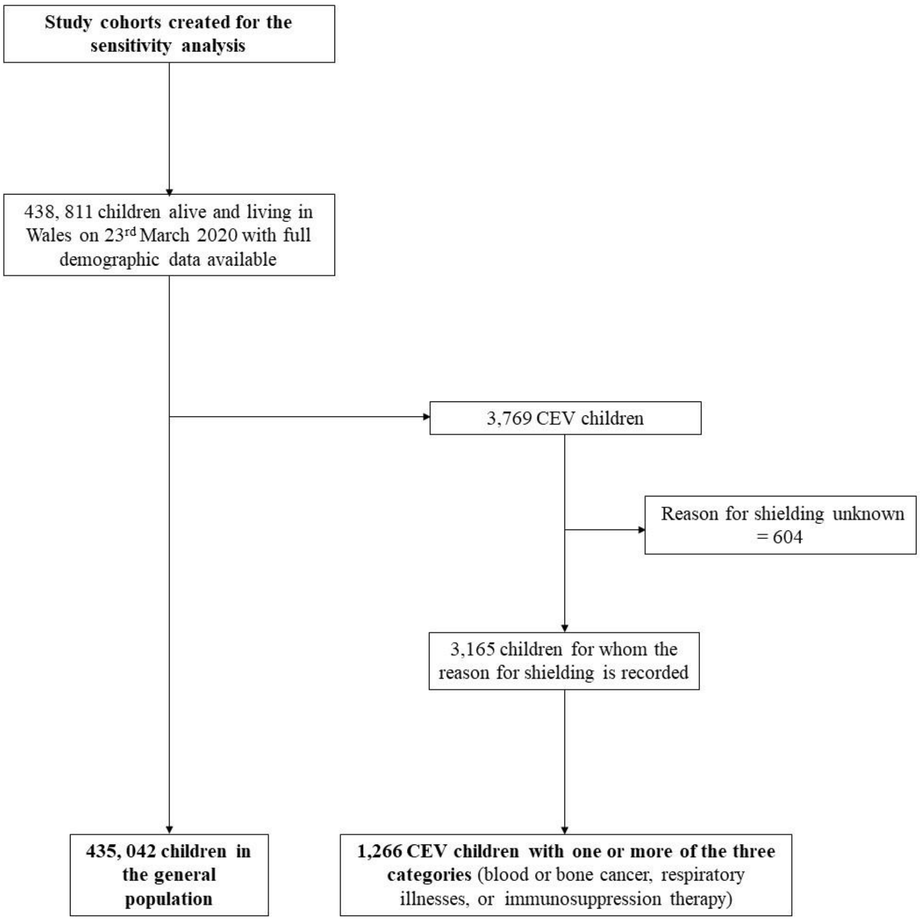

Figure 1. Flow diagram of the inclusion criteria for the creation of the study cohorts for the sensitivity analysis.

For peer review only - http://bmjopen.bmj.com/site/about/guidelines.xhtml

		General population 2020	CEV children with one or more of three categories 2020	Chi^2 P value
N		435,042	1,266	
**Sex (%)**	Male	222,558 (51.2)	712 (56.2)	<0.001
	Female	212,484 (48.8)	554 (43.8)	
**Age group (%)**	2–7	157,691 (36.2)	385 (30.4)	<0.001
	8–12	143,988 (33.1)	396 (31.3)	
	13–17	133,363 (30.7)	485 (38.3)	
**Deprivation quintile (WIMD 2019) (%)**	1 (most deprived)	111,133 (25.5)	329 (26.0)	0.262
	2	92,037 (21.2)	257 (20.3)	
	3	76,717 (17.6)	210 (16.6)	
	4	73,277 (16.8)	203 (16.0)	
	5 (least deprived)	81,878 (18.8)	267 (21.1)	
**Rural/Urban area (%)**	Rural	116,126 (26.7)	336 (26.5)	0.928
	Urban	318,916 (73.3)	930 (73.5)	
**Any history of anxiety or depression**	NO	415,925 (95.6)	1,166 (92.1)	<0.001
	YES	19,117 (4.4)	100 (7.9)	

Table 1. Demographic characteristics of children in the general population and clinically extremely vulnerable children added to the COVID-19 Shielded People List for one or more of three reasons (blood or bone cancer, respiratory illnesses, or immunosuppression therapy)

The results showed a similar pattern to those obtained when analysing the full CEV cohort in that CEV children with one or more of the three categories were at greater risk of having a record for anxiety or depression during the COVID-19 pandemic (March 23rd 2020–January 31st 2021) compared to children in the general population, thereby validating our approach.

For peer review only

For peer review only - http://bmjopen.bmj.com/site/about/guidelines.xhtml

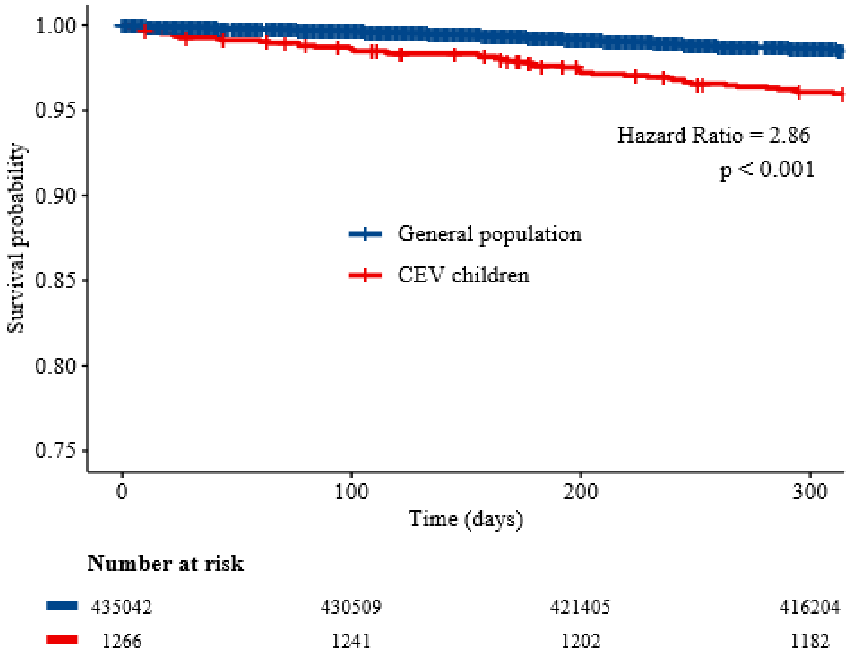

Figure 2. Kaplan-Meier survival curves for each cohort, showing the time to first record of anxiety or depression during the COVID-19 pandemic

CEV, clinically extremely vulnerable

For peer review only - http://bmjopen.bmj.com/site/about/guidelines.xhtml

Read v2 and ICD-10 diagnosis codes used to identify records of anxiety or depression in primary or secondary care

Read v2 codes for depression diagnosis

Read v2 code	Description
Eu32.	[X]Depressive episode
Eu320	[X]Mild depressive episode
Eu321	[X]Moderate depressive episode
Eu322	[X]Severe depressive episode without psychotic symptoms
Eu324	[X]Mild depression
Eu32y	[X]Other depressive episodes
Eu32z	[X]Depressive episode, unspecified
Eu33.	[X]Recurrent depressive disorder
Eu330	[X]Recurrent depressive disorder, current episode mild
Eu331	[X]Recurrent depressive disorder, current episode moderate
Eu332	[X]Recurrent depressive disorder, current episode severe without psychotic symptoms
Eu334	[X]Recurrent depressive disorder, currently in remission
Eu33y	[X]Other recurrent depressive disorders
Eu33z	[X]Recurrent depressive disorder, unspecified
Eu341	[X]Dysthymia
E118.	Seasonal affective disorder
E135.	Agitated depression
E2B..	Depressive disorder NEC
E2B1.	Chronic depression
E291.	Prolonged depressive reaction
E204.	Neurotic depression reactive type
E2B0.	Postviral depression
E112.	Single major depressive episode
E1120	Single major depressive episode, unspecified
E1121	Single major depressive episode, mild
E1122	Single major depressive episode, moderate
E1123	Single major depressive episode, severe, without psychosis
E1125	Single major depressive episode, partial or unspecied remission
E1126	Single major depressive episode, in full remission
E112z	Single major depressive episode NOS
E113.	Recurrent major depressive episode
E1130	Recurrent major depressive episodes, unspecified
E1131	Recurrent major depressive episodes, mild
E1132	Recurrent major depressive episodes, moderate
E1133	Recurrent major depressive episodes, severe, no psychosis
E1135	Recurrent major depressive episodes, partial/unspecified remission
E1136	Recurrent major depressive episodes, in full remission
E1137	Recurrent depression
E113z	Recurrent major depressive episode NOS

For peer review only - http://bmjopen.bmj.com/site/about/guidelines.xhtml

Read v2 codes for depression symptoms

Read v2 code	Description
1B17.	Depressed
1B1U.	Symptoms of depression
1BQ..	Loss of capacity for enjoyment
1BT..	Depressed mood
1BU..	Loss of hope for the future
2257	O/E – depressed
1BP..	Loss of interest

Read v2 codes for antidepressant prescriptions

Read v2 code	Description
d71..	Amitriptyline hydrochloride
d72..	Butriptyline - discontinued
d73..	Clomipramine hydrochloride
d74..	Desipramine hydrochloride
d75..	Dosulepin Hydrochloride
d76..	Doxepin
d77..	Imipramine hydrochloride
d78..	Iprindole
d79..	Lofepramine
d7a..	Maprotiline hydrochloride
d7b..	Mianserin hydrochloride
d7c..	Nortriptyline
d7d..	Protriptyline hydrochloride
d7e..	Trazadone hydrochloride
d7f..	Trimipramine
d7g..	Viloxazine hydrochloride
d7h..	Amoxapine
d81..	Phenelzine
d83..	Isocarboxazid
d84..	Tranylcypromine
d85..	Moclobemide
d91..	Compound Antidepressants A-Z
da1..	Flupentixol [Antidepressant]
da2..	Tryptophan
da3..	Fluvoxamine Maleate
da4..	Fluoxetine hydrochloride
da5..	Sertraline hydrochloride
da6..	Paroxetine hydrochloride
da7..	Venlafaxine
da9..	Citalopram
daA..	Reboxetine
daB..	Mirtazapine

For peer review only - http://bmjopen.bmj.com/site/about/guidelines.xhtml

daC..	Escitalopram
daD..	Agomelatine
gde..	Duloxetine
d911.	Limbitrol 5 capsules - discontinued
d912.	Limbitrol 10 capsules - discontinued
d913.	Motipress tablets
x28CP	Discontinued
d914.	Motival tablets discontinued
d916.	Triptafen tablets - only one not discontinued?
d917.	Triftafen-M tablets - discontinued
d8…	Monoamine-oxidase
d82..	Iproniazid

Read v2 codes for anxiety diagnosis

Read v2 code	Description
Eu41.	[X]Other anxiety disorders
Eu410	[X]Panic disorder [episodic paroxysmal anxiety]
Eu411	[X]Generalized anxiety disorder
Eu413	[X]Other mixed anxiety disorders
Eu41y	[X]Other specified anxiety disorders
Eu41z	[X]Anxiety disorder, unspecified
E200.	Anxiety states
E2000	Anxiety state unspecified
E2001	Panic disorder
E2002	Generalised anxiety disorder
E2004	Chronic anxiety
E2005	Recurrent anxiety
E200z	Anxiety state NOS
E202	Phobic disorders
Eu40.	Phobic anxiety disorder
Eu930	[X]Separation anxiety disorder of childhood *
Eu931	[X]Phobic anxiety disorder of childhood*
Eu932	[X]Social anxiety disorder of childhood*
E2D0.	Disturbance of anxiety and fearfulness in childhood and adolescence*
E2D00	Childhood and adolescent overanxiousness disturbance*
E2D0z	Disturbance of anxiety and fearfulness in childhood and adolescence NOS*

Read v2 codes for anxiety symptoms

Read v2 code	Description
1B13.	Anxiousness
2258	O/E - anxious
1B12.	Nerves, nervousness
R2y2.	(D) nervousness

For peer review only - http://bmjopen.bmj.com/site/about/guidelines.xhtml

2259	O/E nervous
225J.	O/E panic attack
1B1V.	C/O panic attack

Read v2 codes for anxiety prescriptions – hypnotics

Read v2 code	Description
d11..	Chloral hydrate
d12..	Clomethiazole edisylate (hypnotic)
d13..	Dichloralphenazone - discontinued
d14..	Flumtrazepam - discontinued
d15..	Flurazepam
d16..	Loprazolam
d17..	Lormetazepam
d18..	Nitrazepam
d1a..	Temazepam (hynotic)
d1b..	Triazolam - discontinued
d1c..	Triclofos sodium
d1d..	Zopiclone
d1f..	Zolpidem
d1g..	Zaleplon
d1h..	Melatonin
d1i..	Dexmedetomidine

Read v2 codes for anxiety prescriptions – hypnotics

Read v2 code	Description
d21..	Diazepam
d22..	Alprazolam
d23..	Bromazepam
d24..	Chlordiazepoxide
d25..	Chlormezanone
d26..	Clobazam
d27..	Clorazepate dipotassium
d28..	Hydroxyzine hcl (anxiolytic)
d29..	Ketazolam - discontinued
d2a..	Lorazepam (anxiolytic)
d2b..	Medazepam - discontinued
d2c..	Meprobamate
d2d..	Oxazepam
d2e..	Prazepam - discontinued
d2f..	Buspirone hydrocholoride
d2g..	Flumazenil

For peer review only - http://bmjopen.bmj.com/site/about/guidelines.xhtml

Read v2 codes for mixed anxiety and depression diagnosis

Read v2 code	Description
E2003	Anxiety with depression
Eu412	[X]Mixed anxiety and depressive disorder

ICD-10 diagnosis codes for depression

ICD-10 code	Description
F321	Moderate depressive episode
F322	Severe depressive episode without psychotic symptoms
F328	Other depressive episodes
F329	Depressive episode, unspecified
F33	Recurrent depressive disorder
F330	Recurrent depressive disorder, current episode mild
F331	Recurrent depressive disorder, current episode moderate
F332	Recurrent depressive disorder, current episode severe without psychotic symptoms
F334	Recurrent depressive disorder, currently in remission
F338	Other recurrent depressive disorders
F339	Recurrent depressive disorder, unspecified
F341	Dysthymia

ICD-10 diagnosis codes for anxiety and mixed anxiety and depression

ICD-10 code	Description
F40	Phobic anxiety disorders
F400	Agoraphobia
F401	Social phobias
F402	Specific (isolated) phobias
F408	Other phobic anxiety disorders
F41	Other anxiety disorders
F410	Panic disorder (episodic paroxysmal anxiety)
F411	Generalized anxiety disorder
F413	Other mixed anxiety disorders
F418	Other specified anxiety disorders
F419	Anxiety disorder, unspecified
F930	Separation anxiety disorder of childhood
F931	Phobic anxiety disorder of childhood
F932	Social anxiety disorder of childhood
F412	Mixed anxiety and depressive disorder

For peer review only - http://bmjopen.bmj.com/site/about/guidelines.xhtml

The RECORD statement – checklist of items, extended from the STROBE statement, that should be reported in observational studies using routinely collected health data.

	Item No.	STROBE items	Location in manuscript where items are reported	RECORD items	Location in manuscript where items are reported
**Title and abstract**					
	1	(a) Indicate the study's design with a commonly used term in the title or the abstract (b) Provide in the abstract an informative and balanced summary of what was done and what was found	(a) The study design is indicated as a data linkage study in the title (b) We have provided an informative and balanced summary in the abstract	RECORD 1.1: The type of data used should be specified in the title or abstract. When possible, the name of the databases used should be included.<br><br>RECORD 1.2: If applicable, the geographic region and timeframe within which the study took place should be reported in the title or abstract.<br><br>RECORD 1.3: If linkage between databases was conducted for the study, this should be clearly stated in the title or abstract.	1.1 The type of data used is specified in the abstract. It was not possible to name all of the databases used in the abstract.<br>1.2 We have reported the geographic region within which the study took place in the title and the abstract, and the timeframe in the abstract<br>1.3 It is clearly stated in the title and the abstract that linkage between databases was conducted for the study
**Introduction**					
Background rationale	2	Explain the scientific background and rationale	The scientific background and rationale for the		

For peer review only - http://bmjopen.bmj.com/site/about/guidelines.xhtml

		for the investigation being reported	investigation is explained in the introduction		
Objectives	3	State specific objectives, including any prespecified hypotheses	The objectives are stated in the introduction		
**Methods**					
Study Design	4	Present key elements of study design early in the paper	The study design is reported at the beginning of the methods section, under the subheading “study design and data source”		
Setting	5	Describe the setting, locations, and relevant dates, including periods of recruitment, exposure, follow-up, and data collection	The setting, locations, and relevant dates are described in the “study population and setting” section		
Participants	6	*(a) Cohort study* - Give the eligibility criteria, and the sources and methods of selection of participants. Describe methods of follow-up<br>*Case-control study* - Give the eligibility criteria, and the sources and methods of case ascertainment and control selection. Give the rationale for the choice of cases and controls	(a) Eligibility criteria, and the sources and methods of selection and follow-up of participants are given in the “study population and setting” section<br>(b) N/A this was not a matched study	RECORD 6.1: The methods of study population selection (such as codes or algorithms used to identify subjects) should be listed in detail. If this is not possible, an explanation should be provided.<br><br>RECORD 6.2: Any validation studies of the codes or algorithms used to select the population should be referenced. If validation was conducted for this study and not	6.1 We have described in detail the methods of study population selection in the “study population and setting” section and the “measures” section<br><br>6.2 We have referenced the validation studies of the anxiety and	

For peer review only - http://bmjopen.bmj.com/site/about/guidelines.xhtml

		Cross-sectional study - Give the eligibility criteria, and the sources and methods of selection of participants<br><br>(b) *Cohort study* - For matched studies, give matching criteria and number of exposed and unexposed<br>*Case-control study* - For matched studies, give matching criteria and the number of controls per case		published elsewhere, detailed methods and results should be provided.<br><br>RECORD 6.3: If the study involved linkage of databases, consider use of a flow diagram or other graphical display to demonstrate the data linkage process, including the number of individuals with linked data at each stage.	depression codes we have used in our study in the "measures" section<br><br>6.3 We have provided a flow diagram with the number of individuals included in each cohort at each stage of linkage (Figure 1)
Variables	7	Clearly define all outcomes, exposures, predictors, potential confounders, and effect modifiers. Give diagnostic criteria, if applicable.	We have clearly defined all relevant measures in the "measures" section	RECORD 7.1: A complete list of codes and algorithms used to classify exposures, outcomes, confounders, and effect modifiers should be provided. If these cannot be reported, an explanation should be provided.	7.1 We have provided a list of codes used in the study in the appendix
Data sources/ measurement	8	For each variable of interest, give sources of data and details of methods of assessment (measurement). Describe comparability of assessment methods if there is more than one group	We have described all data sources and measures in the "study design and data sources" section and the "measures" section		

For peer review only - http://bmjopen.bmj.com/site/about/guidelines.xhtml

Bias	9	Describe any efforts to address potential sources of bias	We have explained that we used a conservative approach to identify cohort participants in the "study population and setting" section		
Study size	10	Explain how the study size was arrived at	We have included a flowchart (Figure 1) to describe the creation of each of the study cohorts and explained the process of cohort creation, referred to in the "study population and setting" section		
Quantitative variables	11	Explain how quantitative variables were handled in the analyses. If applicable, describe which groupings were chosen, and why	Handling of quantitative variables is described in the "measures" section		
Statistical methods	12	(a) Describe all statistical methods, including those used to control for confounding<br>(b) Describe any methods used to examine subgroups and interactions<br>(c) Explain how missing data were addressed<br>(d) *Cohort study* - If applicable, explain how	(a) Statistical methods are described in the "statistical analysis" section<br>(b) N/A<br>(c) N/A<br>(d) N/A<br>(e) Sensitivity analyses are described in the appendix		

For peer review only - http://bmjopen.bmj.com/site/about/guidelines.xhtml

		loss to follow-up was addressed<br>*Case-control study* - If applicable, explain how matching of cases and controls was addressed<br>*Cross-sectional study* - If applicable, describe analytical methods taking account of sampling strategy<br>(e) Describe any sensitivity analyses			
Data access and cleaning methods		..		RECORD 12.1: Authors should describe the extent to which the investigators had access to the database population used to create the study population.<br><br>RECORD 12.2: Authors should provide information on the data cleaning methods used in the study.	12.1 We described the extent to which the investigators had access to the database population in the "data access and cleaning methods" section<br>12.2 We provided information on the data cleaning methods in the "data access and cleaning methods" section
Linkage		..		RECORD 12.3: State whether the study included person-level, institutional-level, or other data linkage across two or more databases. The methods of	12.3 The methods of linkage and quality evaluation are detailed in the "study design and data

For peer review only - http://bmjopen.bmj.com/site/about/guidelines.xhtml

				linkage and methods of linkage quality evaluation should be provided.	source" section and the "study population and setting" section and the flowchart (Figure 1)
**Results**					
Participants	13	(a) Report the numbers of individuals at each stage of the study (*e.g.*, numbers potentially eligible, examined for eligibility, confirmed eligible, included in the study, completing follow-up, and analysed)<br>(b) Give reasons for non-participation at each stage.<br>(c) Consider use of a flow diagram	(a) Numbers of individuals at each study stage are provided in the flow diagrams (Figure 1)<br>(b) Reasons for exclusion are provided on the flowchart (Figure 1)<br>(c) We used a flowchart to illustrate selection of our study cohorts (Figure 1)	RECORD 13.1: Describe in detail the selection of the persons included in the study (*i.e.*, study population selection) including filtering based on data quality, data availability and linkage. The selection of included persons can be described in the text and/or by means of the study flow diagram.	13.1 We described in detail the selection of the persons included in the study in the "study population and setting" section and Figure 1
Descriptive data	14	(a) Give characteristics of study participants (*e.g.*, demographic, clinical, social) and information on exposures and potential confounders<br>(b) Indicate the number of participants with missing data for each variable of interest	(a) Characteristics of study participants are given in Table 2<br>(b) N/A<br>(c) N/A		

For peer review only - http://bmjopen.bmj.com/site/about/guidelines.xhtml

		(c) *Cohort study* - summarise follow-up time (*e.g.*, average and total amount)			
Outcome data	15	*Cohort study* - Report numbers of outcome events or summary measures over time<br>*Case-control study* - Report numbers in each exposure category, or summary measures of exposure<br>*Cross-sectional study* - Report numbers of outcome events or summary measures	Numbers of outcome events are reported in the results section in Table 4		
Main results	16	(a) Give unadjusted estimates and, if applicable, confounder-adjusted estimates and their precision (e.g., 95% confidence interval). Make clear which confounders were adjusted for and why they were included<br>(b) Report category boundaries when continuous variables were categorized	(a) We report unadjusted and adjusted estimates in the results section, and have explained why we adjusted for each factor<br>(b) N/A<br>(c) N/A		

For peer review only - http://bmjopen.bmj.com/site/about/guidelines.xhtml

		(c) If relevant, consider translating estimates of relative risk into absolute risk for a meaningful time period			
Other analyses	17	Report other analyses done—e.g., analyses of subgroups and interactions, and sensitivity analyses	We report on sensitivity analyses in the appendix		
**Discussion**					
Key results	18	Summarise key results with reference to study objectives	Key results are summarised at the beginning of the discussion		
Limitations	19	Discuss limitations of the study, taking into account sources of potential bias or imprecision. Discuss both direction and magnitude of any potential bias	Limitations are discussed in the 'Strengths and limitations' section including potential underestimation of our cohorts	RECORD 19.1: Discuss the implications of using data that were not created or collected to answer the specific research question(s). Include discussion of misclassification bias, unmeasured confounding, missing data, and changing eligibility over time, as they pertain to the study being reported.	We discussed the limitations of the Shielded Patient List in the 'Strengths and Limitations' section
Interpretation	20	Give a cautious overall interpretation of results considering objectives, limitations, multiplicity of analyses, results from	We have given a cautious and balanced interpretation of the results with reference to the previous literature in the discussion		

For peer review only - http://bmjopen.bmj.com/site/about/guidelines.xhtml

		similar studies, and other relevant evidence			
Generalisability	21	Discuss the generalisability (external validity) of the study results	Discussed in the "strengths and limitations" section		
**Other Information**					
Funding	22	Give the source of funding and the role of the funders for the present study and, if applicable, for the original study on which the present article is based	We have provided the funding source, and the role of the funders in the "Role of the funders" section		
Accessibility of protocol, raw data, and programming code		..		RECORD 22.1: Authors should provide information on how to access any supplemental information such as the study protocol, raw data, or programming code.	We have provided information on how to access data within the SAIL databank

*Reference: Benchimol EI, Smeeth L, Guttmann A, Harron K, Moher D, Petersen I, Sørensen HT, von Elm E, Langan SM, the RECORD Working Committee. The REporting of studies Conducted using Observational Routinely-collected health Data (RECORD) Statement. *PLoS Medicine* 2015; in press.

*Checklist is protected under Creative Commons Attribution (CC BY) license.

For peer review only - http://bmjopen.bmj.com/site/about/guidelines.xhtml

Demographic characteristics of the 2019 study population

		General population 2019	CEV children 2019	Chi² P value
N		438,924	599	
**Sex (%)**	Male	224,754 (51.2)	347 (57.9)	<0.001
	Female	214,170 (48.8)	252 (42.1)	
**Age group (%)**	2–7	162,730 (37.1)	231 (38.6)	0.412
	8–12	143,844 (32.8)	181 (30.2)	
	13–17	132,350 (30.2)	187 (31.2)	
**Deprivation quintile (WIMD 2019) (%)**	1 (most deprived)	111,381 (25.4)	134 (22.4)	0.003
	2	93,046 (21.2)	137 (22.9)	
	3	77,600 (17.7)	88 (14.7)	
	4	74,288 (16.9)	94 (15.7)	
	5 (least deprived)	82,609 (18.8)	146 (24.4)	
**Rural/Urban area (%)**	Rural	118,418 (27.0)	165 (27.5)	0.790
	Urban	320,506 (73.0)	434 (72.5)	
**Any history of anxiety or depression**	NO	421,659 (96.1)	565 (94.3)	0.037
	YES	17,265 (3.9)	34 (5.7)	

For peer review only - http://bmjopen.bmj.com/site/about/guidelines.xhtml

Multivariable analysis of risk factors for having a record of anxiety or depression during the COVID-19 pandemic reported using hazard ratios and 95% confidence intervals (model adjusting for demographic factors only)

		Hazard ratio	95% confidence interval	P value
**Cohort**	General population	Reference group		
	Clinically extremely vulnerable (CEV) children	2·81	2.40–3.29	<·001
	Children living with a CEV person	1.09	0·97–1·22	0·14
**Sex**	Male	Reference group		
	Female	1·94	1·84–2.04	<·001
**Age group**	2–7	Reference group		
	8–12	5.21	4·58–5·92	<·001
	13–17	19·39	17.20–21.86	<·001
**Deprivation quintile (WIMD 2019)**	1 (most deprived)	1·13	0·89–1·05	<0.01
	2	1.05	0·95–0.97	0·19
	3	1·05	0·95–0.97	0·23
	4	1·10	0·91–1·01	<0.05
	5 (least deprived)	Reference group		
**Rural/Urban area**	Urban	Reference group		
	Rural	1·02	0·98–0.97	0·45

For peer review only

Multivariable analysis of risk factors for having a record of anxiety or depression during the COVID-19 pandemic reported using hazard ratios and 95% confidence intervals (model adjusting for both demographic and mental health factors)

		Hazard ratio	95% confidence interval	P value
**Cohort**	General population	Reference group		
	Clinically extremely vulnerable (CEV) children	2·27	1·94–2·66	<·001
	Children living with a CEV person	1·02	0·91–1·14	0·746
**Sex**	Male	Reference group		
	Female	1·58	1·50–1·66	<·001
**Age group**	2–7	Reference group		
	8–12	4·56	4·01–5·18	<·001
	13–17	11·05	9·78–12·49	<·001
**Deprivation quintile (WIMD 2019)**	1 (most deprived)	1·02	0·95–1·10	0·565
	2	0·98	0·90–1·06	0·614
	3	1·00	0·92–1·09	0·965
	4	1·07	0·98–1·16	0·127
	5 (least deprived)	Reference group		
**Rural/Urban area**	Urban	Reference group		
	Rural	1·02	0·96–1·02	0·464
**History of anxiety or depression**	No history	Reference group		
	Past history only	5·13	4·75–5·53	<·001
	Recent history only	8·75	8·12–9·44	<·001
	Both recent and past history	18·98	17·52–20·55	<·001

For peer review only

Multivariable analysis of risk factors for having a record of anxiety or depression during the follow-up period March 23rd 2019–January 31st 2020 reported using hazard ratios and 95% confidence intervals (model adjusting for demographic factors only)

		Hazard ratio	95% confidence interval	P value
**Cohort**	General population	Reference group		
	Clinically extremely vulnerable (CEV) children	2·03	1.37–3.01	<·001
**Sex**	Male	Reference group		
	Female	1·85	1·77–1·93	<·001
**Age group**	2–7	Reference group		
	8–12	4·60	4.17–5.08	<·001
	13–17	18·50	16.89–20.27	<·001
**Deprivation quintile (WIMD 2019)**	1 (most deprived)	1·32	1.25–1·41	<·001
	2	1.22	1.15–1·31	<·001
	3	1·13	1.06–1·22	<·001
	4	1·09	1.02–1·17	<0.05
	5 (least deprived)	Reference group		
**Rural/Urban area**	Urban	Reference group		
	Rural	0.97	0·93–1·02	0·21

For peer review only

Multivariable analysis of risk factors for having a record of anxiety or depression during the follow-up period March 23rd 2019–January 31st 2020 reported using hazard ratios and 95% confidence intervals (model adjusting for both demographic and mental health factors)

For peer review only

		Hazard ratio	95% confidence interval	P value
**Cohort**	General population	Reference group		
	Clinically extremely vulnerable (CEV) children	2·03	1.37–3.01	<·001
**Sex**	Male	Reference group		
	Female	1·54	1·48–1·61	<·001
**Age group**	2–7	Reference group		
	8–12	4·13	3.74–4.56	<·001
	13–17	11·56	10.53–12.68	<·001
**Deprivation quintile (WIMD 2019)**	1 (most deprived)	1·23	1.15–1·30	<·001
	2	1.14	1.07–1·22	<·001
	3	1·09	1.02–1·16	<·05
	4	1·05	0·98–1·13	0·17
	5 (least deprived)	Reference group		
**Rural/Urban area**	Urban	Reference group		
	Rural	0.97	0·92–1·02	0·19
**History of anxiety or depression**	No history	Reference group		
	Past history only	4.50	4·21–4.81	<·001
	Recent history only	8·44	7.94–8.96	<·001
	Both recent and past history	16.97	15.85–18.18	<·001

For peer review only - http://bmjopen.bmj.com/site/about/guidelines.xhtml

The RECORD statement – checklist of items, extended from the STROBE statement, that should be reported in observational studies using routinely collected health data.

	Item No.	STROBE items	Location in manuscript where items are reported	RECORD items	Location in manuscript where items are reported
**Title and abstract**					
	1	(a) Indicate the study's design with a commonly used term in the title or the abstract (b) Provide in the abstract an informative and balanced summary of what was done and what was found	(a) The study design is indicated as a data linkage study in the title on page 1<br>(b) We have provided an informative and balanced summary in the abstract on page 2	RECORD 1.1: The type of data used should be specified in the title or abstract. When possible, the name of the databases used should be included.<br><br>RECORD 1.2: If applicable, the geographic region and timeframe within which the study took place should be reported in the title or abstract.<br><br>RECORD 1.3: If linkage between databases was conducted for the study, this should be clearly stated in the title or abstract.	1.1 The type of data used is specified in the abstract on page 2. It was not possible to name all of the databases used in the abstract.<br>1.2 We have reported the geographic region within which the study took place in the title on page 1 and the abstract on page 2, and the timeframe in the abstract on page 2<br>1.3 It is clearly stated in the title (page 1) and the abstract (page 2) that linkage between databases was conducted for the study
**Introduction**					
Background rationale	2	Explain the scientific background and rationale	The scientific background and rationale for the investigation is explained		

		for the investigation being reported	in the introduction on pages 4-5		
Objectives	3	State specific objectives, including any prespecified hypotheses	The objectives are stated in the introduction on pages 5-6		
**Methods**					
Study Design	4	Present key elements of study design early in the paper	The study design is reported at the beginning of the methods section, under the subheading "study design and data source" on page 6		
Setting	5	Describe the setting, locations, and relevant dates, including periods of recruitment, exposure, follow-up, and data collection	The setting, locations, and relevant dates are described in the "study population and setting" section on pages 8-9		
Participants	6	*(a) Cohort study* - Give the eligibility criteria, and the sources and methods of selection of participants. Describe methods of follow-up *Case-control study* - Give the eligibility criteria, and the sources and methods of case ascertainment and control selection. Give the rationale for the choice of cases and controls	(a) Eligibility criteria, and the sources and methods of selection and follow-up of participants are given in the "study population and setting" section on pages 8-9<br>(b) N/A this was not a matched study	RECORD 6.1: The methods of study population selection (such as codes or algorithms used to identify subjects) should be listed in detail. If this is not possible, an explanation should be provided.<br><br>RECORD 6.2: Any validation studies of the codes or algorithms used to select the population should be referenced. If validation was conducted for this study and	6.1 We have described in detail the methods of study population selection in the "study population and setting" section on pages 8-9, and the "measures" section on page 10<br><br>6.2 We have referenced the validation studies of the anxiety and depression codes we have used in our study

		Cross-sectional study - Give the eligibility criteria, and the sources and methods of selection of participants<br><br>*(b) Cohort study* - For matched studies, give matching criteria and number of exposed and unexposed<br>*Case-control study* - For matched studies, give matching criteria and the number of controls per case		not published elsewhere, detailed methods and results should be provided.<br><br>RECORD 6.3: If the study involved linkage of databases, consider use of a flow diagram or other graphical display to demonstrate the data linkage process, including the number of individuals with linked data at each stage.	in the "measures" section on page 10<br><br>6.3 We have provided a flow diagram with the number of individuals included in each cohort at each stage of linkage (Figure 1), referred to on page 8
Variables	7	Clearly define all outcomes, exposures, predictors, potential confounders, and effect modifiers. Give diagnostic criteria, if applicable.	We have clearly defined all relevant measures in the "measures" section on page 10	RECORD 7.1: A complete list of codes and algorithms used to classify exposures, outcomes, confounders, and effect modifiers should be provided. If these cannot be reported, an explanation should be provided.	7.1 We have provided a list of codes used in the study in the appendix (pages 10-14 of the appendix)
Data sources/ measurement	8	For each variable of interest, give sources of data and details of methods of assessment (measurement). Describe comparability of assessment methods if	We have described all data sources and measures in the "study design and data sources" section on pages 8-9 and the "measures" section on page10		

For peer review only

		there is more than one group			
Bias	9	Describe any efforts to address potential sources of bias	We have explained that we used a conservative approach to identify cohort participants in the "study population and setting" section on page 8		
Study size	10	Explain how the study size was arrived at	We have included a flowchart (Figure 1) to describe the creation of each of the study cohorts and explained the process of cohort creation, referred to in the "study population and setting" section on page 8		
Quantitative variables	11	Explain how quantitative variables were handled in the analyses. If applicable, describe which groupings were chosen, and why	Handling of quantitative variables is described in the "measures" section on page 10		
Statistical methods	12	(a) Describe all statistical methods, including those used to control for confounding<br>(b) Describe any methods used to examine subgroups and interactions	(a) Statistical methods are described in the "statistical analysis" section on page 11<br>(b) N/A<br>(c) N/A<br>(d) N/A<br>(e) Sensitivity analyses are described in the		

For peer review only

		(c) Explain how missing data were addressed<br>(d) *Cohort study* - If applicable, explain how loss to follow-up was addressed<br>*Case-control study* - If applicable, explain how matching of cases and controls was addressed<br>*Cross-sectional study* - If applicable, describe analytical methods taking account of sampling strategy<br>(e) Describe any sensitivity analyses	appendix (pages 7-9 of the appendix)		
Data access and cleaning methods		..		RECORD 12.1: Authors should describe the extent to which the investigators had access to the database population used to create the study population.<br><br>RECORD 12.2: Authors should provide information on the data cleaning methods used in the study.	12.1 We described the extent to which the investigators had access to the database population in the “data access and cleaning methods” section on page 7<br>12.2 We provided information on the data cleaning methods in the “data access and cleaning methods” section on page 7

For peer review only

Linkage		..		RECORD 12.3: State whether the study included person-level, institutional-level, or other data linkage across two or more databases. The methods of linkage and methods of linkage quality evaluation should be provided.	12.3 The methods of linkage and quality evaluation are detailed in the "study design and data source" section on pages 6-7, the "study population and setting" section on pages 8-9, and the flowchart (Figure 1), referred to on page 8
**Results**					
Participants	13	(a) Report the numbers of individuals at each stage of the study (*e.g.*, numbers potentially eligible, examined for eligibility, confirmed eligible, included in the study, completing follow-up, and analysed)<br>(b) Give reasons for non-participation at each stage.<br>(c) Consider use of a flow diagram	(a) Numbers of individuals at each study stage are provided in the flow diagrams (Figure 1, referred to on page 8)<br>(b) Reasons for exclusion are provided on the flowchart (Figure 1, referred to on page 8)<br>(c) We used a flowchart to illustrate selection of our study cohorts (Figure 1, referred to on page 8)	RECORD 13.1: Describe in detail the selection of the persons included in the study (*i.e.,* study population selection) including filtering based on data quality, data availability and linkage. The selection of included persons can be described in the text and/or by means of the study flow diagram.	13.1 We described in detail the selection of the persons included in the study in the "study population and setting" section on pages 8-9 and Figure 1 referred to on page 8
Descriptive data	14	(a) Give characteristics of study participants (*e.g.*, demographic, clinical, social) and information	(a) Characteristics of study participants are given in Table 2, page 13<br>(b) N/A<br>(c) N/A		

		on exposures and potential confounders<br>(b) Indicate the number of participants with missing data for each variable of interest<br>(c) *Cohort study* - summarise follow-up time (*e.g.*, average and total amount)			
Outcome data	15	*Cohort study* - Report numbers of outcome events or summary measures over time<br>*Case-control study* - Report numbers in each exposure category, or summary measures of exposure<br>*Cross-sectional study* - Report numbers of outcome events or summary measures	Numbers of outcome events are reported in the results section in Table 4, pages 14-15		
Main results	16	(a) Give unadjusted estimates and, if applicable, confounder-adjusted estimates and their precision (e.g., 95% confidence interval). Make clear which confounders were	(a) We report unadjusted and adjusted estimates in the results section on pages 15-18, and have explained why we adjusted for each factor<br>(b) N/A<br>(c) N/A		

For peer review only

		adjusted for and why they were included<br>(b) Report category boundaries when continuous variables were categorized<br>(c) If relevant, consider translating estimates of relative risk into absolute risk for a meaningful time period			
Other analyses	17	Report other analyses done—e.g., analyses of subgroups and interactions, and sensitivity analyses	We report on sensitivity analyses in the appendix (pages 7-9 of the appendix)		
**Discussion**					
Key results	18	Summarise key results with reference to study objectives	Key results are summarised at the beginning of the discussion on page 21		
Limitations	19	Discuss limitations of the study, taking into account sources of potential bias or imprecision. Discuss both direction and magnitude of any potential bias	Limitations are discussed in the 'Strengths and limitations' section on page 23 including potential underestimation of our cohorts	RECORD 19.1: Discuss the implications of using data that were not created or collected to answer the specific research question(s). Include discussion of misclassification bias, unmeasured confounding, missing data, and changing eligibility over time, as they pertain to the study being reported.	We discussed the limitations of the Shielded Patient List in the 'Strengths and Limitations' section on page 23

For peer review only

Interpretation	20	Give a cautious overall interpretation of results considering objectives, limitations, multiplicity of analyses, results from similar studies, and other relevant evidence	We have given a cautious and balanced interpretation of the results with reference to the previous literature in the discussion on pages 21-22		
Generalisability	21	Discuss the generalisability (external validity) of the study results	Discussed in the "strengths and limitations" section on page 23		
**Other Information**					
Funding	22	Give the source of funding and the role of the funders for the present study and, if applicable, for the original study on which the present article is based	We have provided the funding source on page 25, and the role of the funders in the "Role of the funders" section on pages 7-8		
Accessibility of protocol, raw data, and programming code		..		RECORD 22.1: Authors should provide information on how to access any supplemental information such as the study protocol, raw data, or programming code.	We have provided information on how to access data within the SAIL databank on pages 26-27

*Reference: Benchimol EI, Smeeth L, Guttmann A, Harron K, Moher D, Petersen I, Sørensen HT, von Elm E, Langan SM, the RECORD Working Committee. The REporting of studies Conducted using Observational Routinely-collected health Data (RECORD) Statement. *PLoS Medicine* 2015; in press.

*Checklist is protected under Creative Commons Attribution (CC BY) license.

Effects of the COVID-19 pandemic on the mental health of clinically extremely vulnerable children and children living with clinically extremely vulnerable people in Wales: A data linkage study

Journal:	*BMJ Open*
Manuscript ID	bmjopen-2022-067882.R1
Article Type:	Original research
Date Submitted by the Author:	19-Apr-2023
Complete List of Authors:	Cowley, Laura; Swansea University, Population Data Science<br>Hodgson, Karen; Public Health Wales, Research and Evaluation Division<br>Song, Jiao; Public Health Wales, Health Protection Division<br>Whiffen, Tony; Welsh Government, Administrative Data Research Unit<br>Tan, Jacinta; University of Oxford, Department of Psychiatry<br>John, Ann; Swansea University, Population Data Science<br>Bandyopadhyay, Amrita; Swansea University, National Centre for Population Health and Wellbeing Research<br>Davies, Alisha; Public Health Wales, Research and Evaluation Division
**Primary Subject Heading**:	Mental health
Secondary Subject Heading:	Public health, Health informatics, Health services research, Paediatrics
Keywords:	COVID-19, MENTAL HEALTH, Child & adolescent psychiatry < PSYCHIATRY, PUBLIC HEALTH, Depression & mood disorders < PSYCHIATRY, Anxiety disorders < PSYCHIATRY

SCHOLARONE™
Manuscripts

I, the Submitting Author has the right to grant and does grant on behalf of all authors of the Work (as defined in the below author licence), an exclusive licence and/or a non-exclusive licence for contributions from authors who are: i) UK Crown employees; ii) where BMJ has agreed a CC-BY licence shall apply, and/or iii) in accordance with the terms applicable for US Federal Government officers or employees acting as part of their official duties; on a worldwide, perpetual, irrevocable, royalty-free basis to BMJ Publishing Group Ltd ("BMJ") its licensees and where the relevant Journal is co-owned by BMJ to the co-owners of the Journal, to publish the Work in this journal and any other BMJ products and to exploit all rights, as set out in our licence.

The Submitting Author accepts and understands that any supply made under these terms is made by BMJ to the Submitting Author unless you are acting as an employee on behalf of your employer or a postgraduate student of an affiliated institution which is paying any applicable article publishing charge ("APC") for Open Access articles. Where the Submitting Author wishes to make the Work available on an Open Access basis (and intends to pay the relevant APC), the terms of reuse of such Open Access shall be governed by a Creative Commons licence – details of these licences and which Creative Commons licence will apply to this Work are set out in our licence referred to above.

Other than as permitted in any relevant BMJ Author's Self Archiving Policies, I confirm this Work has not been accepted for publication elsewhere, is not being considered for publication elsewhere and does not duplicate material already published. I confirm all authors consent to publication of this Work and authorise the granting of this licence.

For peer review only

Effects of the COVID-19 pandemic on the mental health of clinically extremely vulnerable children and children living with clinically extremely vulnerable people in Wales: A data linkage study

Laura E Cowley[1], PhD, Karen Hodgson[2], PhD, Jiao Song[3], PhD, Tony Whiffen[4], MSc, Jacinta Tan[5], PhD, Prof Ann John[1], PhD, Amrita Bandyopadhyay[6], MSc, Prof Alisha R Davies[2,6], PhD

Affiliations:
[1] Population Data Science, Swansea University Medical School, Wales, United Kingdom
[2] Research and Evaluation Division, Public Health Data, Knowledge and Research Directorate, Public Health Wales, Cardiff, Wales, United Kingdom
[3] Communicable Disease Surveillance Centre, Health Protection Division, Public Health Wales, Cardiff, Wales, United Kingdom
[4]Administrative Data Research Unit, Welsh Government, Wales, United Kingdom
[5] Department of Psychiatry, University of Oxford
[6] National Centre for Population Health and Wellbeing Research, Swansea University Medical School, Wales, United Kingdom

Address correspondence to: Dr Laura Cowley, Data Science Building, Singleton Park, Swansea, SA2 8PP, Wales, United Kingdom. E-mail: l.e.cowley@swansea.ac.uk

Author email addresses: l.e.cowley@swansea.ac.uk; Karen.Hodgson@wales.nhs.uk; Jiao.Song@wales.nhs.uk; Anthony.Whiffen@gov.wales; Jacinta.Tan@oxfordhealth.nhs.uk; A.John@swansea.ac.uk; A.Bandyopadhyay@swansea.ac.uk; Alisha.davies@wales.nhs.uk

Word count: 3,907

For peer review only - http://bmjopen.bmj.com/site/about/guidelines.xhtml

Abstract

Objectives: To determine whether clinically extremely vulnerable (CEV) children or children living with a CEV person in Wales were at greater risk of presenting with anxiety or depression in primary or secondary care during the COVID-19 pandemic compared with children in the general population, and to compare patterns of anxiety and depression during the pandemic (23rd March 2020–31st January 2021, referred to as 2020/21) and before the pandemic (March 23rd 2019–January 31st 2020, referred to as 2019/20), between CEV children and the general population.

Design: Population-based cross-sectional cohort study using anonymised, linked, routinely collected health and administrative data held in the Secure Anonymised Information Linkage Databank. CEV individuals were identified using the COVID-19 Shielded Patient List.

Setting: Primary and secondary healthcare settings covering 80% of the population of Wales.

Participants: Children aged 2–17 in Wales: CEV (3,769); living with a CEV person (20,033); or neither (415,009).

Primary outcome measure: First record of anxiety or depression in primary or secondary healthcare in 2019/20 and 2020/21, identified using Read and ICD-10 codes.

Results: A Cox regression model adjusted for demographics and history of anxiety or depression revealed that only CEV children were at greater risk of presenting with anxiety or depression during the pandemic compared with the general population (Hazard Ratio=2.27, 95% Confidence Interval=1.94–2.66, p<0.001). Compared with the general population, the risk amongst CEV children was higher in 2020/21 (Risk Ratio 3.04) compared with 2019/20 (Risk Ratio 1.90). In 2020/21, the period prevalence of anxiety or depression increased slightly amongst CEV children, but declined amongst the general population.

Conclusions: Differences in the period prevalence of recorded anxiety or depression in healthcare between CEV children and the general population were largely driven by a reduction in presentations to healthcare services by children in the general population during the pandemic.

For peer review only

Strengths and limitations of this study

- Strengths of this study include its novelty, national focus and clinical relevance; to date this is the first population-based study examining the effects of the COVID-19 pandemic on healthcare use for anxiety or depression amongst clinically extremely vulnerable (CEV) children and children living with a CEV person in Wales
- We compared 2020/21 data with pre-pandemic 2019/20 data for CEV children and children in the general population, to place the impact of the COVID-19 pandemic in the context of longer-term patterns of healthcare use
- We used a novel approach and linked multiple datasets to identify a cohort of children living with a CEV person in Wales during the COVID-19 pandemic
- There was heterogeneity within the Shielded Patient List that was used to create the cohorts of children identified as CEV or living with a CEV person, in terms of the type and severity of individuals' underlying conditions; the manner in which people

For peer review only - http://bmjopen.bmj.com/site/about/guidelines.xhtml

were added to the list; the time point that people were added to the list; and the extent to which people followed the shielding guidance

- Routinely collected healthcare data does not capture self-reported health, and is likely to underestimate the burden of common mental disorders in the population

Keywords: COVID-19, mental health, child and adolescent psychiatry, public health, depression and mood disorders, anxiety disorders

For peer review only

For peer review only - http://bmjopen.bmj.com/site/about/guidelines.xhtml

Introduction

In March 2020, Welsh Government and the Welsh National Health Service sought to protect people deemed clinically extremely vulnerable (CEV) to severe illness or death from COVID-19, advising them to 'shield' at home i.e. to remain indoors and minimise contact with others.[1] To identify CEV people, a Shielded Patient List (SPL) was created, using an algorithm based on clinical code lists and applied centrally to patients' electronic health records.[2] Additionally, health professionals could add people to the list based on their clinical judgement. Shielding was in place from 23rd March 2020 until 16th August 2020 and re-introduced from 22nd December 2020 until 1st April 2021.[1] CEV children were encouraged to return to school at the end of August 2020 taking into account the low rate of severe disease and death from COVID-19 amongst children, balanced against the harms of a lack of schooling and socialization.[3]

Studies have highlighted the detrimental impact of the COVID-19 pandemic on the mental health of CEV adults, with CEV individuals more likely to report increased depressive symptoms and anxiety[4] and to have a clinical record of anxiety or depression during the pandemic compared with those who were not CEV.[1] Meanwhile, studies have reported decreased diagnoses of mental health conditions in primary care across the population as a whole during the pandemic[5,6] (attributed to a reluctance to seek healthcare, or reduced access to services, rather than a decrease in need). However, there is limited evidence on how children accessed healthcare during the pandemic for their mental health, and no studies focusing on CEV children, or children living with a CEV person.

Children are particularly vulnerable to indirect impacts of the pandemic.[7] Drawing on evidence from longitudinal surveys,[8–10] the Department for Education concluded that

For peer review only - http://bmjopen.bmj.com/site/about/guidelines.xhtml

children's mental health declined during the pandemic, reporting that rates of probable mental health disorders were higher from 2020 than before.[11] The data also highlighted variation in mental health trajectories; children with long-term health conditions were more likely to experience poor mental health during the pandemic.[11] However, the extent to which these trends are attributable to the pandemic, or are a continuation of pre-existing upward trends is unclear.

Almost 5,000 CEV children were living in Wales by July 2020; approximately 3.9% of the Welsh CEV population.[12] Children with chronic illnesses are at increased risk of behavioural and emotional problems and psychiatric disorders compared with their peers,[13] but CEV children may be particularly susceptible to mental health difficulties relative to non-CEV children since the pandemic, due to additional restrictions imposed by shielding guidance, potentially exacerbating loneliness and isolation.[14] CEV children may have also experienced heightened health anxiety due to their potential higher risk of severe illness from COVID-19. Additionally, there were almost 14,400 school-aged children living with a CEV person in June 2020[15] who may be at greater risk of mental health difficulties due to both restrictions to protect the vulnerable members of their household, and fears of causing harm.[16]

We investigated the impact of the COVID-19 pandemic on use of healthcare services for anxiety or depression in Wales, for CEV children, children living with a CEV person, and children in the general population, using routinely collected population-level linked data. The primary aim was to determine whether CEV children or children living with a CEV person were more likely to have a record for anxiety or depression in primary or secondary care during the pandemic compared with children in the general population. The secondary aim was to compare patterns of anxiety or depression in 2019/2020 and 2020/2021 between CEV

For peer review only - http://bmjopen.bmj.com/site/about/guidelines.xhtml

children and the general population, to place the impact of COVID-19 and the shielding guidance in the context of longer-term patterns of healthcare use.

Methods

Study design and data source

This is a population-based cross-sectional cohort study using anonymised, linked, routinely collected health and administrative data for the population of Wales, UK, held in the Secure Anonymised Information Linkage (SAIL) databank (www.saildatabank.com). Within the SAIL databank, encrypted linkage fields are used to link data anonymously from various sources at individual and household level (appendix pp 1–2); known as Anonymised Linking Fields (ALFs) for individuals and Residential Anonymised Linking Fields (RALFs) for residences.[17,18] We used these to link multiple datasets in this study (Table 1). General Practices (GPs) opt-in to providing data to SAIL; currently, SAIL contains primary care data for around 80% of the Welsh population, and the available data are representative of the entire Welsh population with respect to age, sex, and deprivation.[19] The SAIL databank was interrogated using DB2 structured query language (SQL).

Table 1. Datasets used in this study

Dataset	Description
Welsh Demographic Service (WDS) dataset	A register containing demographic information about all individuals registered at a Welsh General Practice (GP)
COVID-19 Shielded People List (CVSP)	A dataset containing information about clinically extremely vulnerable individuals in Wales, including reasons for shielding
Welsh Index of Multiple Deprivation 2019 (WIMD 2019)	A dataset containing deprivation scores corresponding to all Lower Super Output Areas (LSOAs; geographic units comprised of around 1,600 individuals) in Wales[24]
Rural Urban Classification (RUC) dataset	A dataset containing information on urban/rural categories corresponding to all LSOAs in Wales
Annual District Death Extract (ADDE)	A register containing details of all deaths of Welsh residents, including information regarding date and cause of death

For peer review only - http://bmjopen.bmj.com/site/about/guidelines.xhtml

Outpatient Dataset (OPD)	A dataset containing attendance information for all hospital outpatient appointments in Wales
National Community Child Health Database (NCCHD)	A register of children born in Wales, containing data collected at birth, including a maternal ALF to link children with their biological mothers
Patient Episode Database for Wales (PEDW)	A dataset containing attendance and clinical information for all hospital admissions in Wales, including data regarding diagnoses
Care Homes Data (CHD)	A dataset containing residential information about adult care homes in Wales
Welsh Longitudinal General Practice (WLGP) dataset	A dataset containing attendance and clinical information for all GP interactions including symptoms, diagnoses, and prescriptions

Ethical Approval

This study used anonymised data and therefore did not require National Research Ethics Committee approval. Approval to access and link the data within SAIL was granted by the Information Governance Review Panel under project number 1265 (appendix p 3).

Patient and Public Involvement

The study protocol was presented at the SAIL consumer panel meeting prior to study commencement. This panel consists of members of the public with an interest in data and its uses to improve services and healthcare. The panel provided advice and feedback on the study design from a public perspective.

Data Access and Cleaning Methods

All authors had full access to all the data in the study. Data cleaning included deduplication and restructuring of the SPL prior to cohort creation and analysis, and was undertaken by LEC.

Role of the funding source

For peer review only - http://bmjopen.bmj.com/site/about/guidelines.xhtml

For peer review only

The funders had no role in the study design, data collection, data analysis, interpretation, writing of the report, or the decision to submit the paper for publication.

Study population and setting

We created three study cohorts for 2020: 1) CEV children 2) children living with at least one CEV person and 3) a general population group of children who were *not* identified as CEV or living with a CEV person, along with two further cohorts for 2019 for comparison purposes. Children who were both CEV and living with a CEV person were categorised as CEV. Figure 1 shows a flow diagram of the inclusion criteria for each cohort. We included all children aged 2–17 years who were alive, living in Wales and registered with a SAIL-supplying GP on 23rd March 2020; and who had either an exact match on NHS number or demographics (name, date of birth, gender code, and postcode), or a probabilistic match of 90% or greater.[17] We excluded those for whom full demographic or residence data were not available.

Cohort 1: Clinically extremely vulnerable children

Cohort 1 consisted of all children who were identified as CEV (either by algorithm[2] or health professionals) between 23rd March 2020 and 16th August 2020 (N=3,769).

Cohort 2: Children living with at least one clinically extremely vulnerable person

To identify children living with a CEV person, we first identified the RALF for all CEV people in Wales as of 23rd March 2020, including dates of residence. To minimize bias, we then adopted a conservative approach and included 1) children who were recorded as residents at the same RALF on 23rd March 2020 and had an entry date of residence within 6 months of the entry date of the CEV person 2) children born to mothers who were recorded as resident at the same RALF as the CEV person, or 3) children who shared the same

For peer review only - http://bmjopen.bmj.com/site/about/guidelines.xhtml

maternal ALF as a younger CEV child. We excluded 1) adult care homes as these were unlikely to contain children[20] and 2) RALFs containing more than ten people, as the Unique Property Reference Number (UPRN) from which the RALF is derived, is considered inaccurate in this case[21], with a final 20,033 children in cohort 2.

Cohort 3: Children in the general population

Cohort 3 consisted of all other children who were alive, living in Wales and registered with a SAIL-supplying GP on 23rd March 2020 (N=415,009).

Clinically extremely vulnerable children in Wales in 2019 (pre-COVID-19 CEV children)

To explore longer-term patterns of anxiety or depression amongst children with the conditions included within the shielding guidance, we created a cohort of pre-COVID-19 "CEV" children who had similar health concerns to CEV children in a period prior to 2020. Creation of this pre-pandemic cohort enabled us to explore whether adverse mental health outcomes for CEV children during the pandemic were likely attributable to the shielding guidance, or whether CEV children experience poorer mental health compared with the general population regardless of having to shield. We included children who were alive, living in Wales, and registered with a SAIL-supplying GP one year prior to the introduction of shielding (23rd March 2019) and who had one or more of three of the health condition categories warranting inclusion on the SPL (respiratory illnesses, blood or bone cancer, and immunosuppression therapy). These categories were chosen as they only required the patient to have one of the listed codes within a given time period and therefore children with these conditions could be identified with a high degree of certainty. We used International Classification of Diseases (ICD) version 10 diagnostic codes and OPCS Classification of Interventions and Procedures version 4 codes for these categories, which were taken from the

For peer review only - http://bmjopen.bmj.com/site/about/guidelines.xhtml

SPL documentation and provided in the appendix (pp 4–5). For comparison purposes we also created a general population cohort of children aged 2–17 who were alive, living in Wales, and registered with a SAIL-supplying GP as of 23rd March 2019. A flow diagram of inclusion criteria for these cohorts is provided in the appendix (p 6). We performed a sensitivity analysis to confirm the validity of this approach (appendix pp 7–9).

Measures

Outcome of interest: Anxiety or depression

The outcome of interest was the first record of anxiety or depression in primary or secondary healthcare data during the COVID-19 pandemic (i.e. 23rd March 2020–31st January 2021, referred to as 2020/21), and pre-pandemic (March 23rd 2019–January 31st 2020, referred to as 2019/20). We included any healthcare visit where anxiety or depression was documented in the electronic health record. We used validated Read v2 codes to identify children with primary care records for anxiety[22] or depression[23] (including diagnoses, symptoms, and prescriptions) in the WLGP dataset. Read codes are a hierarchical terminology system that encode clinical, diagnostic and therapeutic patient information, and enable data entry of patient care information following a primary care consultation. Separate Read codes are used to record a patients' reported past medical history, or to note that a clinician is aware of a past condition. We used ICD-10 diagnostic codes to identify children with hospital admissions or outpatient appointments for anxiety or depression in the PEDW and OPD datasets. If children had multiple records of anxiety or depression during the relevant time periods, we sequenced these and selected the record with the earliest date. Code lists were reviewed by a clinician with expertise in child psychiatry (JT) and are provided in the appendix (pp 10–14).

Covariates: History of anxiety or depression

For peer review only - http://bmjopen.bmj.com/site/about/guidelines.xhtml

We used the same process to identify children in the study population with a 'recent' or 'past' history of anxiety or depression, defined as any record in the year prior to the pandemic (23rd March 2019–23rd March 2020), and any record occurring any time before 23rd March 2019 respectively.[1]

Covariates: Demographics

We calculated age and determined Lower-layer Super Output Areas (LSOA) as of 23rd March 2020. LSOA codes were derived from the WDS dataset based on the child's RALF, and used to ascertain deprivation quintiles and urban/rural classification by linking to the WIMD 2019[24] and RUC datasets.

Statistical analysis

We used R version 4.1.1 for statistical analyses. P-values of <0.05 were considered statistically significant.

Examining risk of anxiety or depression between different populations in 2020/21

We tested the hypothesis that there was no difference in the risk of having a record of anxiety or depression in 2020/21 between the three cohorts (CEV children, children living with a CEV person, and children in the general population). We plotted Kaplan-Meier survival curves for each cohort. We used Cox regression to calculate unadjusted and adjusted hazard ratios (HRs) with 95% confidence intervals (CI). We report three models examining the risk of having a record of anxiety or depression during the pandemic compared with the general population; (1) unadjusted, (2) adjusted for demographic factors (age group, sex, deprivation and rurality) and (3) adjusted for demographic factors and previous history of anxiety or depression (no history, recent history, past history, or both recent and past history).

For peer review only - http://bmjopen.bmj.com/site/about/guidelines.xhtml

Examining risk of anxiety or depression between different populations in 2019/20

We tested the hypothesis that there was no difference in the risk of having a record of anxiety or depression in 2019/20 between two cohorts ("CEV" children and all other children living in Wales in 2019). As above, we calculated unadjusted and adjusted HRs, and calculated the change in the period prevalence of depression and anxiety for these two groups between 2019 and 2020.

Study reporting

This study is reported in accordance with the Reporting of Studies conducted using Observational Routinely-collected data (RECORD) guidelines[25] (appendix pp 15–23).

Results

Descriptive statistics and demographic characteristics of the study population

Demographic characteristics of the 2020/21 study population are presented in Table 2, and for the 2019/20 study population in the appendix (p 24). For both years, there were greater proportions of boys, older children (aged 13–17), children living in the least and most deprived quintiles and children with a history of anxiety or depression in the CEV children than for the general population. In 2020/21, a greater proportion of children living with a CEV person were older (aged 13–17), and had a history of anxiety or depression, compared with the general population (Table 2). The conditions leading to children being identified as CEV in 2020/21 are shown in Table 3.

For peer review only - http://bmjopen.bmj.com/site/about/guidelines.xhtml

Table 2. Characteristics of clinically extremely vulnerable (CEV) children, children living with a CEV person, and a general population group of children neither CEV or living with a CEV person, 2020/21, Wales

		General population	Clinically extremely vulnerable children	χ^2 statistic, degrees of freedom, and p value, general population v. clinically extremely vulnerable children	Children living with a clinically extremely vulnerable person	χ^2 statistic, degrees of freedom, and p value, general population v. children living with a clinically extremely vulnerable person
N		415,009	3,769		20,033	
**Sex (%)**	Male	212,311 (51·2)	2,184 (57·9)	χ^2=68·6, df=1, p<0·001	10,247 (51·2)	χ^2=0·0, df=1, p=0·989
	Female	202,698 (48·8)	1,585 (42·1)		9,786 (48·8)	
**Age group (years) (%)**	2–7	150,999 (36·4)	1,141 (30·3)	χ^2=84·0, df=2, p<0·001	6,692 (33·4)	χ^2=93·7, df=2, p<0·001
	8–12	137,310 (33·1)	1,247 (33·1)		6,678 (33·3)	
	13–17	126,700 (30·5)	1,381 (36·6)		6,663 (33·3)	
**Deprivation quintile (WIMD 2019) (%)**	1	106,075 (25·6)	1,014 (26·9)	χ^2=11·1, df=4, p=0·025	5,058 (25·2)	χ^2=10·8, df=4, p=0·029
	2	87,930 (21·2)	773 (20·5)		4,107 (20·5)	
	3	73,106 (17·6)	635 (16·8)		3,611 (18·0)	
	4	69,789 (16·8)	589 (15·6)		3,488 (17·4)	
	5	78,109 (18·8)	758 (20·1)		3,769 (18·8)	
**Rural/Urban area (%)**	Rural	110,682 (26·7)	971 (25·8)	χ^2=1·5, df=1, p=0·217	5,444 (27·2)	χ^2=2·5, df=1, p=0·116
	Urban	304,327 (73·3)	2,798 (74·2)		14,589 (72·8)	
**Any history of anxiety or depression (%)**	YES	17,986 (4·3)	368 (9·8)	χ^2=261·5, df=1, p<0·001	1,131 (5·6)	χ^2=78·0, df=1, p<0·001
	NO	397,023 (95·7)	3,401 (90·2)		18,902 (94·4)	

For peer review only

Table 3. Proportion of clinically extremely vulnerable children with different conditions contributing to underlying reasons to shield

Reason for shielding	Number and percentage of clinically extremely vulnerable children (n = 3,769)
Rare diseases	1,227 (32·6%)
Organ disease	735 (19·5%)
Respiratory illness	660 (17·5%)
Immunosuppression therapy	491 (13%)
Cancer	147 (3·9%)
Transplant	132 (3·5%)
Renal dialysis	10 (0·3%)
GP referred *(reason unknown)*	582 (15·4%)
Other *(reason unknown)*	22 (0·6%)

In children whose reasons for shielding were known (3,165/3,769) 6.3% (198/3,165) had more than one condition

Risk of anxiety or depression in children in 2020/21

Numbers of censored children in each group (due to death, migration or registration with a non-SAIL supplying GP) and numbers with a record for anxiety or depression are presented in Table 4. Of those with a record, 5,768/6,251 (92.3%) presented to primary care while 483/6,251 (7.7%) presented to secondary care.

Table 4. Number of children who were censored, with no event, or who had a record of anxiety or depression during 2020/21

	General population	Clinically extremely vulnerable children	Children living with a clinically extremely vulnerable person
N	415009	3769	20033
Died (%)	12 (0·003)	6 (0·2)	0 (0·0)
Moved out of Wales (%)	7297 (1·8)	68 (1·8)	245 (1·2)
Moved to a non SAIL-supplying GP (%)	5664 (1·4)	31 (0·8)	262 (1·3)

For peer review only - http://bmjopen.bmj.com/site/about/guidelines.xhtml

No event (%)	396267 (95·5)	3505 (93·0)	19203 (95·9)
Anxiety or depression during the pandemic 2020/21 (%)	5,769 (1·4)	159 (4·2)	323 (1·6)

In the unadjusted model, both CEV children and children living with a CEV person were at significantly greater risk of having a record of anxiety or depression during the pandemic compared with the general population (HR=3·09, 95% CI=2·64–3·61, p<0·001 and HR=1·16, 95% CI=1·04–1·30, p<0·05, respectively). For CEV children, the hazard ratio remained significant when adjusting for demographic factors including age, sex, deprivation and rurality (Table 5), and when adjusting for demographic factors and previous clinical history of anxiety or depression (Table 6). However, for children living with a CEV person, the hazard ratio was no longer significant in either of the adjusted models. The unadjusted survival curves for each cohort are shown in Figure 2.

For peer review only - http://bmjopen.bmj.com/site/about/guidelines.xhtml

Table 5

Multivariable analysis of risk factors for having a record of anxiety or depression during the COVID-19 pandemic (23rd March 2020–31st January 2021), reported using hazard ratios and 95% confidence intervals (model adjusting for demographic factors only)

		Hazard ratio	95% confidence interval	P value
**Cohort**	General population	Reference group		
	Clinically extremely vulnerable (CEV) children	2·81	2.40–3.29	<·001
	Children living with a CEV person	1.09	0·97–1·22	0·14
**Sex**	Male	Reference group		
	Female	1·94	1·84–2.04	<·001
**Age group**	2–7	Reference group		
	8–12	5.21	4·58–5·92	<·001
	13–17	19·39	17.20–21.86	<·001
**Deprivation quintile (WIMD 2019)**	1 (most deprived)	1·13	0·89–1·05	<0.01
	2	1.05	0·95–0.97	0·19
	3	1·05	0·95–0.97	0·23
	4	1·10	0·91–1·01	<0.05
	5 (least deprived)	Reference group		
**Rural/Urban area**	Urban	Reference group		
	Rural	1·02	0·98–0.97	0·45

For peer review only - http://bmjopen.bmj.com/site/about/guidelines.xhtml

Table 6. Multivariable analysis of risk factors for having a record of anxiety or depression during the COVID-19 pandemic (23rd March 2020–31st January 2021), reported using hazard ratios and 95% confidence intervals (model adjusting for both demographic and mental health factors)

		Hazard ratio	95% confidence interval	P value
**Cohort**	General population	Reference group		
	Clinically extremely vulnerable (CEV) children	2·27	1·94–2·66	<·001
	Children living with a CEV person	1·02	0·91–1·14	0·746
**Sex**	Male	Reference group		
	Female	1·58	1·50–1·66	<·001
**Age group**	2–7	Reference group		
	8–12	4·56	4·01–5·18	<·001
	13–17	11·05	9·78–12·49	<·001
**Deprivation quintile (WIMD 2019)**	1 (most deprived)	1·02	0·95–1·10	0·565
	2	0·98	0·90–1·06	0·614
	3	1·00	0·92–1·09	0·965
	4	1·07	0·98–1·16	0·127
	5 (least deprived)	Reference group		
**Rural/Urban area**	Urban	Reference group		
	Rural	1·02	0·96–1·02	0·464
**History of anxiety or depression**	No history	Reference group		
	Past history only	5·13	4·75–5·53	<·001
	Recent history only	8·75	8·12–9·44	<·001
	Both recent and past history	18·98	17·52–20·55	<·001

For peer review only

For peer review only - http://bmjopen.bmj.com/site/about/guidelines.xhtml

Risk of anxiety or depression in children in 2019/2020

In 2019/20, in the unadjusted model there was an increased risk of having a record of anxiety or depression amongst the "CEV" children compared with the general population (HR=1·94, 95% CI=1·31–2·87, p<0·001). This remained evident in the adjusted models (Table 7 and Table 8). The unadjusted survival curves for each cohort are shown in Figure 3.

For peer review only

For peer review only - http://bmjopen.bmj.com/site/about/guidelines.xhtml

Table 7

Multivariable analysis of risk factors for having a record of anxiety or depression between March 23rd 2019–January 31st 2020, reported using hazard ratios and 95% confidence intervals (model adjusting for demographic factors only)

		Hazard ratio	95% confidence interval	P value
**Cohort**	General population	Reference group		
	Clinically extremely vulnerable (CEV) children	2·03	1.37–3.01	<·001
**Sex**	Male	Reference group		
	Female	1·85	1·77–1·93	<·001
**Age group**	2–7	Reference group		
	8–12	4·60	4.17–5.08	<·001
	13–17	18·50	16.89–20.27	<·001
**Deprivation quintile (WIMD 2019)**	1 (most deprived)	1·32	1.25–1·41	<·001
	2	1.22	1.15–1·31	<·001
	3	1·13	1.06–1·22	<·001
	4	1·09	1.02–1·17	<0.05
	5 (least deprived)	Reference group		
**Rural/Urban area**	Urban	Reference group		
	Rural	0.97	0·93–1·02	0·21

For peer review only

Table 8. Multivariable analysis of risk factors for having a record of anxiety or depression between March 23rd 2019–January 31st 2020, reported using hazard ratios and 95% confidence intervals (model adjusting for both demographic and mental health factors)

		Hazard ratio	95% confidence interval	P value
**Cohort**	General population	Reference group		
	Clinically extremely vulnerable (CEV) children	2·03	1.37–3.01	<·001
**Sex**	Male	Reference group		
	Female	1·54	1·48–1·61	<·001
**Age group**	2–7	Reference group		
	8–12	4·13	3.74–4.56	<·001
	13–17	11·56	10.53–12.68	<·001
**Deprivation quintile (WIMD 2019)**	1 (most deprived)	1·23	1.15–1·30	<·001
	2	1.14	1.07–1·22	<·001
	3	1·09	1.02–1·16	<0·05
	4	1·05	0·98–1·13	0·17
	5 (least deprived)	Reference group		
**Rural/Urban area**	Urban	Reference group		
	Rural	0.97	0·92–1·02	0·19
**History of anxiety or depression**	No history	Reference group		
	Past history only	4.50	4·21–4.81	<·001
	Recent history only	8·44	7.94–8.96	<·001
	Both recent and past history	16.97	15.85–18.18	<·001

For peer review only

Difference in the risk of records of anxiety or depression between 2019/20 and 2020/21

In 2019/20, “CEV” children had increased risk of having recorded anxiety or depression compared with children in the general population, and in 2020/21 the risk ratio increased to 3.04 (Table 9). This reflects a marked decline in presentation amongst children in the general population over this period (from 2·19% to 1·39%), alongside a small increase for CEV children (from 4·17% to 4·22%).

For peer review only

For peer review only - http://bmjopen.bmj.com/site/about/guidelines.xhtml

Table 9. Risk of records of anxiety or depression in children (aged 2–17 years) who were clinically extremely vulnerable (CEV) and those in the general population, in 2019/20 and 2020/21

**Time period**	**CEV children**<br>*2020/21 - children identified through shielded patient list*<br>*2019/20 – comparable cohort of children with the conditions listed in the shielded patient list*			**Children in the general population in Wales**			**Crude Risk Ratio**
	**No. children with recorded anxiety or depression**	**Total No. of children**	**Period Prevalence (%)**	**No. children with recorded anxiety or depression**	**Total No. of children**	**Period Prevalence(%)**	
2019/20 (Pre COVID-19)	25	599	4·17	9,620	438,924	2·19	1·90
2020/21 (During COVID-19)	159	3,769	4·22	5,769	415,009	1·39	3·04
**Percentage point change over time points**			-0·05			0·8	

For peer review only - http://bmjopen.bmj.com/site/about/guidelines.xhtml

Discussion

In Wales, CEV children and children living with a CEV person were more likely to access health services for anxiety or depression during the pandemic than children in the general population. For CEV children, this pattern remained evident after adjusting for demographic differences and the likelihood of having a previous history of anxiety or depression. Although a small increase in risk was found for children living with a CEV person, after adjusting for demographic characteristics and previous history of anxiety and depression, this was no longer significant.

Both before and during the pandemic, the groups with the greatest risk of having a record for anxiety or depression were adolescents aged 13–17, and those with both a past and recent history of anxiety or depression. Females were also more likely than males to have a record for anxiety or depression in both time periods. These findings are in line with previous studies reporting worse mental health in female adolescents compared with males[26]; a higher prevalence of anxiety and depression in adolescents and females compared with younger children and males during the pandemic[27]; and worse mental health outcomes during the pandemic for those with pre-existing mental health difficulties[28]. This suggests that moving forward, it will be important to prioritise mental health support for female adolescents, and particularly for children who have concomitant physical and mental health conditions.

Given the detailed methodology used to identify CEV individuals in Wales,[2] we were able to develop a comparable cohort of children with a subset of health conditions before the pandemic, using routine healthcare data. This enabled us to examine patterns of presentation for anxiety and depression amongst CEV children outside of the context of the pandemic. We found this group were at greater risk of having a record for anxiety or depression compared

For peer review only - http://bmjopen.bmj.com/site/about/guidelines.xhtml

with children in the general population in 2019/20, before COVID-19. In 2020/21, CEV children remained at higher risk, and the difference was greater, although this is explained by a marked decline amongst children in the general population presenting to healthcare services with anxiety or depression during this time.

The reduction in presentations for anxiety and depression amongst children in the general population most likely reflects reduced access to NHS services during the pandemic. Other evidence suggests increased demand and unmet need for mental health support in the UK, for children with and without pre-existing mental health problems, since 2020.[29,30] Findings from self-report surveys[8–10] and the current study suggest that the pandemic has widened the gap between need and access to mental healthcare for the general population of children in Wales, but additional data are required to unpack the relationship between self-reported mental health needs and presentation to healthcare services.

Meanwhile, the relatively stable period prevalence of anxiety and depression for CEV children in 2019/20 and 2020/21 could indicate that this group did not experience an increase in mental health needs during the pandemic over and above past years, and that they had access to mental health support through existing care pathways for underlying conditions. Alternatively, if CEV children experienced the same increase in mental health needs as reported elsewhere amongst the general population, then these figures may mask unmet demand for mental health support among this group. A survey of adults supports the latter explanation, reporting increased anxiety and depressive symptoms amongst shielding individuals compared with non-shielding individuals.[4] However, we have found no UK surveys focusing on the mental health of CEV children.

For peer review only - http://bmjopen.bmj.com/site/about/guidelines.xhtml

Our finding that children living with a CEV person were at no greater risk of presenting with anxiety or depression during the pandemic compared with the general population (after controlling for other factors), could be interpreted in two ways. It is possible that the impact of the pandemic on the mental health of children living with a CEV person was not as great as for CEV children, but this seems unlikely given that research has suggested increased anxiety amongst children who were shielding their siblings.[16] Another explanation is that children living with a CEV person suffered from similar barriers to access to mental healthcare services to the general population, and did not have the same routes to access that CEV children did. This explanation is supported by research with shielding families, which suggests that they have felt left behind, and that children living with a CEV person may have 'fallen under the radar of educational and healthcare professionals'.[16]

Strengths and limitations

To our knowledge, this is the first population-based study examining the effects of the COVID-19 pandemic on healthcare use for anxiety or depression amongst CEV children and children living with a CEV person in Wales. Linkage of population-based routinely collected data is a valuable method for generating evidence with a high level of external validity and applicability for policy making. A strength of this study is the comparison of 2020/21 data with pre-pandemic 2019/20 data for CEV children and children in the general population. Another strength is the use of a novel approach using multiple linked datasets to identify a cohort of children living with a CEV person during the pandemic.

This study used the SPL to create cohorts of children identified as CEV and a cohort of children living with a CEV person. There was heterogeneity within the SPL in terms of the type and severity of individuals' underlying conditions; the manner in which people were

For peer review only - http://bmjopen.bmj.com/site/about/guidelines.xhtml

added to the list (via the algorithm or clinical judgement); the time point that people were added to the list; and the extent to which people followed the shielding guidance. In addition, the impact of following shielding guidance is likely to have varied due to individual circumstances and the level of support received. The 2019 "CEV" cohort was a relatively small sample and for pragmatic reasons, only included children with a subset of the conditions included in the shielding guidance. To identify children living with a CEV person, we adopted a conservative approach and included children only if they were living with their mother. We took this approach in order to minimize bias and increase the generalisability of the findings, however this approach is likely to have underestimated the number of children living with a CEV person. Finally, this study focused on healthcare use using clinical codes. Routinely collected healthcare data does not capture self-reported health, and is likely to underestimate the burden of common mental disorders in the population.[31] Focusing on healthcare use with routine data alone cannot tell us about the underlying reasons for changes in utilisation, or the scale of mental health need.

Implications for policy and practice

Our findings have implications for recovery planning to prevent, mitigate and respond to the mental health impacts of the pandemic. We have shown changes in presentation to primary and secondary healthcare services with anxiety and depression for CEV children and children in the general population during the pandemic, and there are concerns regarding potential increases in unmet mental health needs over time. As highlighted by UK organisations such as the Centre for Mental Health,[32] services face challenges in tackling this demand. This has been recognised by Welsh Government, who invested an additional £9.4m in children's mental health services in 2021.[33]

For peer review only - http://bmjopen.bmj.com/site/about/guidelines.xhtml

This novel linked data study contributes to our understanding of the direct and indirect impact of shielding on children's mental health in Wales during the COVID-19 pandemic. This evidence should be considered in light of additional, more detailed routine healthcare linkage studies, and national surveys, to provide a comprehensive understanding of the relationship between mental health support needs, expressed demands and care provision to better target services to those who need them the most.

Beyond the indirect impacts of the pandemic, our findings highlight the increased mental health needs of children with serious medical conditions. Given that these children are likely to have greater contact with healthcare services, signposting across services including mental health services, is likely to be beneficial.

Funding

This work was supported by the Adolescent Mental Health Data Platform (ADP). The ADP is funded by MQ Mental Health Research Charity (Grant Reference MQBF/3 ADP). This work was also supported by the National Centre for Population Health and Wellbeing Research (NCPHWR) which is funded by Health and Care Research Wales.

Acknowledgements

ADP and the authors would like to acknowledge the data providers who supplied the datasets enabling this research study. The views expressed are entirely those of the authors and should not be assumed to be the same as those of ADP or MQ Mental Health Research Charity. This study makes use of anonymised data held in the Secure Anonymised Information Linkage

For peer review only - http://bmjopen.bmj.com/site/about/guidelines.xhtml

(SAIL) Databank. We would like to acknowledge all the data providers who make anonymised data available for research.

Contributors

LEC: study design, literature search, data curation, data analysis, figures, data interpretation, writing – original draft, writing – review and editing; KH: data analysis, data interpretation, supervision, writing – review and editing; JS: conceptualisation, study design, methodology, data curation, data analysis, data interpretation, supervision, writing – review and editing; TW: methodology, writing – review and editing; JT: validation, writing – review and editing; AJ: methodology, funding acquisition, writing – review and editing; AB: methodology, writing – review and editing; ARD: conceptualisation, study design, data analysis, data interpretation, supervision, funding acquisition, writing – review and editing. LEC and JS verified the underlying data.

Competing interests

Ann John is a member of the Welsh Government COVID-19 Technical Advisory Group and is also co-chair of the Scientific Pandemic Insights Group on Behaviours (SPI-B) which is a subgroup of the Scientific Advisory Group for Emergencies (SAGE) advising the UK government. None of the other authors have any competing interests.

Data availability statement

The data used in this study are available in the SAIL Databank at Swansea University (Swansea, UK) via the Adolescent Mental Health Data Platform, but, as restrictions apply, they are not publicly available. All proposals to use SAIL data are subject to review by an independent Information Governance Review Panel (IGRP). Before any data can be

For peer review only - http://bmjopen.bmj.com/site/about/guidelines.xhtml

accessed, approval must be given by the IGRP. The IGRP carefully considers each project to ensure proper and appropriate use of SAIL data. When access has been granted, it is gained through a privacy-protecting safe haven and remote access system referred to as the SAIL Gateway. SAIL have established an application process to be followed by anyone who would like to access data via SAIL, details of which can be found at https://saildatabank.com/data/apply-to-work-with-the-data/

References

1 Davies A, Song J, Bentley L, *et al.* COVID-19 in Wales: The impact on levels of health care use and mental health of the clinically extremely vulnerable. Cardiff: Public Health Wales. 2021. https://phw.nhs.wales/services-and-teams/knowledge-directorate/research-and-evaluation/publications/covid-19-in-wales-the-impact-on-levels-of-health-care-use-and-mental-health-of-the-clinically-extremely-vulnerable/ (accessed March 2, 2022).

2 NHS Wales Informatics Service. COVID-19 high risk shielded patient list identification methodology. 2020. https://nwis.nhs.wales/coronavirus/coronavirus-content/coronavirus-documents/covid-19-high-risk-shielded-patient-list-identification-methodology-v3-2-17th-august/ (accessed Dec 10, 2020).

3 Department of Health and Social Care. Statement from the UK Chief Medical Officers on schools and childcare reopening. 2020. https://www.gov.uk/government/news/statement-from-the-uk-chief-medical-officers-on-schools-and-childcare-reopening (accessed Dec 12, 2020).

4 Di Gessa G, Price D. The impact of shielding during the COVID-19 pandemic on mental health: evidence from the English Longitudinal Study of Ageing. *Br J Psychiatry* 2022; published online April 4. https://doi.org/10.1192/bjp.2022.44.

5 Mansfield KE, Mathur R, Tazare J, *et al.* Indirect acute effects of the COVID-19 pandemic on physical and mental health in the UK: a population-based study. *Lancet Digit Heal* 2021; **3**: e217–30.

6 Carr MJ, Steeg S, Webb RT, *et al.* Effects of the COVID-19 pandemic on primary care-recorded mental illness and self-harm episodes in the UK: a population-based cohort study. *Lancet Public Heal* 2021; **6**: e124–35.

7 Crawley E, Loades M, Feder G, Logan S, Redwood S, Macleod J. Wider collateral damage to children in the UK because of the social distancing measures designed to reduce the impact of COVID-19 in adults. *BMJ Paediatr Open* 2020; **4**: e000701.

8 Newlove-Delgado, T Williams T, Robertson K, McManus S, *et al.* Mental health of children and young people in England, 2021. Wave 2 follow up to the 2017 survey. 2021. https://files.digital.nhs.uk/97/B09EF8/mhcyp_2021_rep.pdf (accessed Dec 15, 2021).

9 Vizard T, Sadler K, Ford T, *et al.* Mental health of children and young people in England, 2020. Wave 1 follow up to the 2017 survey. 2020.

For peer review only - http://bmjopen.bmj.com/site/about/guidelines.xhtml

https://files.digital.nhs.uk/AF/AECD6B/mhcyp_2020_rep_v2.pdf (accessed April 5, 2021).
10 Raw JAL, Waite P, Pearcey S, Shum A, Patalay P, Creswell C. Examining changes in parent-reported child and adolescent mental health throughout the UK's first COVID-19 national lockdown. *J Child Psychol Psychiatry* 2021; **62**: 1391–401.
11 Department for Education. State of the Nation 2021: Children and young people's wellbeing. Research report. 2022. https://assets.publishing.service.gov.uk/government/uploads/system/uploads/attachment_data/file/1053302/State_of_the_Nation_CYP_Wellbeing_2022.pdf (accessed April 7, 2022).
12 Welsh Government. Shielded households (composition and characteristics during the coronavirus (COVID-19) pandemic: July 2020. 2020. https://gov.wales/shielded-households-composition-and-characteristics-during-coronavirus-covid-19-pandemic-july-2020 (accessed Nov 14, 2020).
13 Hysing M, Elgen I, Gillberg C, Lie SA, Lundervold AJ. Chronic physical illness and mental health in children. Results from a large-scale population study. *J Child Psychol Psychiatry* 2007; **48**: 785–92.
14 Griffiths H, O'Connor K, Phillips B, *et al.* Meeting the psychological needs of children in shielding families. 2020. https://www.bps.org.uk/sites/www.bps.org.uk/files/Policy/Policy - Files/Meeting the psychological needs of children in shielding families.pdf (accessed Nov 17, 2020).
15 Welsh Government. Children living in shielded households during the coronavirus (COVID-19) pandemic: June 2020. 2020. https://gov.wales/children-living-shielded-households-during-coronavirus-covid-19-pandemic-june-2020-html (accessed Nov 15, 2020).
16 Kassa C, Pavlopoulou G. Lonely lockdown. Life for siblings of disabled children in the UK. 2021. https://doi.org/10.14324/000.rp.10125424.
17 Lyons RA, Jones KH, John G, *et al.* The SAIL databank: linking multiple health and social care datasets. *BMC Med Inform Decis Mak* 2009; **9**: 3.
18 Rodgers S, Lyons R, Dsilva R, *et al.* Residential Anonymous Linking Fields (RALFs): a novel information infrastructure to study the interaction between the environment and individuals' health. *J Public Health* 2009; **31**: 582–8.
19 Schnier C, Wilkinson T, Akbari A, *et al.* The Secure Anonymised Information Linkage databank Dementia e-cohort (SAIL-DeC). *Int J Popul Data Sci* 2020; **5**: 1121.
20 Schultze A, Bates C, Cockburn J, *et al.* Identifying care home residents in electronic health records - an OpenSAFELY short data report. *Wellcome Open Res* 2021; **6**: 90.
21 Paranjothy S, Evans A, Bandyopadhyay A, *et al.* Risk of emergency hospital admission in children associated with mental disorders and alcohol misuse in the household: an electronic birth cohort study. *Lancet Public Health* 2018; **3**: e279–88.
22 John A, Marchant AL, McGregor JI, *et al.* Recent trends in the incidence of anxiety and prescription of anxiolytics and hypnotics in children and young people: an e-cohort study. *J Affect Disord* 2015; **183**: 134–41.
23 John A, Marchant AL, Fone DL, *et al.* Recent trends in primary-care antidepressant prescribing to children andyoung people: an e-cohort study. *Psychol Med* 2016; **46**: 3315.
24 Welsh Government. Welsh Index of Multiple Deprivation (WIMD) 2019: Results report. 2019. https://gov.wales/sites/default/files/statistics-and-research/2019-11/welsh-index-multiple-deprivation-2019-results-report-024.pdf (accessed Nov 20, 2020).
25 Benchimol EI, Smeeth L, Guttmann A, *et al.* The REporting of studies Conducted

For peer review only - http://bmjopen.bmj.com/site/about/guidelines.xhtml

using Observational Routinely-collected health Data (RECORD) statement. *PLoS Med* 2015; **12**: e1001885.

26 Campbell OLK, Bann D, Patalay P. The gender gap in adolescent mental health: A cross-national investigation of 566,829 adolescents across 73 countries. *SSM – Population Health* 2021; 13, 100742.

27 Ma L, Mazidi M, Li K, et al. Prevalence of mental health problems among children and adolescents during the COVID-19 pandemic: A systematic review and meta-analysis. *Journal of Affective Disorders* 2021; 78-89.

28 O'Connor RC, Wetherall K, Cleare S, et al. Mental health and well-being during the COVID-19 pandemic: longitudinal analyses of adults in the UK COVID-19 Mental Health & Wellbeing study. *The British Journal of Psychiatry* 2021; 218: 326-333.

29 Torjesen I. Covid-19: Only a third of children in need accessed mental health support in the pandemic. *BMJ* 2022; **376**: o335.

30 Young Minds. Coronavirus: Impact on young people with mental health needs. 2020. https://www.youngminds.org.uk/media/04apxfrt/youngminds-coronavirus-report-summer-2020.pdf (accessed Nov 2, 2020).

31 Cornish RP, John A, Boyd A, Tilling K, Macleod J. Defining adolescent common mental disorders using electronic primary care data: A comparison with outcomes measured using the CIS-R. *BMJ Open* 2016; **6**: e013167.

32 Centre for Mental Health. Covid-19 and the nation's mental health: October 2020. 2020. https://www.centreformentalhealth.org.uk/publications/covid-19-and-nations-mental-health-october-2020 (accessed April 8, 2021).

33 Welsh Government. Pledge to support youth with extra £9.4m investment in children and young people mental health services. 2021. https://gov.wales/pledge-support-youth-extra-ps94m-investment-children-and-young-people-mental-health-services (accessed May 2, 2021).

Figure captions

Figure 1. Flow diagram of the inclusion criteria for the creation of three study cohorts: clinically extremely vulnerable children, children living with a clinically extremely vulnerable person, and children in the general population. CEV, clinically extremely vulnerable; CVSP, COVID-19 Shielded People List; SAIL, Secure Anonymised Information Linkage; GP, general practice; RALF, residential anonymised linking field; NHS, national health service.

Figure 2. Kaplan-Meier survival curves for each cohort, showing the time to first record of anxiety or depression between 23rd March 2020 and 31st January 2021 (2020/21)

Figure 3. Kaplan-Meier survival curves for each cohort, showing the time to first record of anxiety or depression between 23rd March 2019 and 31st January 2020 (2019/20)

For peer review only - http://bmjopen.bmj.com/site/about/guidelines.xhtml

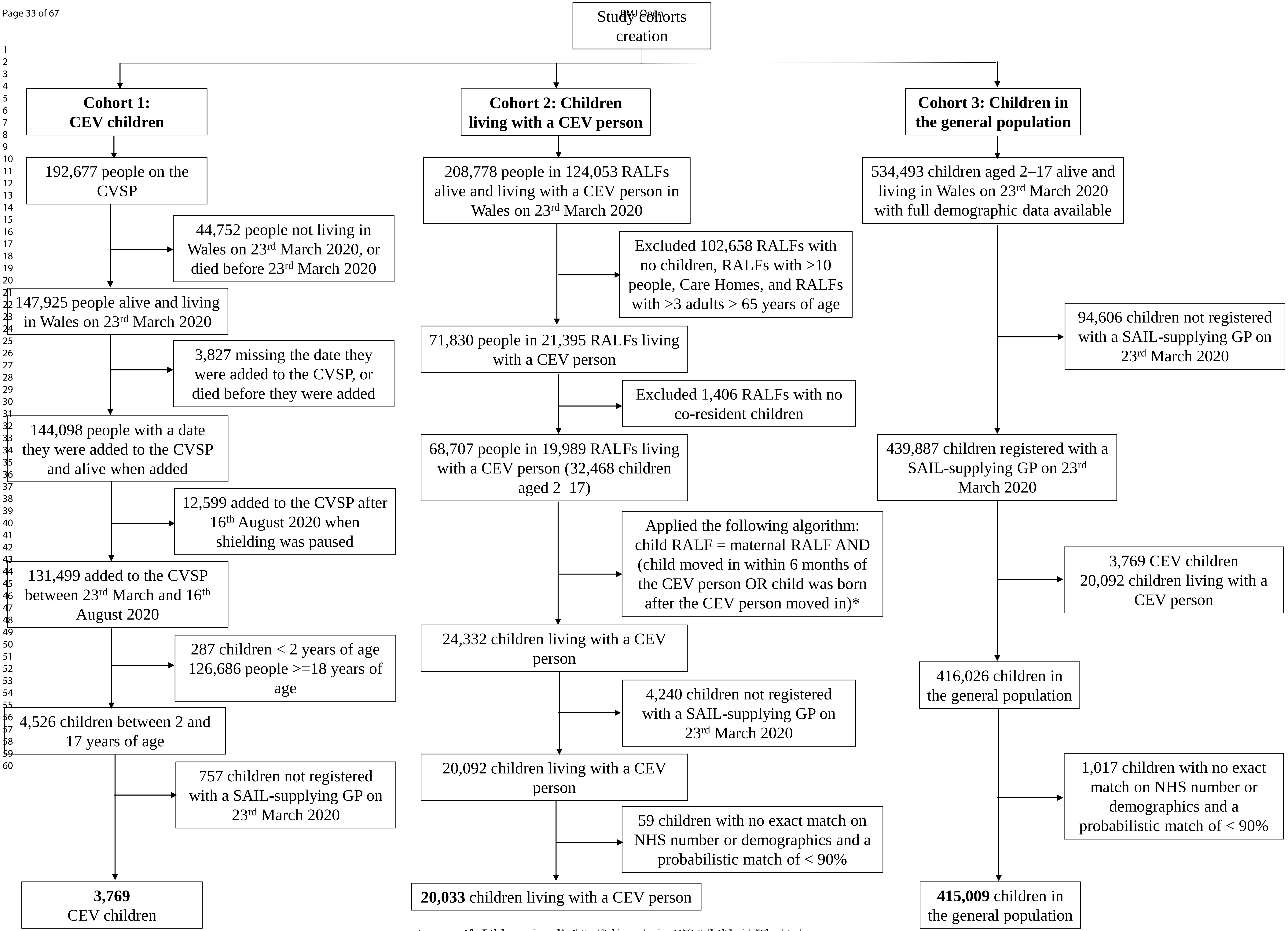

*except if children were living with younger CEV children. These children were included if they shared the same maternal ALF

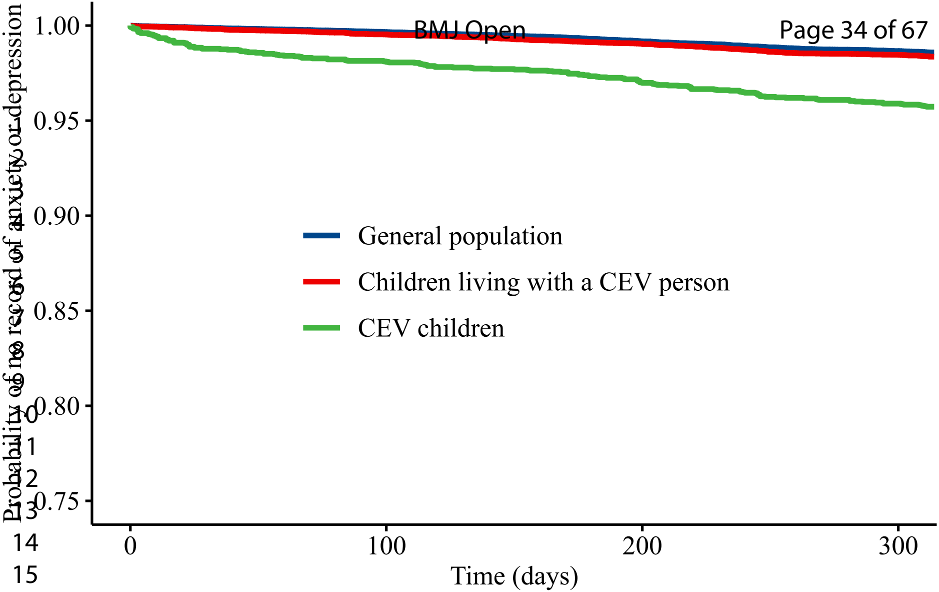

Number at risk

	0	100	200	300
General population	415009	410665	401954	396973
Children living with a CEV person	20033	19844	19451	19231
CEV children	3769	3675	3581	3514

For peer review only - http://bmjopen.bmj.com/site/about/guidelines.xhtml

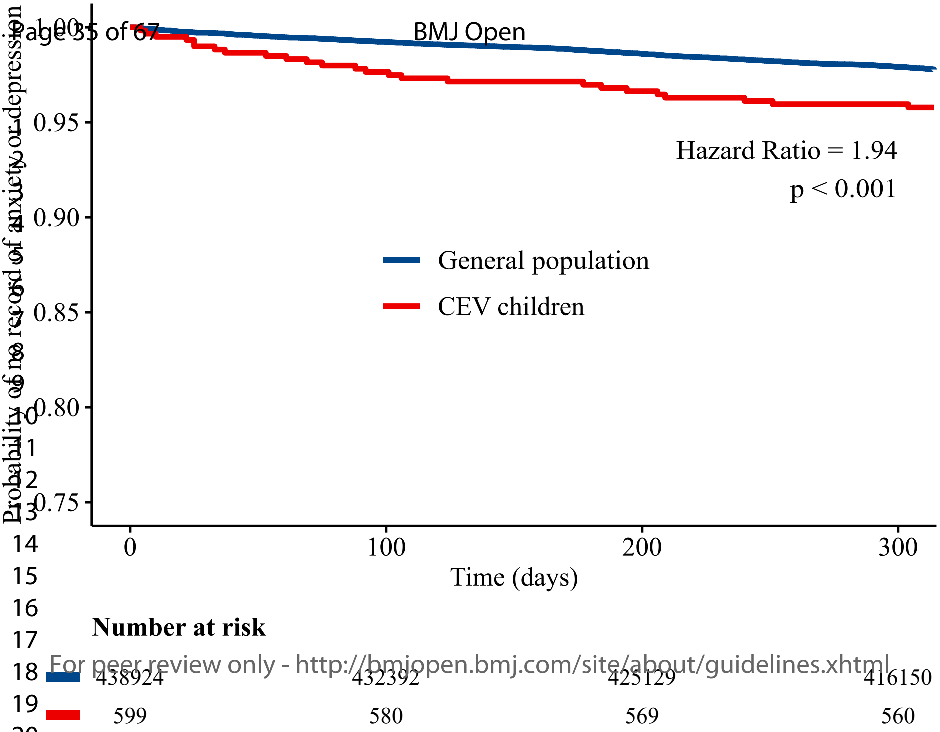

For peer review only - http://bmjopen.bmj.com/site/about/guidelines.xhtml

Appendix

SAIL databank additional information

SAIL databank information governance policies and procedures

The SAIL databank (www.saildatabank.com) is an internationally recognised, remotely-accessible, privacy-protecting data safe haven designed to support observational, interventional, and policy-relevant research to improve population health, well-being, and services.[1–6] SAIL contains anonymised, linkable, routinely collected health, administrative, and social care data for the population of Wales, UK, from multiple sources at individual, household, and ecological levels.[1–6] Data are anonymised using a split-file process, which has been described in detail elsewhere.[1–3] Within each dataset, identifiable and non-identifiable data are separated, and identifiable data are sent to a trusted third party (TTP), Digital Health and Care Wales (DHCW; previously known as the NHS Wales Informatics Service). The TTP uniquely matches identities based on name, NHS number, date of birth, and Unique Property Reference Number (UPRN), using the Matching Algorithm for Consistent Results in Anonymised Linkage, which has an accuracy of 99.85%.[1,2] Individuals and residences are then assigned unique identifiers: for individuals this is called an Anonymised Linking Field (ALF) and for residences a Residential Anonymised Linking Field (RALF). The anonymised and non-identifiable data components are then recombined within SAIL and the linking fields are further encrypted and used to anonymously link between datasets. This enables data from multiple sources, including general practice (GP) data, hospital admissions, outpatient data, and demographic details to be linked at the individual and household level, while preserving anonymity.

References

For peer review only - http://bmjopen.bmj.com/site/about/guidelines.xhtml

1. Lyons RA, Jones KH, John G, et al. The SAIL databank: linking multiple health and social care datasets. BMC Med Inform Decis Mak2009;9:3.doi:10.1186/1472-6947-9-3
2. Ford DV, Jones KH, Verplancke JP, et al. The SAIL Databank: building a national architecture for e-health research and evaluation. BMC Health Serv Res2009;9:157.doi:10.1186/1472-6963-9-157
3. Rodgers SE, Lyons RA, Dsilva R, et al. Residential Anonymous Linking Fields (RALFs): a novel information infrastructure to study the interaction between the environment and individuals health. J Public Health2009;31:582–8.doi:10.1093/pubmed/fdp041
4. Rodgers SE, Demmler JC, Dsilva R, Lyons RA. Protecting health data privacy while using residence-based environment and demographic data. *Health Place*. 2012; 18: 209-217.
5. Jones, K. H., Ford, D. V., Thompson, S., & Lyons, R. A. (2019). A profile of the SAIL databank on the UK secure research platform. *International Journal of Population Data Science*, *4*(2). https://doi.org/10.23889/ijpds.v4i2.1134
6. Jones, K. H., Ford, D. V., Jones, C., Dsilva, R., Thompson, S., Brooks, C. J., Heaven, M. L., Thayer, D. S., McNerney, C. L., & Lyons, R. A. (2014). A case study of the secure anonymous information linkage (SAIL) gateway: A privacy-protecting remote access system for health-related research and evaluation. *Journal of Biomedical Informatics*, *50*, 196–204. https://doi.org/10.1016/j.jbi.2014.01.003

For peer review only - http://bmjopen.bmj.com/site/about/guidelines.xhtml

Ethical Approval

The Information Governance Review Panel (IGRP) is an independent panel of representatives from various government, regulatory, and professional organisations, who review all proposals for SAIL data access to ensure that they are appropriate with respect to Information Governance, and in the public interest.[1] Data were analysed within the SAIL secure research environment, and appropriate disclosure control procedures were followed to ensure that no personally identifiable data or small numbers (n<5) were removed from the environment. All data within SAIL are treated in accordance with the Data Protection Act 2018 and SAIL complies with the principles of the General Data Protection Regulation (GDPR).

References

1. Ford DV, Jones KH, Verplancke JP, et al. The SAIL Databank: building a national architecture for e-health research and evaluation. BMC Health Serv Res2009;9:157.doi:10.1186/1472-6963-9-157

For peer review only - http://bmjopen.bmj.com/site/about/guidelines.xhtml

ICD-10 codes for cancers of the blood or bone marrow

ICD-10 code	Description
C81	Hodgkin lymphoma
C82	Follicular lymphoma
C83	Non-follicular lymphoma
C84	Mature T/NK-cell lymphomas
C85	Other and unspecified types of non-Hodgkin lymphoma
C86	Other specified types of T/NK-cell lymphoma
C88	Malignant immunoproliferative diseases
C90	Multiple myeloma and malignant plasma cell neoplasms
C91	Lymphoid leukaemia
C92	Myeloid leukaemia
C93	Monocytic leukaemia
C94	Other leukaemias of specified cell type
C95	Leukaemia of unspecified cell type
C96	Other and unspecified malignant neoplasms of lymphoid, haematopoietic and related tissue
D470	Histiocytic and mast cell tumours of uncertain and unknown behaviour
D475	Chronic eosinophilic leukaemia [hypereosinophilic syndrome]
D477	Other specified neoplasms of uncertain or unknown behaviour of lymphoid, haematopoietic and related tissue
D479	Neoplasm of uncertain or unknown behaviour of lymphoid, haematopoietic and related tissue, unspecified
D595	Paroxysmal nocturnal haemoglobinuria [Marchiafava-Micheli]
D71X	Functional disorders of polymorphonuclear neutrophils
D730	Hyposplenism
D760	Langerhans' cell histiocytosis, not elsewhere classified
D761	Haemophagocytic lymphohistiocytosis
D898	Other specified disorders involving the immune mechanism, not elsewhere classified
D899	Disorder involving the immune mechanism, unspecified
L412	Lymphomatoid papulosis
P615	Transient neonatal neutropenia

For peer review only

ICD-10 codes for respiratory illnesses

ICD-10 code	Description
E84	Cystic Fibrosis
J84	Other interstitial pulmonary diseases
J620	Pneumoconiosis due to talc dust
J630	Aluminosis (of lung)
J631	Bauxite fibrosis (of lung)
J633	Graphite fibrosis (of lung)
J634	Siderosis
J635	Stannosis

For peer review only - http://bmjopen.bmj.com/site/about/guidelines.xhtml

J660	Byssinosis
J661	Flax-dresser disease
J662	Cannabinosis
J668	Airway disease due to other specific organic dusts
J670	Farmer lung
J671	Bagassosis
J678	Hypersensitivity pneumonitis due to other organic dusts
J684	Chronic respiratory conditions due to chemicals, gases, fumes and vapours
J688	Other respiratory conditions due to chemicals, gases, fumes and vapours
J698	Pneumonitis due to other solids and liquids
J701	Chronic and other pulmonary manifestations due to radiation
J703	Chronic drug-induced interstitial lung disorders
J840	Alveolar and parietoalveolar conditions
J983	Compensatory emphysema
J991	Respiratory disorders in other diffuse connective tissue disorders
M313	Wegener granulomatosis
P250	Interstitial emphysema originating in the perinatal period
Q334	Congenital bronchiectasis

OPCS-4 codes for immunosuppression therapy

OPCS-4 code	Description
X353	Active Inflammatory thyroid eye disease patients currently on weekly intravenous steroid infusion treatment (12 weekly injections regime) or immunospressant
X374	Intramuscular Immunotherapy
X385	Subcutaneous Immunotherapy
X891	Monoclonal antibodies Band 1
X892	Monoclonal antibodies Band 2
X893	Patients receiving maintenance treatment with rituximab, obinotuzimab or ofatumumab
X894	Somatostatin analogues Band 1
X895	Allergic emergency drugs Band 1
X961	Patients previously treated for haematological malignancy requiring IV immunoglobulin replacement
X962	Allergen immunotherapy drugs Band 1
X963	Poison management drugs Band 1

For peer review only - http://bmjopen.bmj.com/site/about/guidelines.xhtml

Pre-COVID-19 study cohorts

Figure 3 shows a flow diagram of inclusion criteria for the two 2019 study cohorts: children in the general population, and clinically extremely vulnerable children with blood or bone cancer or respiratory illnesses, or receiving immunosuppression therapy.

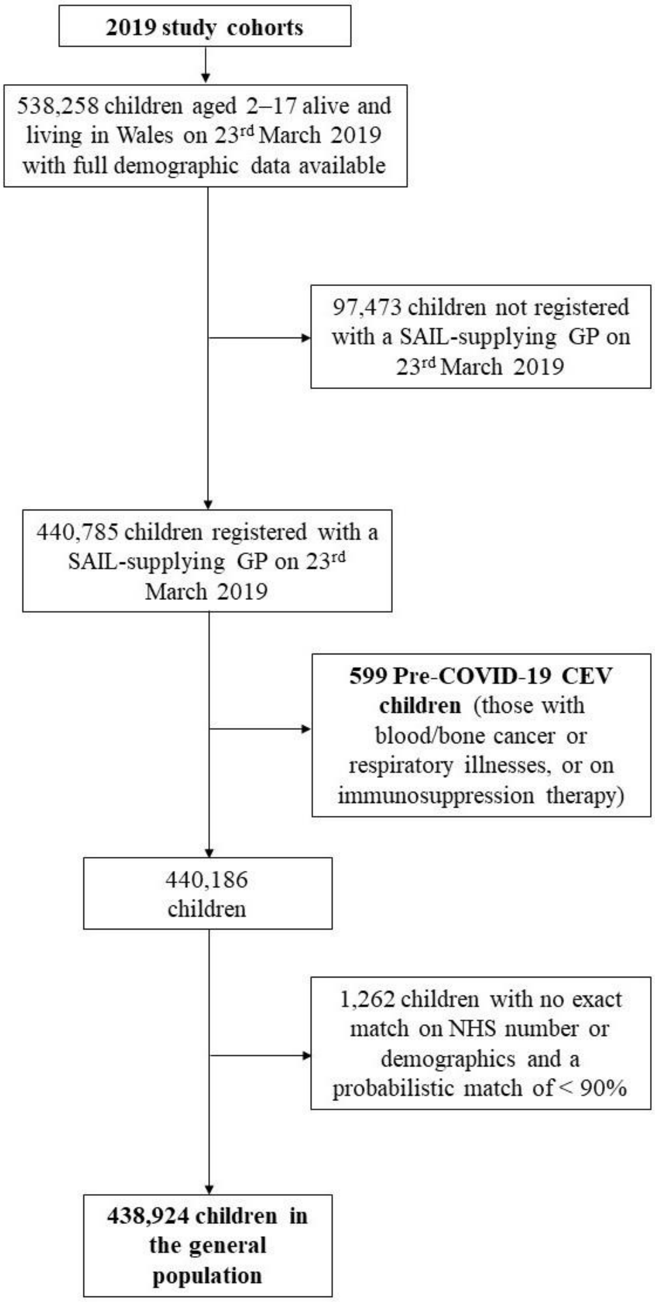

Figure 3. Flow diagram of the inclusion criteria for the creation of the 2019 study cohorts

For peer review only - http://bmjopen.bmj.com/site/about/guidelines.xhtml

Sensitivity Analysis

We undertook a sensitivity analysis to confirm the validity of creating a cohort of pre-COVID-19 CEV children based on just three of the categories included in the COVID-19 Shielded People List (CVSP). We created a cohort of clinically extremely vulnerable (CEV) children who were added to the CVSP in 2020 for the same three reasons only (respiratory illnesses, blood/bone cancer, and immunosuppression therapy) and a cohort of children in the general population (figure 1). Demographic characteristics of the children in each cohort are presented in Table 1. We plotted the Kaplan-Meier survival curve for each cohort and estimated the hazard ratio of having a record for anxiety or depression during the COVID-19 pandemic (March 23rd 2020–January 31st 2021) using Cox regression (figure 2).

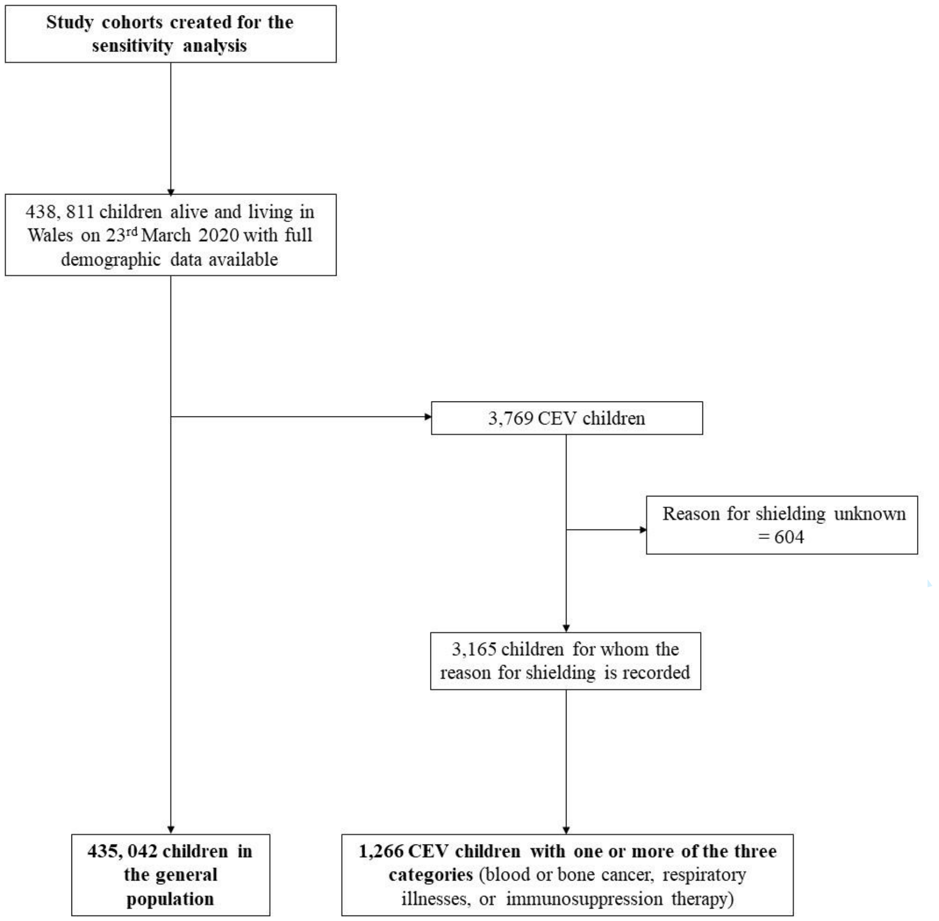

Figure 1. Flow diagram of the inclusion criteria for the creation of the study cohorts for the sensitivity analysis.

For peer review only - http://bmjopen.bmj.com/site/about/guidelines.xhtml

		General population 2020	CEV children with one or more of three categories 2020	Chi^2 P value
N		435,042	1,266	
**Sex (%)**	Male	222,558 (51.2)	712 (56.2)	<0.001
	Female	212,484 (48.8)	554 (43.8)	
**Age group (%)**	2–7	157,691 (36.2)	385 (30.4)	<0.001
	8–12	143,988 (33.1)	396 (31.3)	
	13–17	133,363 (30.7)	485 (38.3)	
**Deprivation quintile (WIMD 2019) (%)**	1 (most deprived)	111,133 (25.5)	329 (26.0)	0.262
	2	92,037 (21.2)	257 (20.3)	
	3	76,717 (17.6)	210 (16.6)	
	4	73,277 (16.8)	203 (16.0)	
	5 (least deprived)	81,878 (18.8)	267 (21.1)	
**Rural/Urban area (%)**	Rural	116,126 (26.7)	336 (26.5)	0.928
	Urban	318,916 (73.3)	930 (73.5)	
**Any history of anxiety or depression**	NO	415,925 (95.6)	1,166 (92.1)	<0.001
	YES	19,117 (4.4)	100 (7.9)	

Table 1. Demographic characteristics of children in the general population and clinically extremely vulnerable children added to the COVID-19 Shielded People List for one or more of three reasons (blood or bone cancer, respiratory illnesses, or immunosuppression therapy)

The results showed a similar pattern to those obtained when analysing the full CEV cohort in that CEV children with one or more of the three categories were at greater risk of having a record for anxiety or depression during the COVID-19 pandemic (March 23rd 2020–January 31st 2021) compared to children in the general population, thereby validating our approach.

For peer review only - http://bmjopen.bmj.com/site/about/guidelines.xhtml

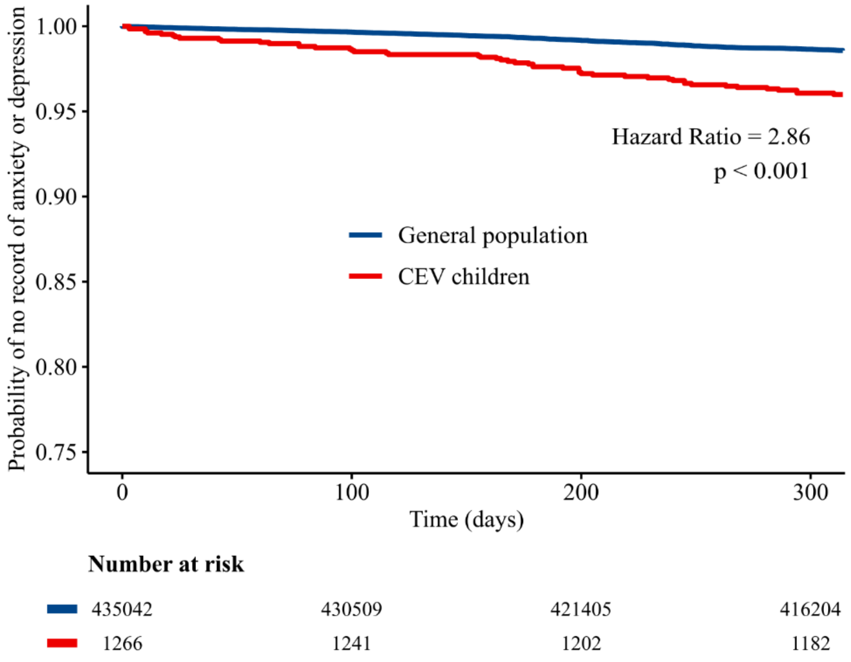

Figure 2. Kaplan-Meier survival curves for each cohort, showing the time to first record of anxiety or depression during the COVID-19 pandemic

CEV, clinically extremely vulnerable

For peer review only - http://bmjopen.bmj.com/site/about/guidelines.xhtml

Read v2 and ICD-10 diagnosis codes used to identify records of anxiety or depression in primary or secondary care

Read v2 codes for depression diagnosis

Read v2 code	Description
Eu32.	[X]Depressive episode
Eu320	[X]Mild depressive episode
Eu321	[X]Moderate depressive episode
Eu322	[X]Severe depressive episode without psychotic symptoms
Eu324	[X]Mild depression
Eu32y	[X]Other depressive episodes
Eu32z	[X]Depressive episode, unspecified
Eu33.	[X]Recurrent depressive disorder
Eu330	[X]Recurrent depressive disorder, current episode mild
Eu331	[X]Recurrent depressive disorder, current episode moderate
Eu332	[X]Recurrent depressive disorder, current episode severe without psychotic symptoms
Eu334	[X]Recurrent depressive disorder, currently in remission
Eu33y	[X]Other recurrent depressive disorders
Eu33z	[X]Recurrent depressive disorder, unspecified
Eu341	[X]Dysthymia
E118.	Seasonal affective disorder
E135.	Agitated depression
E2B..	Depressive disorder NEC
E2B1.	Chronic depression
E291.	Prolonged depressive reaction
E204.	Neurotic depression reactive type
E2B0.	Postviral depression
E112.	Single major depressive episode
E1120	Single major depressive episode, unspecified
E1121	Single major depressive episode, mild
E1122	Single major depressive episode, moderate
E1123	Single major depressive episode, severe, without psychosis
E1125	Single major depressive episode, partial or unspecied remission
E1126	Single major depressive episode, in full remission
E112z	Single major depressive episode NOS
E113.	Recurrent major depressive episode
E1130	Recurrent major depressive episodes, unspecified
E1131	Recurrent major depressive episodes, mild
E1132	Recurrent major depressive episodes, moderate
E1133	Recurrent major depressive episodes, severe, no psychosis
E1135	Recurrent major depressive episodes, partial/unspecified remission
E1136	Recurrent major depressive episodes, in full remission
E1137	Recurrent depression
E113z	Recurrent major depressive episode NOS

For peer review only - http://bmjopen.bmj.com/site/about/guidelines.xhtml

Read v2 codes for depression symptoms

Read v2 code	Description
1B17.	Depressed
1B1U.	Symptoms of depression
1BQ..	Loss of capacity for enjoyment
1BT..	Depressed mood
1BU..	Loss of hope for the future
2257	O/E – depressed
1BP..	Loss of interest

Read v2 codes for antidepressant prescriptions

Read v2 code	Description
d71..	Amitriptyline hydrochloride
d72..	Butriptyline - discontinued
d73..	Clomipramine hydrochloride
d74..	Desipramine hydrochloride
d75..	Dosulepin Hydrochloride
d76..	Doxepin
d77..	Imipramine hydrochloride
d78..	Iprindole
d79..	Lofepramine
d7a..	Maprotiline hydrochloride
d7b..	Mianserin hydrochloride
d7c..	Nortriptyline
d7d..	Protriptyline hydrochloride
d7e..	Trazadone hydrochloride
d7f..	Trimipramine
d7g..	Viloxazine hydrochloride
d7h..	Amoxapine
d81..	Phenelzine
d83..	Isocarboxazid
d84..	Tranylcypromine
d85..	Moclobemide
d91..	Compound Antidepressants A-Z
da1..	Flupentixol [Antidepressant]
da2..	Tryptophan
da3..	Fluvoxamine Maleate
da4..	Fluoxetine hydrochloride
da5..	Sertraline hydrochloride
da6..	Paroxetine hydrochloride
da7..	Venlafaxine
da9..	Citalopram
daA..	Reboxetine
daB..	Mirtazapine

For peer review only - http://bmjopen.bmj.com/site/about/guidelines.xhtml

daC..	Escitalopram
daD..	Agomelatine
gde..	Duloxetine
d911.	Limbitrol 5 capsules - discontinued
d912.	Limbitrol 10 capsules - discontinued
d913.	Motipress tablets
x28CP	Discontinued
d914.	Motival tablets discontinued
d916.	Triptafen tablets - only one not discontinued?
d917.	Triftafen-M tablets - discontinued
d8…	Monoamine-oxidase
d82..	Iproniazid

Read v2 codes for anxiety diagnosis

Read v2 code	Description
Eu41.	[X]Other anxiety disorders
Eu410	[X]Panic disorder [episodic paroxysmal anxiety]
Eu411	[X]Generalized anxiety disorder
Eu413	[X]Other mixed anxiety disorders
Eu41y	[X]Other specified anxiety disorders
Eu41z	[X]Anxiety disorder, unspecified
E200.	Anxiety states
E2000	Anxiety state unspecified
E2001	Panic disorder
E2002	Generalised anxiety disorder
E2004	Chronic anxiety
E2005	Recurrent anxiety
E200z	Anxiety state NOS
E202	Phobic disorders
Eu40.	Phobic anxiety disorder
Eu930	[X]Separation anxiety disorder of childhood *
Eu931	[X]Phobic anxiety disorder of childhood*
Eu932	[X]Social anxiety disorder of childhood*
E2D0.	Disturbance of anxiety and fearfulness in childhood and adolescence*
E2D00	Childhood and adolescent overanxiousness disturbance*
E2D0z	Disturbance of anxiety and fearfulness in childhood and adolescence NOS*

Read v2 codes for anxiety symptoms

Read v2 code	Description
1B13.	Anxiousness
2258	O/E - anxious
1B12.	Nerves, nervousness
R2y2.	(D) nervousness

For peer review only - http://bmjopen.bmj.com/site/about/guidelines.xhtml

2259	O/E nervous
225J.	O/E panic attack
1B1V.	C/O panic attack

Read v2 codes for anxiety prescriptions – hypnotics

Read v2 code	Description
d11..	Chloral hydrate
d12..	Clomethiazole edisylate (hypnotic)
d13..	Dichloralphenazone - discontinued
d14..	Flumtrazepam - discontinued
d15..	Flurazepam
d16..	Loprazolam
d17..	Lormetazepam
d18..	Nitrazepam
d1a..	Temazepam (hynotic)
d1b..	Triazolam - discontinued
d1c..	Triclofos sodium
d1d..	Zopiclone
d1f..	Zolpidem
d1g..	Zaleplon
d1h..	Melatonin
d1i..	Dexmedetomidine

Read v2 codes for anxiety prescriptions – hypnotics

Read v2 code	Description
d21..	Diazepam
d22..	Alprazolam
d23..	Bromazepam
d24..	Chlordiazepoxide
d25..	Chlormezanone
d26..	Clobazam
d27..	Clorazepate dipotassium
d28..	Hydroxyzine hcl (anxiolytic)
d29..	Ketazolam - discontinued
d2a..	Lorazepam (anxiolytic)
d2b..	Medazepam - discontinued
d2c..	Meprobamate
d2d..	Oxazepam
d2e..	Prazepam - discontinued
d2f..	Buspirone hydrocholoride
d2g..	Flumazenil

For peer review only - http://bmjopen.bmj.com/site/about/guidelines.xhtml

Read v2 codes for mixed anxiety and depression diagnosis

Read v2 code	Description
E2003	Anxiety with depression
Eu412	[X]Mixed anxiety and depressive disorder

ICD-10 diagnosis codes for depression

ICD-10 code	Description
F321	Moderate depressive episode
F322	Severe depressive episode without psychotic symptoms
F328	Other depressive episodes
F329	Depressive episode, unspecified
F33	Recurrent depressive disorder
F330	Recurrent depressive disorder, current episode mild
F331	Recurrent depressive disorder, current episode moderate
F332	Recurrent depressive disorder, current episode severe without psychotic symptoms
F334	Recurrent depressive disorder, currently in remission
F338	Other recurrent depressive disorders
F339	Recurrent depressive disorder, unspecified
F341	Dysthymia

ICD-10 diagnosis codes for anxiety and mixed anxiety and depression

ICD-10 code	Description
F40	Phobic anxiety disorders
F400	Agoraphobia
F401	Social phobias
F402	Specific (isolated) phobias
F408	Other phobic anxiety disorders
F41	Other anxiety disorders
F410	Panic disorder (episodic paroxysmal anxiety)
F411	Generalized anxiety disorder
F413	Other mixed anxiety disorders
F418	Other specified anxiety disorders
F419	Anxiety disorder, unspecified
F930	Separation anxiety disorder of childhood
F931	Phobic anxiety disorder of childhood
F932	Social anxiety disorder of childhood
F412	Mixed anxiety and depressive disorder

For peer review only - http://bmjopen.bmj.com/site/about/guidelines.xhtml

The RECORD statement – checklist of items, extended from the STROBE statement, that should be reported in observational studies using routinely collected health data.

	Item No.	STROBE items	Location in manuscript where items are reported	RECORD items	Location in manuscript where items are reported
**Title and abstract**					
	1	(a) Indicate the study's design with a commonly used term in the title or the abstract (b) Provide in the abstract an informative and balanced summary of what was done and what was found	(a) The study design is indicated as a data linkage study in the title (b) We have provided an informative and balanced summary in the abstract	RECORD 1.1: The type of data used should be specified in the title or abstract. When possible, the name of the databases used should be included.<br><br>RECORD 1.2: If applicable, the geographic region and timeframe within which the study took place should be reported in the title or abstract.<br><br>RECORD 1.3: If linkage between databases was conducted for the study, this should be clearly stated in the title or abstract.	1.1 The type of data used is specified in the abstract. It was not possible to name all of the databases used in the abstract.<br>1.2 We have reported the geographic region within which the study took place in the title and the abstract, and the timeframe in the abstract<br>1.3 It is clearly stated in the title and the abstract that linkage between databases was conducted for the study
**Introduction**					
Background rationale	2	Explain the scientific background and rationale	The scientific background and rationale for the		

For peer review only - http://bmjopen.bmj.com/site/about/guidelines.xhtml

		for the investigation being reported	investigation is explained in the introduction		
Objectives	3	State specific objectives, including any prespecified hypotheses	The objectives are stated in the introduction		
**Methods**					
Study Design	4	Present key elements of study design early in the paper	The study design is reported at the beginning of the methods section, under the subheading "study design and data source"		
Setting	5	Describe the setting, locations, and relevant dates, including periods of recruitment, exposure, follow-up, and data collection	The setting, locations, and relevant dates are described in the "study population and setting" section		
Participants	6	*(a) Cohort study* - Give the eligibility criteria, and the sources and methods of selection of participants. Describe methods of follow-up *Case-control study* - Give the eligibility criteria, and the sources and methods of case ascertainment and control selection. Give the rationale for the choice of cases and controls	(a) Eligibility criteria, and the sources and methods of selection and follow-up of participants are given in the "study population and setting" section<br>(b) N/A this was not a matched study	RECORD 6.1: The methods of study population selection (such as codes or algorithms used to identify subjects) should be listed in detail. If this is not possible, an explanation should be provided.<br><br>RECORD 6.2: Any validation studies of the codes or algorithms used to select the population should be referenced. If validation was conducted for this study and not	6.1 We have described in detail the methods of study population selection in the "study population and setting" section and the "measures" section<br><br>6.2 We have referenced the validation studies of the anxiety and

For peer review only - http://bmjopen.bmj.com/site/about/guidelines.xhtml

		Cross-sectional study - Give the eligibility criteria, and the sources and methods of selection of participants<br><br>*(b) Cohort study* - For matched studies, give matching criteria and number of exposed and unexposed<br>*Case-control study* - For matched studies, give matching criteria and the number of controls per case		published elsewhere, detailed methods and results should be provided.<br><br>RECORD 6.3: If the study involved linkage of databases, consider use of a flow diagram or other graphical display to demonstrate the data linkage process, including the number of individuals with linked data at each stage.	depression codes we have used in our study in the "measures" section<br><br>6.3 We have provided a flow diagram with the number of individuals included in each cohort at each stage of linkage (Figure 1)
Variables	7	Clearly define all outcomes, exposures, predictors, potential confounders, and effect modifiers. Give diagnostic criteria, if applicable.	We have clearly defined all relevant measures in the "measures" section	RECORD 7.1: A complete list of codes and algorithms used to classify exposures, outcomes, confounders, and effect modifiers should be provided. If these cannot be reported, an explanation should be provided.	7.1 We have provided a list of codes used in the study in the appendix
Data sources/ measurement	8	For each variable of interest, give sources of data and details of methods of assessment (measurement). Describe comparability of assessment methods if there is more than one group	We have described all data sources and measures in the "study design and data sources" section and the "measures" section		

For peer review only - http://bmjopen.bmj.com/site/about/guidelines.xhtml

Bias	9	Describe any efforts to address potential sources of bias	We have explained that we used a conservative approach to identify cohort participants in the "study population and setting" section		
Study size	10	Explain how the study size was arrived at	We have included a flowchart (Figure 1) to describe the creation of each of the study cohorts and explained the process of cohort creation, referred to in the "study population and setting" section		
Quantitative variables	11	Explain how quantitative variables were handled in the analyses. If applicable, describe which groupings were chosen, and why	Handling of quantitative variables is described in the "measures" section		
Statistical methods	12	(a) Describe all statistical methods, including those used to control for confounding<br>(b) Describe any methods used to examine subgroups and interactions<br>(c) Explain how missing data were addressed<br>(d) *Cohort study* - If applicable, explain how	(a) Statistical methods are described in the "statistical analysis" section<br>(b) N/A<br>(c) N/A<br>(d) N/A<br>(e) Sensitivity analyses are described in the appendix		

For peer review only - http://bmjopen.bmj.com/site/about/guidelines.xhtml

		loss to follow-up was addressed<br>*Case-control study* - If applicable, explain how matching of cases and controls was addressed<br>*Cross-sectional study* - If applicable, describe analytical methods taking account of sampling strategy<br>(e) Describe any sensitivity analyses			
Data access and cleaning methods		..		RECORD 12.1: Authors should describe the extent to which the investigators had access to the database population used to create the study population.<br><br>RECORD 12.2: Authors should provide information on the data cleaning methods used in the study.	12.1 We described the extent to which the investigators had access to the database population in the “data access and cleaning methods” section<br>12.2 We provided information on the data cleaning methods in the “data access and cleaning methods” section
Linkage		..		RECORD 12.3: State whether the study included person-level, institutional-level, or other data linkage across two or more databases. The methods of	12.3 The methods of linkage and quality evaluation are detailed in the “study design and data

For peer review only - http://bmjopen.bmj.com/site/about/guidelines.xhtml

				linkage and methods of linkage quality evaluation should be provided.	source" section and the "study population and setting" section and the flowchart (Figure 1)
**Results**					
Participants	13	(a) Report the numbers of individuals at each stage of the study (*e.g.*, numbers potentially eligible, examined for eligibility, confirmed eligible, included in the study, completing follow-up, and analysed)<br>(b) Give reasons for non-participation at each stage.<br>(c) Consider use of a flow diagram	(a) Numbers of individuals at each study stage are provided in the flow diagrams (Figure 1)<br>(b) Reasons for exclusion are provided on the flowchart (Figure 1)<br>(c) We used a flowchart to illustrate selection of our study cohorts (Figure 1)	RECORD 13.1: Describe in detail the selection of the persons included in the study (*i.e.*, study population selection) including filtering based on data quality, data availability and linkage. The selection of included persons can be described in the text and/or by means of the study flow diagram.	13.1 We described in detail the selection of the persons included in the study in the "study population and setting" section and Figure 1
Descriptive data	14	(a) Give characteristics of study participants (*e.g.*, demographic, clinical, social) and information on exposures and potential confounders<br>(b) Indicate the number of participants with missing data for each variable of interest	(a) Characteristics of study participants are given in Table 2<br>(b) N/A<br>(c) N/A		

For peer review only - http://bmjopen.bmj.com/site/about/guidelines.xhtml

		(c) *Cohort study* - summarise follow-up time (*e.g.*, average and total amount)			
Outcome data	15	*Cohort study* - Report numbers of outcome events or summary measures over time<br>*Case-control study* - Report numbers in each exposure category, or summary measures of exposure<br>*Cross-sectional study* - Report numbers of outcome events or summary measures	Numbers of outcome events are reported in the results section in Table 4		
Main results	16	(a) Give unadjusted estimates and, if applicable, confounder-adjusted estimates and their precision (e.g., 95% confidence interval). Make clear which confounders were adjusted for and why they were included<br>(b) Report category boundaries when continuous variables were categorized	(a) We report unadjusted and adjusted estimates in the results section, and have explained why we adjusted for each factor<br>(b) N/A<br>(c) N/A		

For peer review only - http://bmjopen.bmj.com/site/about/guidelines.xhtml

		(c) If relevant, consider translating estimates of relative risk into absolute risk for a meaningful time period			
Other analyses	17	Report other analyses done—e.g., analyses of subgroups and interactions, and sensitivity analyses	We report on sensitivity analyses in the appendix		
**Discussion**					
Key results	18	Summarise key results with reference to study objectives	Key results are summarised at the beginning of the discussion		
Limitations	19	Discuss limitations of the study, taking into account sources of potential bias or imprecision. Discuss both direction and magnitude of any potential bias	Limitations are discussed in the 'Strengths and limitations' section including potential underestimation of our cohorts	RECORD 19.1: Discuss the implications of using data that were not created or collected to answer the specific research question(s). Include discussion of misclassification bias, unmeasured confounding, missing data, and changing eligibility over time, as they pertain to the study being reported.	We discussed the limitations of the Shielded Patient List in the 'Strengths and Limitations' section
Interpretation	20	Give a cautious overall interpretation of results considering objectives, limitations, multiplicity of analyses, results from	We have given a cautious and balanced interpretation of the results with reference to the previous literature in the discussion		

For peer review only - http://bmjopen.bmj.com/site/about/guidelines.xhtml

		similar studies, and other relevant evidence			
Generalisability	21	Discuss the generalisability (external validity) of the study results	Discussed in the “strengths and limitations” section		
**Other Information**					
Funding	22	Give the source of funding and the role of the funders for the present study and, if applicable, for the original study on which the present article is based	We have provided the funding source, and the role of the funders in the “Role of the funders” section		
Accessibility of protocol, raw data, and programming code		..		RECORD 22.1: Authors should provide information on how to access any supplemental information such as the study protocol, raw data, or programming code.	We have provided information on how to access data within the SAIL databank

*Reference: Benchimol EI, Smeeth L, Guttmann A, Harron K, Moher D, Petersen I, Sørensen HT, von Elm E, Langan SM, the RECORD Working Committee. The REporting of studies Conducted using Observational Routinely-collected health Data (RECORD) Statement. *PLoS Medicine* 2015; in press.

*Checklist is protected under Creative Commons Attribution (CC BY) license.

Demographic characteristics of the 2019 study population

		General population 2019	CEV children 2019	Chi^2 P value
N		438,924	599	
**Sex (%)**	Male	224,754 (51.2)	347 (57.9)	<0.001
	Female	214,170 (48.8)	252 (42.1)	
**Age group (%)**	2–7	162,730 (37.1)	231 (38.6)	0.412
	8–12	143,844 (32.8)	181 (30.2)	
	13–17	132,350 (30.2)	187 (31.2)	
**Deprivation quintile (WIMD 2019) (%)**	1 (most deprived)	111,381 (25.4)	134 (22.4)	0.003
	2	93,046 (21.2)	137 (22.9)	
	3	77,600 (17.7)	88 (14.7)	
	4	74,288 (16.9)	94 (15.7)	
	5 (least deprived)	82,609 (18.8)	146 (24.4)	
**Rural/Urban area (%)**	Rural	118,418 (27.0)	165 (27.5)	0.790
	Urban	320,506 (73.0)	434 (72.5)	
**Any history of anxiety or depression**	NO	421,659 (96.1)	565 (94.3)	0.037
	YES	17,265 (3.9)	34 (5.7)	

For peer review only

For peer review only - http://bmjopen.bmj.com/site/about/guidelines.xhtml

The RECORD statement – checklist of items, extended from the STROBE statement, that should be reported in observational studies using routinely collected health data.

	Item No.	STROBE items	Location in manuscript where items are reported	RECORD items	Location in manuscript where items are reported
**Title and abstract**					
	1	(a) Indicate the study's design with a commonly used term in the title or the abstract (b) Provide in the abstract an informative and balanced summary of what was done and what was found	(a) The study design is indicated as a data linkage study in the title on page 1<br>(b) We have provided an informative and balanced summary in the abstract on page 2	RECORD 1.1: The type of data used should be specified in the title or abstract. When possible, the name of the databases used should be included.<br><br>RECORD 1.2: If applicable, the geographic region and timeframe within which the study took place should be reported in the title or abstract.<br><br>RECORD 1.3: If linkage between databases was conducted for the study, this should be clearly stated in the title or abstract.	1.1 The type of data used is specified in the abstract on page 2. It was not possible to name all of the databases used in the abstract.<br>1.2 We have reported the geographic region within which the study took place in the title on page 1 and the abstract on page 2, and the timeframe in the abstract on page 2<br>1.3 It is clearly stated in the title (page 1) and the abstract (page 2) that linkage between databases was conducted for the study
**Introduction**					
Background rationale	2	Explain the scientific background and rationale	The scientific background and rationale for the investigation is explained		

		for the investigation being reported	in the introduction on pages 4-5		
Objectives	3	State specific objectives, including any prespecified hypotheses	The objectives are stated in the introduction on pages 5-6		
**Methods**					
Study Design	4	Present key elements of study design early in the paper	The study design is reported at the beginning of the methods section, under the subheading "study design and data source" on page 6		
Setting	5	Describe the setting, locations, and relevant dates, including periods of recruitment, exposure, follow-up, and data collection	The setting, locations, and relevant dates are described in the "study population and setting" section on pages 8-9		
Participants	6	*(a) Cohort study* - Give the eligibility criteria, and the sources and methods of selection of participants. Describe methods of follow-up *Case-control study* - Give the eligibility criteria, and the sources and methods of case ascertainment and control selection. Give the rationale for the choice of cases and controls	(a) Eligibility criteria, and the sources and methods of selection and follow-up of participants are given in the "study population and setting" section on pages 8-9 (b) N/A this was not a matched study	RECORD 6.1: The methods of study population selection (such as codes or algorithms used to identify subjects) should be listed in detail. If this is not possible, an explanation should be provided.<br><br>RECORD 6.2: Any validation studies of the codes or algorithms used to select the population should be referenced. If validation was conducted for this study and	6.1 We have described in detail the methods of study population selection in the "study population and setting" section on pages 8-9, and the "measures" section on page 10<br><br>6.2 We have referenced the validation studies of the anxiety and depression codes we have used in our study

For peer review only

		Cross-sectional study - Give the eligibility criteria, and the sources and methods of selection of participants<br><br>*(b) Cohort study* - For matched studies, give matching criteria and number of exposed and unexposed<br>*Case-control study* - For matched studies, give matching criteria and the number of controls per case		not published elsewhere, detailed methods and results should be provided.<br><br>RECORD 6.3: If the study involved linkage of databases, consider use of a flow diagram or other graphical display to demonstrate the data linkage process, including the number of individuals with linked data at each stage.	in the "measures" section on page 10<br><br>6.3 We have provided a flow diagram with the number of individuals included in each cohort at each stage of linkage (Figure 1), referred to on page 8
Variables	7	Clearly define all outcomes, exposures, predictors, potential confounders, and effect modifiers. Give diagnostic criteria, if applicable.	We have clearly defined all relevant measures in the "measures" section on page 10	RECORD 7.1: A complete list of codes and algorithms used to classify exposures, outcomes, confounders, and effect modifiers should be provided. If these cannot be reported, an explanation should be provided.	7.1 We have provided a list of codes used in the study in the appendix (pages 10-14 of the appendix)
Data sources/ measurement	8	For each variable of interest, give sources of data and details of methods of assessment (measurement). Describe comparability of assessment methods if	We have described all data sources and measures in the "study design and data sources" section on pages 8-9 and the "measures" section on page10		

For peer review only

		there is more than one group			
Bias	9	Describe any efforts to address potential sources of bias	We have explained that we used a conservative approach to identify cohort participants in the "study population and setting" section on page 8		
Study size	10	Explain how the study size was arrived at	We have included a flowchart (Figure 1) to describe the creation of each of the study cohorts and explained the process of cohort creation, referred to in the "study population and setting" section on page 8		
Quantitative variables	11	Explain how quantitative variables were handled in the analyses. If applicable, describe which groupings were chosen, and why	Handling of quantitative variables is described in the "measures" section on page 10		
Statistical methods	12	(a) Describe all statistical methods, including those used to control for confounding<br>(b) Describe any methods used to examine subgroups and interactions	(a) Statistical methods are described in the "statistical analysis" section on page 11<br>(b) N/A<br>(c) N/A<br>(d) N/A<br>(e) Sensitivity analyses are described in the		

For peer review only

		(c) Explain how missing data were addressed<br>(d) *Cohort study* - If applicable, explain how loss to follow-up was addressed<br>*Case-control study* - If applicable, explain how matching of cases and controls was addressed<br>*Cross-sectional study* - If applicable, describe analytical methods taking account of sampling strategy<br>(e) Describe any sensitivity analyses	appendix (pages 7-9 of the appendix)		
Data access and cleaning methods		..		RECORD 12.1: Authors should describe the extent to which the investigators had access to the database population used to create the study population.<br><br>RECORD 12.2: Authors should provide information on the data cleaning methods used in the study.	12.1 We described the extent to which the investigators had access to the database population in the "data access and cleaning methods" section on page 7<br>12.2 We provided information on the data cleaning methods in the "data access and cleaning methods" section on page 7

For peer review only

Linkage		..		RECORD 12.3: State whether the study included person-level, institutional-level, or other data linkage across two or more databases. The methods of linkage and methods of linkage quality evaluation should be provided.	12.3 The methods of linkage and quality evaluation are detailed in the "study design and data source" section on pages 6-7, the "study population and setting" section on pages 8-9, and the flowchart (Figure 1), referred to on page 8
**Results**					
Participants	13	(a) Report the numbers of individuals at each stage of the study (*e.g.*, numbers potentially eligible, examined for eligibility, confirmed eligible, included in the study, completing follow-up, and analysed)<br>(b) Give reasons for non-participation at each stage.<br>(c) Consider use of a flow diagram	(a) Numbers of individuals at each study stage are provided in the flow diagrams (Figure 1, referred to on page 8)<br>(b) Reasons for exclusion are provided on the flowchart (Figure 1, referred to on page 8)<br>(c) We used a flowchart to illustrate selection of our study cohorts (Figure 1, referred to on page 8)	RECORD 13.1: Describe in detail the selection of the persons included in the study (*i.e.,* study population selection) including filtering based on data quality, data availability and linkage. The selection of included persons can be described in the text and/or by means of the study flow diagram.	13.1 We described in detail the selection of the persons included in the study in the "study population and setting" section on pages 8-9 and Figure 1 referred to on page 8
Descriptive data	14	(a) Give characteristics of study participants (*e.g.*, demographic, clinical, social) and information	(a) Characteristics of study participants are given in Table 2, page 13<br>(b) N/A<br>(c) N/A		

For peer review only

		on exposures and potential confounders<br>(b) Indicate the number of participants with missing data for each variable of interest<br>(c) *Cohort study* - summarise follow-up time (*e.g.*, average and total amount)			
Outcome data	15	*Cohort study* - Report numbers of outcome events or summary measures over time<br>*Case-control study* - Report numbers in each exposure category, or summary measures of exposure<br>*Cross-sectional study* - Report numbers of outcome events or summary measures	Numbers of outcome events are reported in the results section in Table 4, pages 14-15		
Main results	16	(a) Give unadjusted estimates and, if applicable, confounder-adjusted estimates and their precision (e.g., 95% confidence interval). Make clear which confounders were	(a) We report unadjusted and adjusted estimates in the results section on pages 15-18, and have explained why we adjusted for each factor<br>(b) N/A<br>(c) N/A		

For peer review only

		adjusted for and why they were included<br>(b) Report category boundaries when continuous variables were categorized<br>(c) If relevant, consider translating estimates of relative risk into absolute risk for a meaningful time period			
Other analyses	17	Report other analyses done—e.g., analyses of subgroups and interactions, and sensitivity analyses	We report on sensitivity analyses in the appendix (pages 7-9 of the appendix)		
**Discussion**					
Key results	18	Summarise key results with reference to study objectives	Key results are summarised at the beginning of the discussion on page 21		
Limitations	19	Discuss limitations of the study, taking into account sources of potential bias or imprecision. Discuss both direction and magnitude of any potential bias	Limitations are discussed in the 'Strengths and limitations' section on page 23 including potential underestimation of our cohorts	RECORD 19.1: Discuss the implications of using data that were not created or collected to answer the specific research question(s). Include discussion of misclassification bias, unmeasured confounding, missing data, and changing eligibility over time, as they pertain to the study being reported.	We discussed the limitations of the Shielded Patient List in the 'Strengths and Limitations' section on page 23

For peer review only

Interpretation	20	Give a cautious overall interpretation of results considering objectives, limitations, multiplicity of analyses, results from similar studies, and other relevant evidence	We have given a cautious and balanced interpretation of the results with reference to the previous literature in the discussion on pages 21-22		
Generalisability	21	Discuss the generalisability (external validity) of the study results	Discussed in the "strengths and limitations" section on page 23		
**Other Information**					
Funding	22	Give the source of funding and the role of the funders for the present study and, if applicable, for the original study on which the present article is based	We have provided the funding source on page 25, and the role of the funders in the "Role of the funders" section on pages 7-8		
Accessibility of protocol, raw data, and programming code		..		RECORD 22.1: Authors should provide information on how to access any supplemental information such as the study protocol, raw data, or programming code.	We have provided information on how to access data within the SAIL databank on pages 26-27

*Reference: Benchimol EI, Smeeth L, Guttmann A, Harron K, Moher D, Petersen I, Sørensen HT, von Elm E, Langan SM, the RECORD Working Committee. The REporting of studies Conducted using Observational Routinely-collected health Data (RECORD) Statement. *PLoS Medicine* 2015; in press.

*Checklist is protected under Creative Commons Attribution (CC BY) license.

BMJ Open

Effects of the COVID-19 pandemic on the mental health of clinically extremely vulnerable children and children living with clinically extremely vulnerable people in Wales: a data linkage study

Journal:	*BMJ Open*
Manuscript ID	bmjopen-2022-067882.R2
Article Type:	Original research
Date Submitted by the Author:	01-Jun-2023
Complete List of Authors:	Cowley, Laura; Swansea University, Population Data Science<br>Hodgson, Karen; Public Health Wales, Research and Evaluation Division<br>Song, Jiao; Public Health Wales, Health Protection Division<br>Whiffen, Tony; Welsh Government, Administrative Data Research Unit<br>Tan, Jacinta; University of Oxford, Department of Psychiatry<br>John, Ann; Swansea University, Population Data Science<br>Bandyopadhyay, Amrita; Swansea University, National Centre for Population Health and Wellbeing Research<br>Davies, Alisha; Public Health Wales, Research and Evaluation Division
**Primary Subject Heading**:	Mental health
Secondary Subject Heading:	Public health, Health informatics, Health services research, Paediatrics
Keywords:	COVID-19, MENTAL HEALTH, Child & adolescent psychiatry < PSYCHIATRY, PUBLIC HEALTH, Depression & mood disorders < PSYCHIATRY, Anxiety disorders < PSYCHIATRY

SCHOLARONE™
Manuscripts

I, the Submitting Author has the right to grant and does grant on behalf of all authors of the Work (as defined in the below author licence), an exclusive licence and/or a non-exclusive licence for contributions from authors who are: i) UK Crown employees; ii) where BMJ has agreed a CC-BY licence shall apply, and/or iii) in accordance with the terms applicable for US Federal Government officers or employees acting as part of their official duties; on a worldwide, perpetual, irrevocable, royalty-free basis to BMJ Publishing Group Ltd ("BMJ") its licensees and where the relevant Journal is co-owned by BMJ to the co-owners of the Journal, to publish the Work in this journal and any other BMJ products and to exploit all rights, as set out in our licence.

The Submitting Author accepts and understands that any supply made under these terms is made by BMJ to the Submitting Author unless you are acting as an employee on behalf of your employer or a postgraduate student of an affiliated institution which is paying any applicable article publishing charge ("APC") for Open Access articles. Where the Submitting Author wishes to make the Work available on an Open Access basis (and intends to pay the relevant APC), the terms of reuse of such Open Access shall be governed by a Creative Commons licence – details of these licences and which Creative Commons licence will apply to this Work are set out in our licence referred to above.

Other than as permitted in any relevant BMJ Author's Self Archiving Policies, I confirm this Work has not been accepted for publication elsewhere, is not being considered for publication elsewhere and does not duplicate material already published. I confirm all authors consent to publication of this Work and authorise the granting of this licence.

For peer review only

Effects of the COVID-19 pandemic on the mental health of clinically extremely vulnerable children and children living with clinically extremely vulnerable people in Wales: a data linkage study

Laura E Cowley[1], PhD, Karen Hodgson[2], PhD, Jiao Song[3], PhD, Tony Whiffen[4], MSc, Jacinta Tan[5], PhD, Prof Ann John[1], PhD, Amrita Bandyopadhyay[6], MSc, Prof Alisha R Davies[2,6], PhD

Affiliations:
[1] Population Data Science, Swansea University Medical School, Wales, United Kingdom
[2] Research and Evaluation Division, Public Health Data, Knowledge and Research Directorate, Public Health Wales, Cardiff, Wales, United Kingdom
[3] Communicable Disease Surveillance Centre, Health Protection Division, Public Health Wales, Cardiff, Wales, United Kingdom
[4] Administrative Data Research Unit, Welsh Government, Wales, United Kingdom
[5] Department of Psychiatry, University of Oxford
[6] National Centre for Population Health and Wellbeing Research, Swansea University Medical School, Wales, United Kingdom

Correspondence to: Dr Laura Cowley, Data Science Building, Singleton Park, Swansea, SA2 8PP, Wales, United Kingdom. E-mail: l.e.cowley@swansea.ac.uk

Author email addresses: l.e.cowley@swansea.ac.uk; Karen.Hodgson@wales.nhs.uk; Jiao.Song@wales.nhs.uk; Anthony.Whiffen@gov.wales; Jacinta.Tan@oxfordhealth.nhs.uk; A.John@swansea.ac.uk; A.Bandyopadhyay@swansea.ac.uk; Alisha.davies@wales.nhs.uk

Word count: 3,981

For peer review only

Abstract

Objectives: To determine whether clinically extremely vulnerable (CEV) children or children living with a CEV person in Wales were at greater risk of presenting with anxiety or depression in primary or secondary care during the COVID-19 pandemic compared with children in the general population, and to compare patterns of anxiety and depression during the pandemic (23rd March 2020–31st January 2021, referred to as 2020/21) and before the pandemic (March 23rd 2019–January 31st 2020, referred to as 2019/20), between CEV children and the general population.

Design: Population-based cross-sectional cohort study using anonymised, linked, routinely collected health and administrative data held in the Secure Anonymised Information Linkage Databank. CEV individuals were identified using the COVID-19 Shielded Patient List.

Setting: Primary and secondary healthcare settings covering 80% of the population of Wales.

Participants: Children aged 2–17 in Wales: CEV (3,769); living with a CEV person (20,033); or neither (415,009).

Primary outcome measure: First record of anxiety or depression in primary or secondary healthcare in 2019/20 and 2020/21, identified using Read and ICD-10 codes.

Results: A Cox regression model adjusted for demographics and history of anxiety or depression revealed that only CEV children were at greater risk of presenting with anxiety or depression during the pandemic compared with the general population (Hazard Ratio=2.27, 95% Confidence Interval=1.94–2.66, p<0.001). Compared with the general population, the risk amongst CEV children was higher in 2020/21 (Risk Ratio 3.04) compared with 2019/20 (Risk Ratio 1.90). In 2020/21, the period prevalence of anxiety or depression increased slightly amongst CEV children, but declined amongst the general population.

Conclusions: Differences in the period prevalence of recorded anxiety or depression in healthcare between CEV children and the general population were largely driven by a reduction in presentations to healthcare services by children in the general population during the pandemic.

Strengths and limitations of this study

- Strengths of this study include its national focus and clinical relevance; to our knowledge, this is the first population-based study examining the effects of the COVID-19 pandemic on healthcare use for anxiety or depression amongst clinically extremely vulnerable (CEV) children and children living with a CEV person in Wales.
- We compared 2020/21 data with pre-pandemic 2019/20 data for CEV children and children in the general population, to place the impact of the COVID-19 pandemic in the context of longer-term patterns of healthcare use.
- We used a novel approach and linked multiple datasets to identify a cohort of children living with a CEV person in Wales during the COVID-19 pandemic.
- There was heterogeneity within the Shielded Patient List that was used to create the cohorts of children identified as CEV or living with a CEV person, in terms of the type and severity of individuals' underlying conditions; the manner in which people

For peer review only - http://bmjopen.bmj.com/site/about/guidelines.xhtml

were added to the list; the time point that people were added to the list; and the extent to which people followed the shielding guidance.

- Routinely collected healthcare data does not capture self-reported health, and is likely to underestimate the burden of common mental disorders in the population.

Keywords: COVID-19, mental health, child and adolescent psychiatry, public health, depression and mood disorders, anxiety disorders

For peer review only

Introduction

In March 2020, Welsh Government and the Welsh National Health Service sought to protect people deemed clinically extremely vulnerable (CEV) to severe illness or death from COVID-19, advising them to 'shield' at home i.e. to remain indoors and minimise contact with others.[1] To identify CEV people, a Shielded Patient List (SPL) was created, using an algorithm based on clinical code lists and applied centrally to patients' electronic health records.[2] Additionally, health professionals could add people to the list based on their clinical judgement. Shielding was in place from 23rd March 2020 until 16th August 2020 and re-introduced from 22nd December 2020 until 1st April 2021.[1] CEV children were encouraged to return to school at the end of August 2020 taking into account the low rate of severe disease and death from COVID-19 amongst children, balanced against the harms of a lack of schooling and socialization.[3]

Studies have highlighted the detrimental impact of the COVID-19 pandemic on the mental health of CEV adults, with CEV individuals more likely to report increased depressive symptoms and anxiety[4] and to have a clinical record of anxiety or depression during the pandemic compared with those who were not CEV.[1] Meanwhile, studies have reported decreased diagnoses of mental health conditions in primary care across the population as a whole during the pandemic[5,6] (attributed to a reluctance to seek healthcare, or reduced access to services, rather than a decrease in need). However, there is limited evidence on how children accessed healthcare during the pandemic for their mental health, and no studies focusing on CEV children, or children living with a CEV person.

Children are particularly vulnerable to indirect impacts of the pandemic.[7] Drawing on evidence from longitudinal surveys,[8–10] the Department for Education concluded that

For peer review only - http://bmjopen.bmj.com/site/about/guidelines.xhtml

children's mental health declined during the pandemic, reporting that rates of probable mental health disorders were higher from 2020 than before.[11] The data also highlighted variation in mental health trajectories; children with long-term health conditions were more likely to experience poor mental health during the pandemic.[11] However, the extent to which these trends are attributable to the pandemic, or are a continuation of pre-existing upward trends is unclear.

Almost 5,000 CEV children were living in Wales by July 2020; approximately 3.9% of the Welsh CEV population.[12] Children with chronic illnesses are at increased risk of behavioural and emotional problems and psychiatric disorders compared with their peers,[13] but CEV children may be particularly susceptible to mental health difficulties relative to non-CEV children since the pandemic, due to additional restrictions imposed by shielding guidance, potentially exacerbating loneliness and isolation.[14] CEV children may have also experienced heightened health anxiety due to their potential higher risk of severe illness from COVID-19. Additionally, there were almost 14,400 school-aged children living with a CEV person in June 2020[15] who may be at greater risk of mental health difficulties due to both restrictions to protect the vulnerable members of their household, and fears of causing harm.[16]

We investigated the impact of the COVID-19 pandemic on use of healthcare services for anxiety or depression in Wales, for CEV children, children living with a CEV person, and children in the general population, using routinely collected population-level linked data. The primary aim was to determine whether CEV children or children living with a CEV person were more likely to have a record for anxiety or depression in primary or secondary care during the pandemic compared with children in the general population. The secondary aim was to compare patterns of anxiety or depression in 2019/2020 and 2020/2021 between CEV

For peer review only - http://bmjopen.bmj.com/site/about/guidelines.xhtml

children and the general population, to place the impact of COVID-19 and the shielding guidance in the context of longer-term patterns of healthcare use.

Methods

Study design and data sources

This is a population-based cross-sectional cohort study using anonymised, linked, routinely collected health and administrative data for the population of Wales, UK, held in the Secure Anonymised Information Linkage (SAIL) databank (www.saildatabank.com). Within the SAIL databank, encrypted linkage fields are used to link data anonymously from various sources at individual and household level (appendix pp 1–2); known as Anonymised Linking Fields (ALFs) for individuals and Residential Anonymised Linking Fields (RALFs) for residences.[17,18] We used these to link multiple datasets in this study (Table 1). General Practices (GPs) opt-in to providing data to SAIL; currently, SAIL contains primary care data for around 80% of the Welsh population, and the available data are representative of the entire Welsh population with respect to age, sex, and deprivation.[19] The SAIL databank was interrogated using DB2 structured query language (SQL).

Table 1. Datasets used in this study

Dataset	Description
Welsh Demographic Service (WDS) dataset	A register containing demographic information about all individuals registered at a Welsh General Practice (GP)
COVID-19 Shielded People List (CVSP)	A dataset containing information about clinically extremely vulnerable individuals in Wales, including reasons for shielding
Welsh Index of Multiple Deprivation 2019 (WIMD 2019)	A dataset containing deprivation scores corresponding to all Lower Super Output Areas (LSOAs; geographic units comprised of around 1,600 individuals) in Wales[24]
Rural Urban Classification (RUC) dataset	A dataset containing information on urban/rural categories corresponding to all LSOAs in Wales
Annual District Death Extract (ADDE)	A register containing details of all deaths of Welsh residents, including information regarding date and cause of death

For peer review only

Outpatient Dataset (OPD)	A dataset containing attendance information for all hospital outpatient appointments in Wales
National Community Child Health Database (NCCHD)	A register of children born in Wales, containing data collected at birth, including a maternal ALF to link children with their biological mothers
Patient Episode Database for Wales (PEDW)	A dataset containing attendance and clinical information for all hospital admissions in Wales, including data regarding diagnoses
Care Homes Data (CHD)	A dataset containing residential information about adult care homes in Wales
Welsh Longitudinal General Practice (WLGP) dataset	A dataset containing attendance and clinical information for all GP interactions including symptoms, diagnoses, and prescriptions

For peer review only

Ethical approval

This study used anonymised data and therefore did not require National Research Ethics Committee approval. Approval to access and link the data within SAIL was granted by the Information Governance Review Panel under project number 1265 (appendix p 3).

Patient and public involvement

The study protocol was presented at the SAIL consumer panel meeting prior to study commencement. This panel consists of members of the public with an interest in data and its uses to improve services and healthcare. The panel provided advice and feedback on the study design from a public perspective.

Data access and cleaning methods

All authors had full access to all the data in the study. Data cleaning included deduplication and restructuring of the SPL prior to cohort creation and analysis, and was undertaken by LEC.

Study population and setting

For peer review only - http://bmjopen.bmj.com/site/about/guidelines.xhtml

We created three study cohorts for 2020: 1) CEV children 2) children living with at least one CEV person and 3) a general population group of children who were *not* identified as CEV or living with a CEV person, along with two further cohorts for 2019 for comparison purposes. Children who were both CEV and living with a CEV person were categorised as CEV. Figure 1 shows a flow diagram of the inclusion criteria for each cohort. We included all children aged 2–17 years who were alive, living in Wales and registered with a SAIL-supplying GP on 23rd March 2020; and who had either an exact match on NHS number or demographics (name, date of birth, gender code, and postcode), or a probabilistic match of 90% or greater.[17] We excluded those for whom full demographic or residence data were not available.

Cohort 1: Clinically extremely vulnerable children

Cohort 1 consisted of all children who were identified as CEV (either by algorithm[2] or health professionals) between 23rd March 2020 and 16th August 2020 (N=3,769).

Cohort 2: Children living with at least one clinically extremely vulnerable person

To identify children living with a CEV person, we first identified the RALF for all CEV people in Wales as of 23rd March 2020, including dates of residence. To minimize bias, we then adopted a conservative approach and included 1) children who were recorded as residents at the same RALF on 23rd March 2020 and had an entry date of residence within 6 months of the entry date of the CEV person 2) children born to mothers who were recorded as resident at the same RALF as the CEV person, or 3) children who shared the same maternal ALF as a younger CEV child. We excluded 1) adult care homes as these were unlikely to contain children[20] and 2) RALFs containing more than ten people, as the Unique Property Reference Number (UPRN) from which the RALF is derived, is considered inaccurate in this case[21], with a final 20,033 children in cohort 2.

For peer review only - http://bmjopen.bmj.com/site/about/guidelines.xhtml

Cohort 3: Children in the general population

Cohort 3 consisted of all other children who were alive, living in Wales and registered with a SAIL-supplying GP on 23rd March 2020 (N=415,009).

Clinically extremely vulnerable children in Wales in 2019 (pre-COVID-19 CEV children)

To explore longer-term patterns of anxiety or depression amongst children with the conditions included within the shielding guidance, we created a cohort of pre-COVID-19 "CEV" children who had similar health concerns to CEV children in a period prior to 2020. Creation of this pre-pandemic cohort enabled us to explore whether adverse mental health outcomes for CEV children during the pandemic were likely attributable to the shielding guidance, or whether CEV children experience poorer mental health compared with the general population regardless of having to shield. We included children who were alive, living in Wales, and registered with a SAIL-supplying GP one year prior to the introduction of shielding (23rd March 2019) and who had one or more of three of the health condition categories warranting inclusion on the SPL (respiratory illnesses, blood or bone cancer, and immunosuppression therapy). These categories were chosen as they only required the patient to have one of the listed codes within a given time period and therefore children with these conditions could be identified with a high degree of certainty. We used International Classification of Diseases (ICD) version 10 diagnostic codes and OPCS Classification of Interventions and Procedures version 4 codes for these categories, which were taken from the SPL documentation and provided in the appendix (pp 4–5). For comparison purposes we also created a general population cohort of children aged 2–17 who were alive, living in Wales, and registered with a SAIL-supplying GP as of 23rd March 2019. A flow diagram of inclusion

For peer review only - http://bmjopen.bmj.com/site/about/guidelines.xhtml

criteria for these cohorts is provided in the appendix (p 6). We performed a sensitivity analysis to confirm the validity of this approach (appendix pp 7–9).

Measures

Outcome of interest: Risk of anxiety or depression

The outcome of interest was the first record of anxiety or depression in primary or secondary healthcare data during the COVID-19 pandemic (i.e. 23rd March 2020–31st January 2021, referred to as 2020/21), and pre-pandemic (March 23rd 2019–January 31st 2020, referred to as 2019/20). We included any healthcare visit where anxiety or depression was documented in the electronic health record. We used validated Read v2 codes to identify children with primary care records for anxiety[22] or depression[23] (including diagnoses, symptoms, and prescriptions) in the WLGP dataset. Read codes are a hierarchical terminology system that encode clinical, diagnostic and therapeutic patient information, and enable data entry of patient care information following a primary care consultation. Separate Read codes are used to record a patients' reported past medical history, or to note that a clinician is aware of a past condition. We used ICD-10 diagnostic codes to identify children with hospital admissions or outpatient appointments for anxiety or depression in the PEDW and OPD datasets. If children had multiple records of anxiety or depression during the relevant time periods, we sequenced these and selected the record with the earliest date. Code lists were reviewed by a clinician with expertise in child psychiatry (JT) and are provided in the appendix (pp 10–14). The primary outcome was the *time to the first record* of anxiety or depression for the children in each cohort, and the secondary outcome was the *period prevalence* of anxiety or depression for the children in each cohort.

Covariates: History of anxiety or depression

For peer review only - http://bmjopen.bmj.com/site/about/guidelines.xhtml

We used the same process to identify children in the study population with a ‘recent’ or ‘past’ history of anxiety or depression, defined as any record in the year prior to the pandemic (23rd March 2019–23rd March 2020), and any record occurring any time before 23rd March 2019 respectively.[1]

Covariates: Demographics

We calculated age and determined Lower-layer Super Output Areas (LSOA) as of 23rd March 2020. LSOA codes were derived from the WDS dataset based on the child’s RALF, and used to ascertain deprivation quintiles and urban/rural classification by linking to the WIMD 2019[24] and RUC datasets.

Statistical analysis

We used R version 4.1.1 for statistical analyses. P-values of <0.05 were considered statistically significant.

Examining risk of anxiety or depression between the different cohorts in 2020/21

We tested the hypothesis that there was no difference in the risk of having a record of anxiety or depression in 2020/21 between the three cohorts (CEV children, children living with a CEV person, and children in the general population). We plotted Kaplan-Meier survival curves for each cohort. We used Cox regression to calculate unadjusted and adjusted hazard ratios (HRs) with 95% confidence intervals (CI). We report three models examining the risk of having a record of anxiety or depression during the pandemic compared with the general population; (1) unadjusted, (2) adjusted for demographic factors (age group, sex, deprivation and rurality) and (3) adjusted for demographic factors and previous history of anxiety or depression (no history, recent history, past history, or both recent and past history).

For peer review only - http://bmjopen.bmj.com/site/about/guidelines.xhtml

Examining risk of anxiety or depression between the different cohorts in 2019/20

We tested the hypothesis that there was no difference in the risk of having a record of anxiety or depression in 2019/20 between two cohorts ("CEV" children and all other children living in Wales in 2019). As above, we calculated unadjusted and adjusted HRs, reporting three models, and plotted Kaplan-Meier survival curves for each cohort.

Comparing the risk of anxiety or depression in children between 2019/20 and 2020/21

We calculated the change in the period prevalence of anxiety or depression for CEV or "CEV" children, and all other children living in Wales, between 2019 and 2020.

Study reporting

This study is reported in accordance with the Reporting of Studies conducted using Observational Routinely-collected data (RECORD) guidelines[25] (appendix pp 15–23).

Results

Descriptive statistics and demographic characteristics of the study population

Demographic characteristics of the 2020/21 study population are presented in Table 2, and for the 2019/20 study population in the appendix (p 24). For both years, there were greater proportions of boys, older children (aged 13–17), children living in the least and most deprived quintiles and children with a history of anxiety or depression in the CEV children than for the general population. In 2020/21, a greater proportion of children living with a CEV person were older (aged 13–17), and had a history of anxiety or depression, compared with the general population (Table 2). The conditions leading to children being identified as CEV in 2020/21 are shown in Table 3.

For peer review only - http://bmjopen.bmj.com/site/about/guidelines.xhtml

Table 2. Characteristics of clinically extremely vulnerable (CEV) children, children living with a CEV person, and a general population group of children neither CEV or living with a CEV person, 2020/21, Wales

		General population	Clinically extremely vulnerable children	χ^2 statistic, degrees of freedom, and p value, general population v. clinically extremely vulnerable children	Children living with a clinically extremely vulnerable person	χ^2 statistic, degrees of freedom, and p value, general population v. children living with a clinically extremely vulnerable person
N		415,009	3,769		20,033	
**Sex (%)**	Male	212,311 (51·2)	2,184 (57·9)	χ^2=68·6, df=1, p<0·001	10,247 (51·2)	χ^2=0·0, df=1, p=0·989
	Female	202,698 (48·8)	1,585 (42·1)		9,786 (48·8)	
**Age group (years) (%)**	2–7	150,999 (36·4)	1,141 (30·3)	χ^2=84·0, df=2, p<0·001	6,692 (33·4)	χ^2=93·7, df=2, p<0·001
	8–12	137,310 (33·1)	1,247 (33·1)		6,678 (33·3)	
	13–17	126,700 (30·5)	1,381 (36·6)		6,663 (33·3)	
**Deprivation quintile (WIMD 2019) (%)**	1	106,075 (25·6)	1,014 (26·9)	χ^2=11·1, df=4, p=0·025	5,058 (25·2)	χ^2=10·8, df=4, p=0·029
	2	87,930 (21·2)	773 (20·5)		4,107 (20·5)	
	3	73,106 (17·6)	635 (16·8)		3,611 (18·0)	
	4	69,789 (16·8)	589 (15·6)		3,488 (17·4)	
	5	78,109 (18·8)	758 (20·1)		3,769 (18·8)	
**Rural/Urban area (%)**	Rural	110,682 (26·7)	971 (25·8)	χ^2=1·5, df=1, p=0·217	5,444 (27·2)	χ^2=2·5, df=1, p=0·116
	Urban	304,327 (73·3)	2,798 (74·2)		14,589 (72·8)	
**Any history of anxiety or depression (%)**	YES	17,986 (4·3)	368 (9·8)	χ^2=261·5, df=1, p<0·001	1,131 (5·6)	χ^2=78·0, df=1, p<0·001
	NO	397,023 (95·7)	3,401 (90·2)		18,902 (94·4)	

For peer review only - http://bmjopen.bmj.com/site/about/guidelines.xhtml

Table 3. Proportion of clinically extremely vulnerable children with different conditions contributing to underlying reasons to shield

Reason for shielding	Number and percentage of clinically extremely vulnerable children (n = 3,769)
Rare diseases	1,227 (32·6%)
Organ disease	735 (19·5%)
Respiratory illness	660 (17·5%)
Immunosuppression therapy	491 (13%)
Cancer	147 (3·9%)
Transplant	132 (3·5%)
Renal dialysis	10 (0·3%)
GP referred *(reason unknown)*	582 (15·4%)
Other *(reason unknown)*	22 (0·6%)

In children whose reasons for shielding were known (3,165/3,769) 6.3% (198/3,165) had more than one condition.

Risk of anxiety or depression in children in 2020/21

Numbers of censored children in each group (due to death, migration or registration with a non-SAIL supplying GP) and numbers with a record for anxiety or depression are presented in Table 4. Of those with a record, 5,768/6,251 (92.3%) presented to primary care while 483/6,251 (7.7%) presented to secondary care.

Table 4. Number of children who were censored, with no event, or who had a record of anxiety or depression during 2020/21

	General population	Clinically extremely vulnerable children	Children living with a clinically extremely vulnerable person
N	415009	3769	20033
Died (%)	12 (0·003)	6 (0·2)	0 (0·0)
Moved out of Wales (%)	7297 (1·8)	68 (1·8)	245 (1·2)
Moved to a non SAIL-supplying GP (%)	5664 (1·4)	31 (0·8)	262 (1·3)
No event (%)	396267 (95·5)	3505 (93·0)	19203 (95·9)

For peer review only - http://bmjopen.bmj.com/site/about/guidelines.xhtml

Anxiety or depression during the pandemic 2020/21 (%)	5,769 (1·4)	159 (4·2)	323 (1·6)

In the unadjusted model, both CEV children and children living with a CEV person were at significantly greater risk of having a record of anxiety or depression during the pandemic compared with the general population (HR=3·09, 95% CI=2·64–3·61, p<0·001 and HR=1·16, 95% CI=1·04–1·30, p<0·05, respectively). For CEV children, the hazard ratio remained significant when adjusting for demographic factors including age, sex, deprivation and rurality (Table 5), and when adjusting for demographic factors and previous clinical history of anxiety or depression (Table 6). However, for children living with a CEV person, the hazard ratio was no longer significant in either of the adjusted models. The unadjusted survival curves for each cohort are shown in Figure 2.

For peer review only - http://bmjopen.bmj.com/site/about/guidelines.xhtml

Table 5. Multivariable analysis of risk factors for having a record of anxiety or depression during the COVID-19 pandemic (23rd March 2020–31st January 2021), reported using hazard ratios and 95% confidence intervals (model adjusting for demographic factors only)

		Hazard ratio	95% confidence interval	P value
**Cohort**	General population	Reference group		
	Clinically extremely vulnerable (CEV) children	2·81	2.40–3.29	<·001
	Children living with a CEV person	1.09	0·97–1·22	0·14
**Sex**	Male	Reference group		
	Female	1·94	1·84–2.04	<·001
**Age group**	2–7	Reference group		
	8–12	5.21	4·58–5·92	<·001
	13–17	19·39	17.20–21.86	<·001
**Deprivation quintile (WIMD 2019)**	1 (most deprived)	1·13	0·89–1·05	<0.01
	2	1.05	0·95–0.97	0·19
	3	1·05	0·95–0.97	0·23
	4	1·10	0·91–1·01	<0.05
	5 (least deprived)	Reference group		
**Rural/Urban area**	Urban	Reference group		
	Rural	1·02	0·98–0.97	0·45

Table 6. Multivariable analysis of risk factors for having a record of anxiety or depression during the COVID-19 pandemic (23rd March 2020–31st January 2021), reported using hazard ratios and 95% confidence intervals (model adjusting for both demographic and mental health factors)

For peer review only - http://bmjopen.bmj.com/site/about/guidelines.xhtml

		Hazard ratio	95% confidence interval	P value
**Cohort**	General population	Reference group		
	Clinically extremely vulnerable (CEV) children	2·27	1·94–2·66	<·001
	Children living with a CEV person	1·02	0·91–1·14	0·746
**Sex**	Male	Reference group		
	Female	1·58	1·50–1·66	<·001
**Age group**	2–7	Reference group		
	8–12	4·56	4·01–5·18	<·001
	13–17	11·05	9·78–12·49	<·001
**Deprivation quintile (WIMD 2019)**	1 (most deprived)	1·02	0·95–1·10	0·565
	2	0·98	0·90–1·06	0·614
	3	1·00	0·92–1·09	0·965
	4	1·07	0·98–1·16	0·127
	5 (least deprived)	Reference group		
**Rural/Urban area**	Urban	Reference group		
	Rural	1·02	0·96–1·02	0·464
**History of anxiety or depression**	No history	Reference group		
	Past history only	5·13	4·75–5·53	<·001
	Recent history only	8·75	8·12–9·44	<·001
	Both recent and past history	18·98	17·52–20·55	<·001

For peer review only - http://bmjopen.bmj.com/site/about/guidelines.xhtml

Risk of anxiety or depression in children in 2019/2020

In 2019/20, in the unadjusted model there was an increased risk of having a record of anxiety or depression amongst the "CEV" children compared with the general population (HR=1·94, 95% CI=1·31–2·87, p<0·001). This remained evident in the adjusted models (Table 7 and Table 8). The unadjusted survival curves for each cohort are shown in Figure 3.

For peer review only

For peer review only - http://bmjopen.bmj.com/site/about/guidelines.xhtml

Table 7. Multivariable analysis of risk factors for having a record of anxiety or depression between March 23rd 2019–January 31st 2020, reported using hazard ratios and 95% confidence intervals (model adjusting for demographic factors only)

		Hazard ratio	95% confidence interval	P value
**Cohort**	General population	Reference group		
	Clinically extremely vulnerable (CEV) children	2·03	1.37–3.01	<·001
**Sex**	Male	Reference group		
	Female	1·85	1·77–1·93	<·001
**Age group**	2–7	Reference group		
	8–12	4·60	4.17–5.08	<·001
	13–17	18·50	16.89–20.27	<·001
**Deprivation quintile (WIMD 2019)**	1 (most deprived)	1·32	1.25–1·41	<·001
	2	1.22	1.15–1·31	<·001
	3	1·13	1.06–1·22	<·001
	4	1·09	1.02–1·17	<0.05
	5 (least deprived)	Reference group		
**Rural/Urban area**	Urban	Reference group		
	Rural	0.97	0·93–1·02	0·21

Table 8. Multivariable analysis of risk factors for having a record of anxiety or depression between March 23rd 2019–January 31st 2020, reported using hazard ratios and 95% confidence intervals (model adjusting for both demographic and mental health factors)

For peer review only - http://bmjopen.bmj.com/site/about/guidelines.xhtml

		Hazard ratio	95% confidence interval	P value
**Cohort**	General population	Reference group		
	Clinically extremely vulnerable (CEV) children	2·03	1.37–3.01	<·001
**Sex**	Male	Reference group		
	Female	1·54	1·48–1·61	<·001
**Age group**	2–7	Reference group		
	8–12	4·13	3.74–4.56	<·001
	13–17	11·56	10.53–12.68	<·001
**Deprivation quintile (WIMD 2019)**	1 (most deprived)	1·23	1.15–1·30	<·001
	2	1.14	1.07–1·22	<·001
	3	1·09	1.02–1·16	<0·05
	4	1·05	0·98–1·13	0·17
	5 (least deprived)	Reference group		
**Rural/Urban area**	Urban	Reference group		
	Rural	0.97	0·92–1·02	0·19
**History of anxiety or depression**	No history	Reference group		
	Past history only	4.50	4·21–4.81	<·001
	Recent history only	8·44	7.94–8.96	<·001
	Both recent and past history	16.97	15.85–18.18	<·001

For peer review only

Difference in the risk of records of anxiety or depression between 2019/20 and 2020/21

In 2019/20, "CEV" children had increased risk of having recorded anxiety or depression compared with children in the general population, and in 2020/21 the risk ratio increased to 3.04 (Table 9). This reflects a marked decline in presentation amongst children in the general population over this period (from 2·19% to 1·39%), alongside a small increase for CEV children (from 4·17% to 4·22%).

For peer review only

For peer review only - http://bmjopen.bmj.com/site/about/guidelines.xhtml

Table 9. Risk of records of anxiety or depression in children (aged 2–17 years) who were clinically extremely vulnerable (CEV) and those in the general population, in 2019/20 and 2020/21

**Time period**	**CEV children**<br>*2020/21 - children identified through shielded patient list*<br>*2019/20 – comparable cohort of children with the conditions listed in the shielded patient list*			**Children in the general population in Wales**			**Crude Risk Ratio**
	**No. children with recorded anxiety or depression**	**Total No. of children**	**Period Prevalence (%)**	**No. children with recorded anxiety or depression**	**Total No. of children**	**Period Prevalence (%)**	
2019/20 (Pre COVID-19)	25	599	4·17	9,620	438,924	2·19	1·90
2020/21 (During COVID-19)	159	3,769	4·22	5,769	415,009	1·39	3·04
**Percentage point change over time points**			-0·05			0·8	

For peer review only - http://bmjopen.bmj.com/site/about/guidelines.xhtml

Discussion

In Wales, CEV children and children living with a CEV person were more likely to access health services for anxiety or depression during the pandemic than children in the general population. For CEV children, this pattern remained evident after adjusting for demographic differences and the likelihood of having a previous history of anxiety or depression. Although a small increase in risk was found for children living with a CEV person, after adjusting for demographic characteristics and previous history of anxiety and depression, this was no longer significant.

Both before and during the pandemic, the groups with the greatest risk of having a record for anxiety or depression were adolescents aged 13–17, and those with both a past and recent history of anxiety or depression. Females were also more likely than males to have a record for anxiety or depression in both time periods. These findings are in line with previous studies reporting worse mental health in female adolescents compared with males[26]; a higher prevalence of anxiety and depression in adolescents and females compared with younger children and males during the pandemic[27]; and worse mental health outcomes during the pandemic for those with pre-existing mental health difficulties[28]. This suggests that moving forward, it will be important to prioritise mental health support for female adolescents, and particularly for children who have concomitant physical and mental health conditions.

Given the detailed methodology used to identify CEV individuals in Wales,[2] we were able to develop a comparable cohort of children with a subset of health conditions before the pandemic, using routine healthcare data. This enabled us to examine patterns of presentation for anxiety and depression amongst CEV children outside of the context of the pandemic. We found this group were at greater risk of having a record for anxiety or depression compared

For peer review only - http://bmjopen.bmj.com/site/about/guidelines.xhtml

with children in the general population in 2019/20, before COVID-19. In 2020/21, CEV children remained at higher risk, and the difference was greater, although this is explained by a marked decline amongst children in the general population presenting to healthcare services with anxiety or depression during this time.

The reduction in presentations for anxiety and depression amongst children in the general population most likely reflects reduced access to NHS services during the pandemic. Other evidence suggests increased demand and unmet need for mental health support in the UK, for children with and without pre-existing mental health problems, since 2020.[29,30] Findings from self-report surveys[8–10] and the current study suggest that the pandemic has widened the gap between need and access to mental healthcare for the general population of children in Wales, but additional data are required to unpack the relationship between self-reported mental health needs and presentation to healthcare services.

Meanwhile, the relatively stable period prevalence of anxiety and depression for CEV children in 2019/20 and 2020/21 could indicate that this group did not experience an increase in mental health needs during the pandemic over and above past years, and that they had access to mental health support through existing care pathways for underlying conditions. Alternatively, if CEV children experienced the same increase in mental health needs as reported elsewhere amongst the general population, then these figures may mask unmet demand for mental health support among this group. A survey of adults supports the latter explanation, reporting increased anxiety and depressive symptoms amongst shielding individuals compared with non-shielding individuals.[4] However, we have found no UK surveys focusing on the mental health of CEV children.

For peer review only - http://bmjopen.bmj.com/site/about/guidelines.xhtml

Our finding that children living with a CEV person were at no greater risk of presenting with anxiety or depression during the pandemic compared with the general population (after controlling for other factors), could be interpreted in two ways. It is possible that the impact of the pandemic on the mental health of children living with a CEV person was not as great as for CEV children, but this seems unlikely given that research has suggested increased anxiety amongst children who were shielding their siblings.[16] Another explanation is that children living with a CEV person suffered from similar barriers to access to mental healthcare services to the general population, and did not have the same routes to access that CEV children did. This explanation is supported by research with shielding families, which suggests that they have felt left behind, and that children living with a CEV person may have 'fallen under the radar of educational and healthcare professionals'.[16]

Strengths and limitations

To our knowledge, this is the first population-based study examining the effects of the COVID-19 pandemic on healthcare use for anxiety or depression amongst CEV children and children living with a CEV person in Wales. Linkage of population-based routinely collected data is a valuable method for generating evidence with a high level of external validity and applicability for policy making. A strength of this study is the comparison of 2020/21 data with pre-pandemic 2019/20 data for CEV children and children in the general population. Another strength is the use of a novel approach using multiple linked datasets to identify a cohort of children living with a CEV person during the pandemic.

This study used the SPL to create cohorts of children identified as CEV and a cohort of children living with a CEV person. There was heterogeneity within the SPL in terms of the type and severity of individuals' underlying conditions; the manner in which people were

For peer review only - http://bmjopen.bmj.com/site/about/guidelines.xhtml

added to the list (via the algorithm or clinical judgement); the time point that people were added to the list; and the extent to which people followed the shielding guidance. In addition, the impact of following shielding guidance is likely to have varied due to individual circumstances and the level of support received. The 2019 "CEV" cohort was a relatively small sample and for pragmatic reasons, only included children with a subset of the conditions included in the shielding guidance. To identify children living with a CEV person, we adopted a conservative approach and included children only if they were living with their mother. We took this approach in order to minimize bias and increase the generalisability of the findings, however this approach is likely to have underestimated the number of children living with a CEV person. Finally, this study focused on healthcare use using clinical codes. Routinely collected healthcare data does not capture self-reported health, and is likely to underestimate the burden of common mental disorders in the population.[31] Focusing on healthcare use with routine data alone cannot tell us about the underlying reasons for changes in utilisation, or the scale of mental health need.

Implications for policy and practice

Our findings have implications for recovery planning to prevent, mitigate and respond to the mental health impacts of the pandemic. We have shown changes in presentation to primary and secondary healthcare services with anxiety and depression for CEV children and children in the general population during the pandemic, and there are concerns regarding potential increases in unmet mental health needs over time. As highlighted by UK organisations such as the Centre for Mental Health,[32] services face challenges in tackling this demand. This has been recognised by Welsh Government, who invested an additional £9.4m in children's mental health services in 2021.[33]

For peer review only - http://bmjopen.bmj.com/site/about/guidelines.xhtml

This novel linked data study contributes to our understanding of the direct and indirect impact of shielding on children's mental health in Wales during the COVID-19 pandemic. This evidence should be considered in light of additional, more detailed routine healthcare linkage studies, and national surveys, to provide a comprehensive understanding of the relationship between mental health support needs, expressed demands and care provision to better target services to those who need them the most.

Beyond the indirect impacts of the pandemic, our findings highlight the increased mental health needs of children with serious medical conditions. Given that these children are likely to have greater contact with healthcare services, signposting across services including mental health services, is likely to be beneficial.

*** ***

Funding

This work was supported by the Adolescent Mental Health Data Platform (ADP). The ADP is funded by MQ Mental Health Research Charity (Grant Reference MQBF/3 ADP). This work was also supported by the National Centre for Population Health and Wellbeing Research (NCPHWR) which is funded by Health and Care Research Wales. The funders had no role in the study design, data collection, data analysis, interpretation, writing of the report, or the decision to submit the paper for publication.

Acknowledgements

ADP and the authors would like to acknowledge the data providers who supplied the datasets enabling this research study. The views expressed are entirely those of the authors and should not be assumed to be the same as those of ADP or MQ Mental Health Research Charity. This

For peer review only - http://bmjopen.bmj.com/site/about/guidelines.xhtml

study makes use of anonymised data held in the Secure Anonymised Information Linkage (SAIL) Databank. We would like to acknowledge all the data providers who make anonymised data available for research.

Contributors

LEC: study design, literature search, data curation, data analysis, figures, data interpretation, writing – original draft, writing – review and editing; KH: data analysis, data interpretation, supervision, writing – review and editing; JS: conceptualisation, study design, methodology, data curation, data analysis, data interpretation, supervision, writing – review and editing; TW: methodology, writing – review and editing; JT: validation, writing – review and editing; AJ: methodology, funding acquisition, writing – review and editing; AB: methodology, writing – review and editing; ARD: conceptualisation, study design, data analysis, data interpretation, supervision, funding acquisition, writing – review and editing. LEC and JS verified the underlying data.

Competing interests

Ann John is a member of the Welsh Government COVID-19 Technical Advisory Group and is also co-chair of the Scientific Pandemic Insights Group on Behaviours (SPI-B) which is a subgroup of the Scientific Advisory Group for Emergencies (SAGE) advising the UK government. None of the other authors have any competing interests.

Data availability statement

The data used in this study are available in the SAIL Databank at Swansea University (Swansea, UK) via the Adolescent Mental Health Data Platform, but, as restrictions apply, they are not publicly available. All proposals to use SAIL data are subject to review by an independent Information Governance Review Panel (IGRP). Before any data can be accessed, approval must be given by the IGRP. The IGRP carefully considers each project to ensure proper and appropriate use of SAIL data. When access has been granted, it is gained

For peer review only - http://bmjopen.bmj.com/site/about/guidelines.xhtml

through a privacy-protecting safe haven and remote access system referred to as the SAIL Gateway. SAIL have established an application process to be followed by anyone who would like to access data via SAIL, details of which can be found at https://saildatabank.com/data/apply-to-work-with-the-data/

References

1 Davies A, Song J, Bentley L, *et al.* COVID-19 in Wales: The impact on levels of health care use and mental health of the clinically extremely vulnerable. Cardiff: Public Health Wales. 2021. https://phw.nhs.wales/services-and-teams/knowledge-directorate/research-and-evaluation/publications/covid-19-in-wales-the-impact-on-levels-of-health-care-use-and-mental-health-of-the-clinically-extremely-vulnerable/ (accessed March 2, 2022).

2 NHS Wales Informatics Service. COVID-19 high risk shielded patient list identification methodology. 2020. https://nwis.nhs.wales/coronavirus/coronavirus-content/coronavirus-documents/covid-19-high-risk-shielded-patient-list-identification-methodology-v3-2-17th-august/ (accessed Dec 10, 2020).

3 Department of Health and Social Care. Statement from the UK Chief Medical Officers on schools and childcare reopening. 2020. https://www.gov.uk/government/news/statement-from-the-uk-chief-medical-officers-on-schools-and-childcare-reopening (accessed Dec 12, 2020).

4 Di Gessa G, Price D. The impact of shielding during the COVID-19 pandemic on mental health: evidence from the English Longitudinal Study of Ageing. *Br J Psychiatry* 2022; published online April 4. https://doi.org/10.1192/bjp.2022.44.

5 Mansfield KE, Mathur R, Tazare J, *et al.* Indirect acute effects of the COVID-19 pandemic on physical and mental health in the UK: a population-based study. *Lancet Digit Heal* 2021; **3**: e217–30.

6 Carr MJ, Steeg S, Webb RT, *et al.* Effects of the COVID-19 pandemic on primary care-recorded mental illness and self-harm episodes in the UK: a population-based cohort study. *Lancet Public Heal* 2021; **6**: e124–35.

7 Crawley E, Loades M, Feder G, Logan S, Redwood S, Macleod J. Wider collateral damage to children in the UK because of the social distancing measures designed to reduce the impact of COVID-19 in adults. *BMJ Paediatr Open* 2020; **4**: e000701.

8 Newlove-Delgado, T Williams T, Robertson K, McManus S, *et al.* Mental health of children and young people in England, 2021. Wave 2 follow up to the 2017 survey. 2021. https://files.digital.nhs.uk/97/B09EF8/mhcyp_2021_rep.pdf (accessed Dec 15, 2021).

9 Vizard T, Sadler K, Ford T, *et al.* Mental health of children and young people in England, 2020. Wave 1 follow up to the 2017 survey. 2020. https://files.digital.nhs.uk/AF/AECD6B/mhcyp_2020_rep_v2.pdf (accessed April 5, 2021).

10 Raw JAL, Waite P, Pearcey S, Shum A, Patalay P, Creswell C. Examining changes in parent-reported child and adolescent mental health throughout the UK's first COVID-19 national lockdown. *J Child Psychol Psychiatry* 2021; **62**: 1391–401.

11 Department for Education. State of the Nation 2021: Children and young people's

For peer review only - http://bmjopen.bmj.com/site/about/guidelines.xhtml

wellbeing. Research report. 2022. https://assets.publishing.service.gov.uk/government/uploads/system/uploads/attachment_data/file/1053302/State_of_the_Nation_CYP_Wellbeing_2022.pdf (accessed April 7, 2022).
12 Welsh Government. Shielded households (composition and characteristics during the coronavirus (COVID-19) pandemic: July 2020. 2020. https://gov.wales/shielded-households-composition-and-characteristics-during-coronavirus-covid-19-pandemic-july-2020 (accessed Nov 14, 2020).
13 Hysing M, Elgen I, Gillberg C, Lie SA, Lundervold AJ. Chronic physical illness and mental health in children. Results from a large-scale population study. *J Child Psychol Psychiatry* 2007; **48**: 785–92.
14 Griffiths H, O'Connor K, Phillips B, *et al.* Meeting the psychological needs of children in shielding families. 2020. https://www.bps.org.uk/sites/www.bps.org.uk/files/Policy/Policy - Files/Meeting the psychological needs of children in shielding families.pdf (accessed Nov 17, 2020).
15 Welsh Government. Children living in shielded households during the coronavirus (COVID-19) pandemic: June 2020. 2020. https://gov.wales/children-living-shielded-households-during-coronavirus-covid-19-pandemic-june-2020-html (accessed Nov 15, 2020).
16 Kassa C, Pavlopoulou G. Lonely lockdown. Life for siblings of disabled children in the UK. 2021. https://doi.org/10.14324/000.rp.10125424.
17 Lyons RA, Jones KH, John G, *et al.* The SAIL databank: linking multiple health and social care datasets. *BMC Med Inform Decis Mak* 2009; **9**: 3.
18 Rodgers S, Lyons R, Dsilva R, *et al.* Residential Anonymous Linking Fields (RALFs): a novel information infrastructure to study the interaction between the environment and individuals' health. *J Public Health* 2009; **31**: 582–8.
19 Schnier C, Wilkinson T, Akbari A, *et al.* The Secure Anonymised Information Linkage databank Dementia e-cohort (SAIL-DeC). *Int J Popul Data Sci* 2020; **5**: 1121.
20 Schultze A, Bates C, Cockburn J, *et al.* Identifying care home residents in electronic health records - an OpenSAFELY short data report. *Wellcome Open Res* 2021; **6**: 90.
21 Paranjothy S, Evans A, Bandyopadhyay A, *et al.* Risk of emergency hospital admission in children associated with mental disorders and alcohol misuse in the household: an electronic birth cohort study. *Lancet Public Health* 2018; **3**: e279–88.
22 John A, Marchant AL, McGregor JI, *et al.* Recent trends in the incidence of anxiety and prescription of anxiolytics and hypnotics in children and young people: an e-cohort study. *J Affect Disord* 2015; **183**: 134–41.
23 John A, Marchant AL, Fone DL, *et al.* Recent trends in primary-care antidepressant prescribing to children andyoung people: an e-cohort study. *Psychol Med* 2016; **46**: 3315.
24 Welsh Government. Welsh Index of Multiple Deprivation (WIMD) 2019: Results report. 2019. https://gov.wales/sites/default/files/statistics-and-research/2019-11/welsh-index-multiple-deprivation-2019-results-report-024.pdf (accessed Nov 20, 2020).
25 Benchimol EI, Smeeth L, Guttmann A, *et al.* The REporting of studies Conducted using Observational Routinely-collected health Data (RECORD) statement. *PLoS Med* 2015; **12**: e1001885.
26 Campbell OLK, Bann D, Patalay P. The gender gap in adolescent mental health: A cross-national investigation of 566,829 adolescents across 73 countries. *SSM – Population Health* 2021; 13, 100742.
27 Ma L, Mazidi M, Li K, et al. Prevalence of mental health problems among children

For peer review only - http://bmjopen.bmj.com/site/about/guidelines.xhtml

and adolescents during the COVID-19 pandemic: A systematic review and meta-analysis. *Journal of Affective Disorders* 2021; 78-89.
28 O'Connor RC, Wetherall K, Cleare S, et al. Mental health and well-being during the COVID-19 pandemic: longitudinal analyses of adults in the UK COVID-19 Mental Health & Wellbeing study. *The British Journal of Psychiatry* 2021; 218: 326-333.
29 Torjesen I. Covid-19: Only a third of children in need accessed mental health support in the pandemic. *BMJ* 2022; **376**: o335.
30 Young Minds. Coronavirus: Impact on young people with mental health needs. 2020. https://www.youngminds.org.uk/media/04apxfrt/youngminds-coronavirus-report-summer-2020.pdf (accessed Nov 2, 2020).
31 Cornish RP, John A, Boyd A, Tilling K, Macleod J. Defining adolescent common mental disorders using electronic primary care data: A comparison with outcomes measured using the CIS-R. *BMJ Open* 2016; **6**: e013167.
32 Centre for Mental Health. Covid-19 and the nation's mental health: October 2020. 2020. https://www.centreformentalhealth.org.uk/publications/covid-19-and-nations-mental-health-october-2020 (accessed April 8, 2021).
33 Welsh Government. Pledge to support youth with extra £9.4m investment in children and young people mental health services. 2021. https://gov.wales/pledge-support-youth-extra-ps94m-investment-children-and-young-people-mental-health-services (accessed May 2, 2021).

Figure captions

Figure 1. Flow diagram of the inclusion criteria for the creation of three study cohorts: clinically extremely vulnerable children, children living with a clinically extremely vulnerable person, and children in the general population

CEV, clinically extremely vulnerable; CVSP, COVID-19 Shielded People List; SAIL, Secure Anonymised Information Linkage; GP, general practice; RALF, residential anonymised linking field; NHS, national health service.

Figure 2. Kaplan-Meier survival curves for each cohort, showing the time to first record of anxiety or depression between 23rd March 2020 and 31st January 2021 (2020/21)

Figure 3. Kaplan-Meier survival curves for each cohort, showing the time to first record of anxiety or depression between 23rd March 2019 and 31st January 2020 (2019/20)

For peer review only - http://bmjopen.bmj.com/site/about/guidelines.xhtml

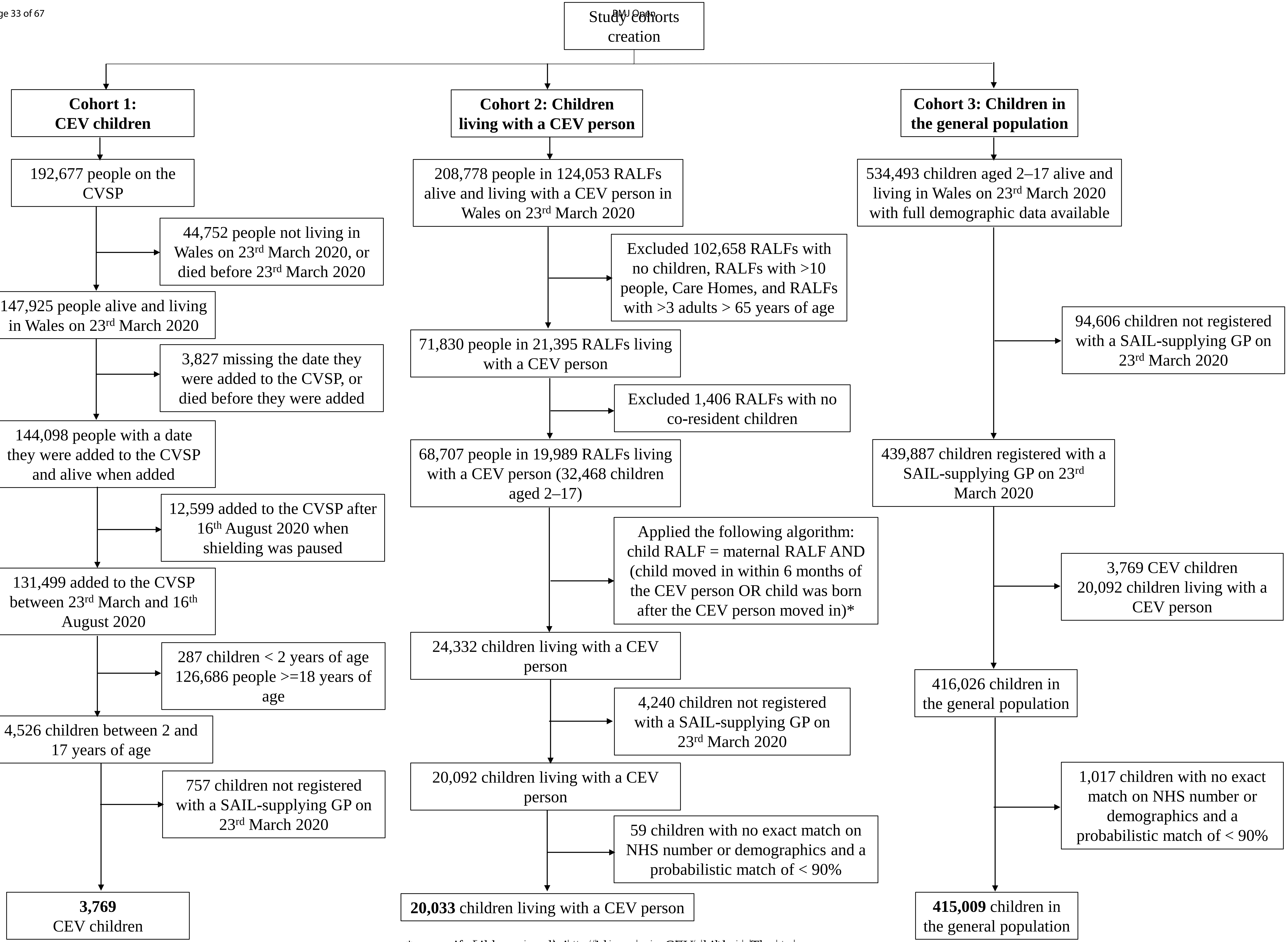

*except if children were living with younger CEV children. These children were included if they shared the same maternal ALF

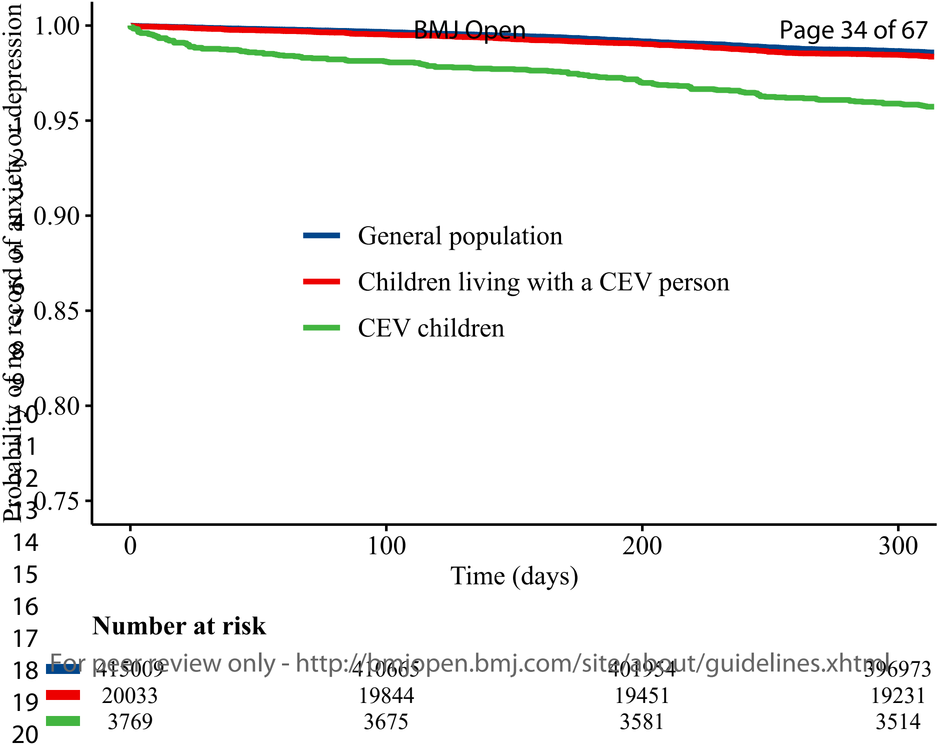
Probability of no record of anxiety or depression
1.00
0.95
0.90
0.85
0.80
0.75
General population
Children living with a CEV person
CEV children
0
100
200
300
Time (days)
Number at risk
20033
19844
19451
19231
3769
3675
3581
3514
396973

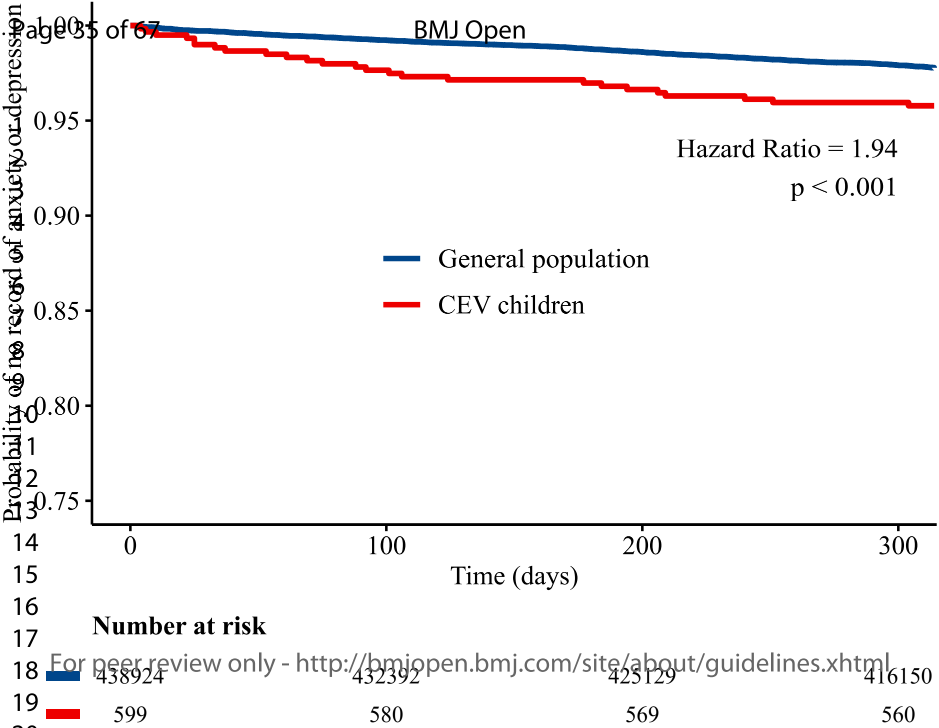
Probability of no record of anxiety or depression
1.00
0.95
0.90
0.85
0.80
0.75
Hazard Ratio = 1.94
p < 0.001
General population
CEV children
0
100
200
300
Time (days)
Number at risk
438924
432392
425129
416150
599
580
569
560

Appendix

SAIL databank additional information

SAIL databank information governance policies and procedures

The SAIL databank (www.saildatabank.com) is an internationally recognised, remotely-accessible, privacy-protecting data safe haven designed to support observational, interventional, and policy-relevant research to improve population health, well-being, and services.[1–6] SAIL contains anonymised, linkable, routinely collected health, administrative, and social care data for the population of Wales, UK, from multiple sources at individual, household, and ecological levels.[1–6] Data are anonymised using a split-file process, which has been described in detail elsewhere.[1–3] Within each dataset, identifiable and non-identifiable data are separated, and identifiable data are sent to a trusted third party (TTP), Digital Health and Care Wales (DHCW; previously known as the NHS Wales Informatics Service). The TTP uniquely matches identities based on name, NHS number, date of birth, and Unique Property Reference Number (UPRN), using the Matching Algorithm for Consistent Results in Anonymised Linkage, which has an accuracy of 99.85%.[1,2] Individuals and residences are then assigned unique identifiers: for individuals this is called an Anonymised Linking Field (ALF) and for residences a Residential Anonymised Linking Field (RALF). The anonymised and non-identifiable data components are then recombined within SAIL and the linking fields are further encrypted and used to anonymously link between datasets. This enables data from multiple sources, including general practice (GP) data, hospital admissions, outpatient data, and demographic details to be linked at the individual and household level, while preserving anonymity.

References

For peer review only - http://bmjopen.bmj.com/site/about/guidelines.xhtml

1. Lyons RA, Jones KH, John G, et al. The SAIL databank: linking multiple health and social care datasets. BMC Med Inform Decis Mak2009;9:3.doi:10.1186/1472-6947-9-3
2. Ford DV, Jones KH, Verplancke JP, et al. The SAIL Databank: building a national architecture for e-health research and evaluation. BMC Health Serv Res2009;9:157.doi:10.1186/1472-6963-9-157
3. Rodgers SE, Lyons RA, Dsilva R, et al. Residential Anonymous Linking Fields (RALFs): a novel information infrastructure to study the interaction between the environment and individuals health. J Public Health2009;31:582–8.doi:10.1093/pubmed/fdp041
4. Rodgers SE, Demmler JC, Dsilva R, Lyons RA. Protecting health data privacy while using residence-based environment and demographic data. *Health Place*. 2012; 18: 209-217.
5. Jones, K. H., Ford, D. V., Thompson, S., & Lyons, R. A. (2019). A profile of the SAIL databank on the UK secure research platform. *International Journal of Population Data Science*, *4*(2). https://doi.org/10.23889/ijpds.v4i2.1134
6. Jones, K. H., Ford, D. V., Jones, C., Dsilva, R., Thompson, S., Brooks, C. J., Heaven, M. L., Thayer, D. S., McNerney, C. L., & Lyons, R. A. (2014). A case study of the secure anonymous information linkage (SAIL) gateway: A privacy-protecting remote access system for health-related research and evaluation. *Journal of Biomedical Informatics*, *50*, 196–204. https://doi.org/10.1016/j.jbi.2014.01.003

For peer review only - http://bmjopen.bmj.com/site/about/guidelines.xhtml

Ethical Approval

The Information Governance Review Panel (IGRP) is an independent panel of representatives from various government, regulatory, and professional organisations, who review all proposals for SAIL data access to ensure that they are appropriate with respect to Information Governance, and in the public interest.[1] Data were analysed within the SAIL secure research environment, and appropriate disclosure control procedures were followed to ensure that no personally identifiable data or small numbers (n<5) were removed from the environment. All data within SAIL are treated in accordance with the Data Protection Act 2018 and SAIL complies with the principles of the General Data Protection Regulation (GDPR).

References

1. Ford DV, Jones KH, Verplancke JP, et al. The SAIL Databank: building a national architecture for e-health research and evaluation. BMC Health Serv Res2009;9:157.doi:10.1186/1472-6963-9-157

For peer review only

ICD-10 codes for cancers of the blood or bone marrow

ICD-10 code	Description
C81	Hodgkin lymphoma
C82	Follicular lymphoma
C83	Non-follicular lymphoma
C84	Mature T/NK-cell lymphomas
C85	Other and unspecified types of non-Hodgkin lymphoma
C86	Other specified types of T/NK-cell lymphoma
C88	Malignant immunoproliferative diseases
C90	Multiple myeloma and malignant plasma cell neoplasms
C91	Lymphoid leukaemia
C92	Myeloid leukaemia
C93	Monocytic leukaemia
C94	Other leukaemias of specified cell type
C95	Leukaemia of unspecified cell type
C96	Other and unspecified malignant neoplasms of lymphoid, haematopoietic and related tissue
D470	Histiocytic and mast cell tumours of uncertain and unknown behaviour
D475	Chronic eosinophilic leukaemia [hypereosinophilic syndrome]
D477	Other specified neoplasms of uncertain or unknown behaviour of lymphoid, haematopoietic and related tissue
D479	Neoplasm of uncertain or unknown behaviour of lymphoid, haematopoietic and related tissue, unspecified
D595	Paroxysmal nocturnal haemoglobinuria [Marchiafava-Micheli]
D71X	Functional disorders of polymorphonuclear neutrophils
D730	Hyposplenism
D760	Langerhans' cell histiocytosis, not elsewhere classified
D761	Haemophagocytic lymphohistiocytosis
D898	Other specified disorders involving the immune mechanism, not elsewhere classified
D899	Disorder involving the immune mechanism, unspecified
L412	Lymphomatoid papulosis
P615	Transient neonatal neutropenia

ICD-10 codes for respiratory illnesses

ICD-10 code	Description
E84	Cystic Fibrosis
J84	Other interstitial pulmonary diseases
J620	Pneumoconiosis due to talc dust
J630	Aluminosis (of lung)
J631	Bauxite fibrosis (of lung)
J633	Graphite fibrosis (of lung)
J634	Siderosis
J635	Stannosis

For peer review only - http://bmjopen.bmj.com/site/about/guidelines.xhtml

J660	Byssinosis
J661	Flax-dresser disease
J662	Cannabinosis
J668	Airway disease due to other specific organic dusts
J670	Farmer lung
J671	Bagassosis
J678	Hypersensitivity pneumonitis due to other organic dusts
J684	Chronic respiratory conditions due to chemicals, gases, fumes and vapours
J688	Other respiratory conditions due to chemicals, gases, fumes and vapours
J698	Pneumonitis due to other solids and liquids
J701	Chronic and other pulmonary manifestations due to radiation
J703	Chronic drug-induced interstitial lung disorders
J840	Alveolar and parietoalveolar conditions
J983	Compensatory emphysema
J991	Respiratory disorders in other diffuse connective tissue disorders
M313	Wegener granulomatosis
P250	Interstitial emphysema originating in the perinatal period
Q334	Congenital bronchiectasis

OPCS-4 codes for immunosuppression therapy

**OPCS-4 code**	**Description**
X353	Active Inflammatory thyroid eye disease patients currently on weekly intravenous steroid infusion treatment (12 weekly injections regime) or immunospressant
X374	Intramuscular Immunotherapy
X385	Subcutaneous Immunotherapy
X891	Monoclonal antibodies Band 1
X892	Monoclonal antibodies Band 2
X893	Patients receiving maintenance treatment with rituximab, obinotuzimab or ofatumumab
X894	Somatostatin analogues Band 1
X895	Allergic emergency drugs Band 1
X961	Patients previously treated for haematological malignancy requiring IV immunoglobulin replacement
X962	Allergen immunotherapy drugs Band 1
X963	Poison management drugs Band 1

For peer review only - http://bmjopen.bmj.com/site/about/guidelines.xhtml

Pre-COVID-19 study cohorts

Figure 3 shows a flow diagram of inclusion criteria for the two 2019 study cohorts: children in the general population, and clinically extremely vulnerable children with blood or bone cancer or respiratory illnesses, or receiving immunosuppression therapy.

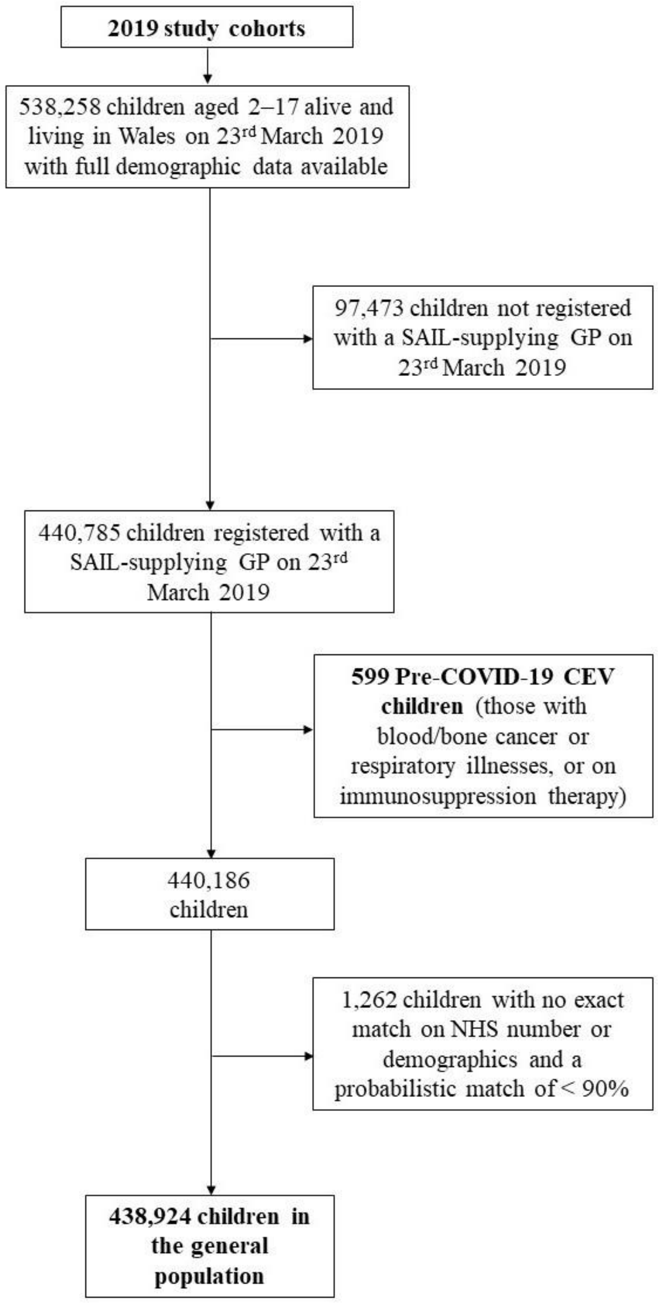

Figure 3. Flow diagram of the inclusion criteria for the creation of the 2019 study cohorts

For peer review only - http://bmjopen.bmj.com/site/about/guidelines.xhtml

Sensitivity Analysis

We undertook a sensitivity analysis to confirm the validity of creating a cohort of pre-COVID-19 CEV children based on just three of the categories included in the COVID-19 Shielded People List (CVSP). We created a cohort of clinically extremely vulnerable (CEV) children who were added to the CVSP in 2020 for the same three reasons only (respiratory illnesses, blood/bone cancer, and immunosuppression therapy) and a cohort of children in the general population (figure 1). Demographic characteristics of the children in each cohort are presented in Table 1. We plotted the Kaplan-Meier survival curve for each cohort and estimated the hazard ratio of having a record for anxiety or depression during the COVID-19 pandemic (March 23rd 2020–January 31st 2021) using Cox regression (figure 2).

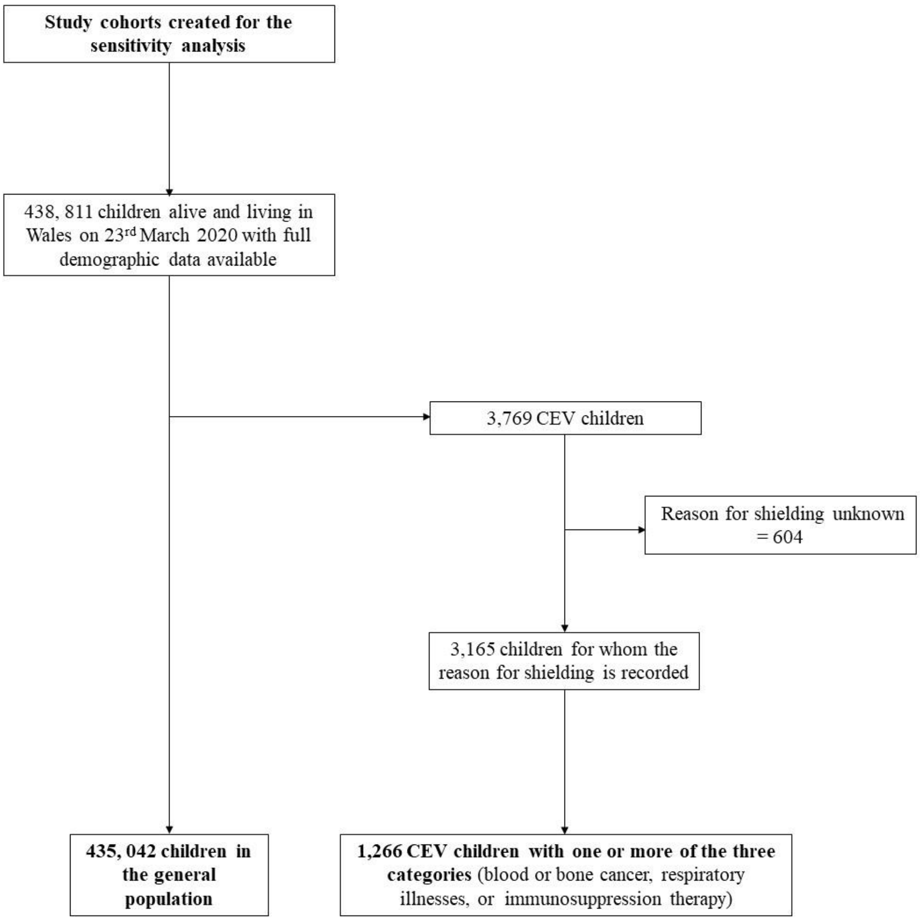

Figure 1. Flow diagram of the inclusion criteria for the creation of the study cohorts for the sensitivity analysis.

		General population 2020	CEV children with one or more of three categories 2020	Chi² P value
N		435,042	1,266	
**Sex (%)**	Male	222,558 (51.2)	712 (56.2)	<0.001
	Female	212,484 (48.8)	554 (43.8)	
**Age group (%)**	2–7	157,691 (36.2)	385 (30.4)	<0.001
	8–12	143,988 (33.1)	396 (31.3)	
	13–17	133,363 (30.7)	485 (38.3)	
**Deprivation quintile (WIMD 2019) (%)**	1 (most deprived)	111,133 (25.5)	329 (26.0)	0.262
	2	92,037 (21.2)	257 (20.3)	
	3	76,717 (17.6)	210 (16.6)	
	4	73,277 (16.8)	203 (16.0)	
	5 (least deprived)	81,878 (18.8)	267 (21.1)	
**Rural/Urban area (%)**	Rural	116,126 (26.7)	336 (26.5)	0.928
	Urban	318,916 (73.3)	930 (73.5)	
**Any history of anxiety or depression**	NO	415,925 (95.6)	1,166 (92.1)	<0.001
	YES	19,117 (4.4)	100 (7.9)	

Table 1. Demographic characteristics of children in the general population and clinically extremely vulnerable children added to the COVID-19 Shielded People List for one or more of three reasons (blood or bone cancer, respiratory illnesses, or immunosuppression therapy)

The results showed a similar pattern to those obtained when analysing the full CEV cohort in that CEV children with one or more of the three categories were at greater risk of having a record for anxiety or depression during the COVID-19 pandemic (March 23rd 2020–January 31st 2021) compared to children in the general population, thereby validating our approach.

For peer review only - http://bmjopen.bmj.com/site/about/guidelines.xhtml

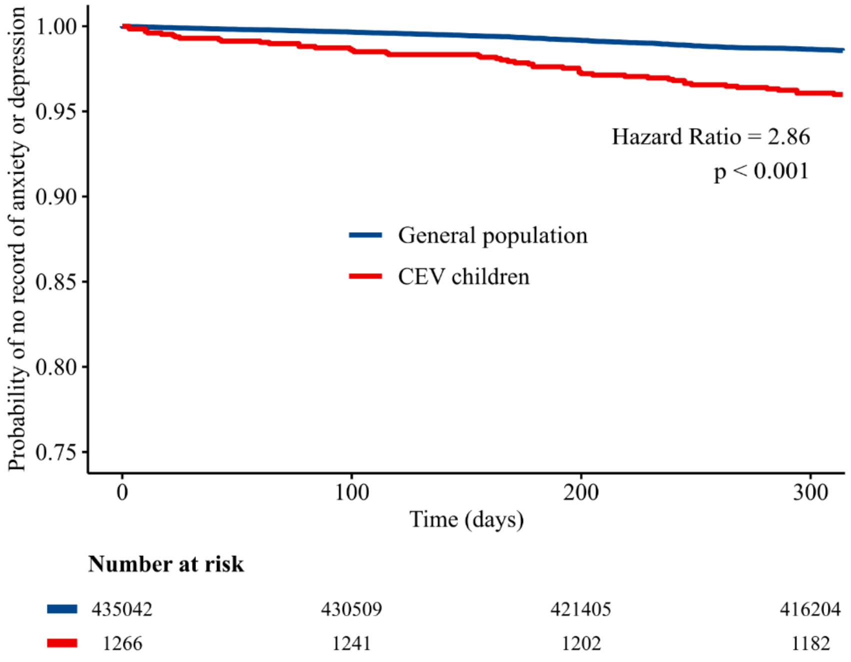

Figure 2. Kaplan-Meier survival curves for each cohort, showing the time to first record of anxiety or depression during the COVID-19 pandemic

CEV, clinically extremely vulnerable

For peer review only - http://bmjopen.bmj.com/site/about/guidelines.xhtml

Read v2 and ICD-10 diagnosis codes used to identify records of anxiety or depression in primary or secondary care

Read v2 codes for depression diagnosis

Read v2 code	Description
Eu32.	[X]Depressive episode
Eu320	[X]Mild depressive episode
Eu321	[X]Moderate depressive episode
Eu322	[X]Severe depressive episode without psychotic symptoms
Eu324	[X]Mild depression
Eu32y	[X]Other depressive episodes
Eu32z	[X]Depressive episode, unspecified
Eu33.	[X]Recurrent depressive disorder
Eu330	[X]Recurrent depressive disorder, current episode mild
Eu331	[X]Recurrent depressive disorder, current episode moderate
Eu332	[X]Recurrent depressive disorder, current episode severe without psychotic symptoms
Eu334	[X]Recurrent depressive disorder, currently in remission
Eu33y	[X]Other recurrent depressive disorders
Eu33z	[X]Recurrent depressive disorder, unspecified
Eu341	[X]Dysthymia
E118.	Seasonal affective disorder
E135.	Agitated depression
E2B..	Depressive disorder NEC
E2B1.	Chronic depression
E291.	Prolonged depressive reaction
E204.	Neurotic depression reactive type
E2B0.	Postviral depression
E112.	Single major depressive episode
E1120	Single major depressive episode, unspecified
E1121	Single major depressive episode, mild
E1122	Single major depressive episode, moderate
E1123	Single major depressive episode, severe, without psychosis
E1125	Single major depressive episode, partial or unspecied remission
E1126	Single major depressive episode, in full remission
E112z	Single major depressive episode NOS
E113.	Recurrent major depressive episode
E1130	Recurrent major depressive episodes, unspecified
E1131	Recurrent major depressive episodes, mild
E1132	Recurrent major depressive episodes, moderate
E1133	Recurrent major depressive episodes, severe, no psychosis
E1135	Recurrent major depressive episodes, partial/unspecified remission
E1136	Recurrent major depressive episodes, in full remission
E1137	Recurrent depression
E113z	Recurrent major depressive episode NOS

For peer review only

Read v2 codes for depression symptoms

Read v2 code	Description
1B17.	Depressed
1B1U.	Symptoms of depression
1BQ..	Loss of capacity for enjoyment
1BT..	Depressed mood
1BU..	Loss of hope for the future
2257	O/E – depressed
1BP..	Loss of interest

Read v2 codes for antidepressant prescriptions

Read v2 code	Description
d71..	Amitriptyline hydrochloride
d72..	Butriptyline - discontinued
d73..	Clomipramine hydrochloride
d74..	Desipramine hydrochloride
d75..	Dosulepin Hydrochloride
d76..	Doxepin
d77..	Imipramine hydrochloride
d78..	Iprindole
d79..	Lofepramine
d7a..	Maprotiline hydrochloride
d7b..	Mianserin hydrochloride
d7c..	Nortriptyline
d7d..	Protriptyline hydrochloride
d7e..	Trazadone hydrochloride
d7f..	Trimipramine
d7g..	Viloxazine hydrochloride
d7h..	Amoxapine
d81..	Phenelzine
d83..	Isocarboxazid
d84..	Tranylcypromine
d85..	Moclobemide
d91..	Compound Antidepressants A-Z
da1..	Flupentixol [Antidepressant]
da2..	Tryptophan
da3..	Fluvoxamine Maleate
da4..	Fluoxetine hydrochloride
da5..	Sertraline hydrochloride
da6..	Paroxetine hydrochloride
da7..	Venlafaxine
da9..	Citalopram
daA..	Reboxetine
daB..	Mirtazapine

For peer review only - http://bmjopen.bmj.com/site/about/guidelines.xhtml

daC..	Escitalopram
daD..	Agomelatine
gde..	Duloxetine
d911.	Limbitrol 5 capsules - discontinued
d912.	Limbitrol 10 capsules - discontinued
d913.	Motipress tablets
x28CP	Discontinued
d914.	Motival tablets discontinued
d916.	Triptafen tablets - only one not discontinued?
d917.	Triftafen-M tablets - discontinued
d8…	Monoamine-oxidase
d82..	Iproniazid

Read v2 codes for anxiety diagnosis

Read v2 code	Description
Eu41.	[X]Other anxiety disorders
Eu410	[X]Panic disorder [episodic paroxysmal anxiety]
Eu411	[X]Generalized anxiety disorder
Eu413	[X]Other mixed anxiety disorders
Eu41y	[X]Other specified anxiety disorders
Eu41z	[X]Anxiety disorder, unspecified
E200.	Anxiety states
E2000	Anxiety state unspecified
E2001	Panic disorder
E2002	Generalised anxiety disorder
E2004	Chronic anxiety
E2005	Recurrent anxiety
E200z	Anxiety state NOS
E202	Phobic disorders
Eu40.	Phobic anxiety disorder
Eu930	[X]Separation anxiety disorder of childhood *
Eu931	[X]Phobic anxiety disorder of childhood*
Eu932	[X]Social anxiety disorder of childhood*
E2D0.	Disturbance of anxiety and fearfulness in childhood and adolescence*
E2D00	Childhood and adolescent overanxiousness disturbance*
E2D0z	Disturbance of anxiety and fearfulness in childhood and adolescence NOS*

Read v2 codes for anxiety symptoms

Read v2 code	Description
1B13.	Anxiousness
2258	O/E - anxious
1B12.	Nerves, nervousness
R2y2.	(D) nervousness

For peer review only - http://bmjopen.bmj.com/site/about/guidelines.xhtml

2259	O/E nervous
225J.	O/E panic attack
1B1V.	C/O panic attack

Read v2 codes for anxiety prescriptions – hypnotics

Read v2 code	Description
d11..	Chloral hydrate
d12..	Clomethiazole edisylate (hypnotic)
d13..	Dichloralphenazone - discontinued
d14..	Flumtrazepam - discontinued
d15..	Flurazepam
d16..	Loprazolam
d17..	Lormetazepam
d18..	Nitrazepam
d1a..	Temazepam (hynotic)
d1b..	Triazolam - discontinued
d1c..	Triclofos sodium
d1d..	Zopiclone
d1f..	Zolpidem
d1g..	Zaleplon
d1h..	Melatonin
d1i..	Dexmedetomidine

Read v2 codes for anxiety prescriptions – hypnotics

Read v2 code	Description
d21..	Diazepam
d22..	Alprazolam
d23..	Bromazepam
d24..	Chlordiazepoxide
d25..	Chlormezanone
d26..	Clobazam
d27..	Clorazepate dipotassium
d28..	Hydroxyzine hcl (anxiolytic)
d29..	Ketazolam - discontinued
d2a..	Lorazepam (anxiolytic)
d2b..	Medazepam - discontinued
d2c..	Meprobamate
d2d..	Oxazepam
d2e..	Prazepam - discontinued
d2f..	Buspirone hydrocholoride
d2g..	Flumazenil

For peer review only

Read v2 codes for mixed anxiety and depression diagnosis

Read v2 code	Description
E2003	Anxiety with depression
Eu412	[X]Mixed anxiety and depressive disorder

ICD-10 diagnosis codes for depression

ICD-10 code	Description
F321	Moderate depressive episode
F322	Severe depressive episode without psychotic symptoms
F328	Other depressive episodes
F329	Depressive episode, unspecified
F33	Recurrent depressive disorder
F330	Recurrent depressive disorder, current episode mild
F331	Recurrent depressive disorder, current episode moderate
F332	Recurrent depressive disorder, current episode severe without psychotic symptoms
F334	Recurrent depressive disorder, currently in remission
F338	Other recurrent depressive disorders
F339	Recurrent depressive disorder, unspecified
F341	Dysthymia

ICD-10 diagnosis codes for anxiety and mixed anxiety and depression

ICD-10 code	Description
F40	Phobic anxiety disorders
F400	Agoraphobia
F401	Social phobias
F402	Specific (isolated) phobias
F408	Other phobic anxiety disorders
F41	Other anxiety disorders
F410	Panic disorder (episodic paroxysmal anxiety)
F411	Generalized anxiety disorder
F413	Other mixed anxiety disorders
F418	Other specified anxiety disorders
F419	Anxiety disorder, unspecified
F930	Separation anxiety disorder of childhood
F931	Phobic anxiety disorder of childhood
F932	Social anxiety disorder of childhood
F412	Mixed anxiety and depressive disorder

For peer review only - http://bmjopen.bmj.com/site/about/guidelines.xhtml

The RECORD statement – checklist of items, extended from the STROBE statement, that should be reported in observational studies using routinely collected health data.

	Item No.	STROBE items	Location in manuscript where items are reported	RECORD items	Location in manuscript where items are reported
**Title and abstract**					
	1	(a) Indicate the study's design with a commonly used term in the title or the abstract (b) Provide in the abstract an informative and balanced summary of what was done and what was found	(a) The study design is indicated as a data linkage study in the title (b) We have provided an informative and balanced summary in the abstract	RECORD 1.1: The type of data used should be specified in the title or abstract. When possible, the name of the databases used should be included.<br><br>RECORD 1.2: If applicable, the geographic region and timeframe within which the study took place should be reported in the title or abstract.<br><br>RECORD 1.3: If linkage between databases was conducted for the study, this should be clearly stated in the title or abstract.	1.1 The type of data used is specified in the abstract. It was not possible to name all of the databases used in the abstract.<br>1.2 We have reported the geographic region within which the study took place in the title and the abstract, and the timeframe in the abstract<br>1.3 It is clearly stated in the title and the abstract that linkage between databases was conducted for the study
**Introduction**					
Background rationale	2	Explain the scientific background and rationale	The scientific background and rationale for the		

For peer review only - http://bmjopen.bmj.com/site/about/guidelines.xhtml

		for the investigation being reported	investigation is explained in the introduction		
Objectives	3	State specific objectives, including any prespecified hypotheses	The objectives are stated in the introduction		
**Methods**					
Study Design	4	Present key elements of study design early in the paper	The study design is reported at the beginning of the methods section, under the subheading "study design and data source"		
Setting	5	Describe the setting, locations, and relevant dates, including periods of recruitment, exposure, follow-up, and data collection	The setting, locations, and relevant dates are described in the "study population and setting" section		
Participants	6	*(a) Cohort study* - Give the eligibility criteria, and the sources and methods of selection of participants. Describe methods of follow-up *Case-control study* - Give the eligibility criteria, and the sources and methods of case ascertainment and control selection. Give the rationale for the choice of cases and controls	(a) Eligibility criteria, and the sources and methods of selection and follow-up of participants are given in the "study population and setting" section<br>(b) N/A this was not a matched study	RECORD 6.1: The methods of study population selection (such as codes or algorithms used to identify subjects) should be listed in detail. If this is not possible, an explanation should be provided.<br><br>RECORD 6.2: Any validation studies of the codes or algorithms used to select the population should be referenced. If validation was conducted for this study and not	6.1 We have described in detail the methods of study population selection in the "study population and setting" section and the "measures" section<br><br>6.2 We have referenced the validation studies of the anxiety and

For peer review only - http://bmjopen.bmj.com/site/about/guidelines.xhtml

		Cross-sectional study - Give the eligibility criteria, and the sources and methods of selection of participants<br><br>*(b) Cohort study* - For matched studies, give matching criteria and number of exposed and unexposed<br>*Case-control study* - For matched studies, give matching criteria and the number of controls per case		published elsewhere, detailed methods and results should be provided.<br><br>RECORD 6.3: If the study involved linkage of databases, consider use of a flow diagram or other graphical display to demonstrate the data linkage process, including the number of individuals with linked data at each stage.	depression codes we have used in our study in the "measures" section<br><br>6.3 We have provided a flow diagram with the number of individuals included in each cohort at each stage of linkage (Figure 1)
Variables	7	Clearly define all outcomes, exposures, predictors, potential confounders, and effect modifiers. Give diagnostic criteria, if applicable.	We have clearly defined all relevant measures in the "measures" section	RECORD 7.1: A complete list of codes and algorithms used to classify exposures, outcomes, confounders, and effect modifiers should be provided. If these cannot be reported, an explanation should be provided.	7.1 We have provided a list of codes used in the study in the appendix
Data sources/ measurement	8	For each variable of interest, give sources of data and details of methods of assessment (measurement). Describe comparability of assessment methods if there is more than one group	We have described all data sources and measures in the "study design and data sources" section and the "measures" section		

For peer review only - http://bmjopen.bmj.com/site/about/guidelines.xhtml

Bias	9	Describe any efforts to address potential sources of bias	We have explained that we used a conservative approach to identify cohort participants in the "study population and setting" section		
Study size	10	Explain how the study size was arrived at	We have included a flowchart (Figure 1) to describe the creation of each of the study cohorts and explained the process of cohort creation, referred to in the "study population and setting" section		
Quantitative variables	11	Explain how quantitative variables were handled in the analyses. If applicable, describe which groupings were chosen, and why	Handling of quantitative variables is described in the "measures" section		
Statistical methods	12	(a) Describe all statistical methods, including those used to control for confounding<br>(b) Describe any methods used to examine subgroups and interactions<br>(c) Explain how missing data were addressed<br>(d) *Cohort study* - If applicable, explain how	(a) Statistical methods are described in the "statistical analysis" section<br>(b) N/A<br>(c) N/A<br>(d) N/A<br>(e) Sensitivity analyses are described in the appendix		

For peer review only - http://bmjopen.bmj.com/site/about/guidelines.xhtml

		loss to follow-up was addressed<br>*Case-control study* - If applicable, explain how matching of cases and controls was addressed<br>*Cross-sectional study* - If applicable, describe analytical methods taking account of sampling strategy<br>(e) Describe any sensitivity analyses			
Data access and cleaning methods		..		RECORD 12.1: Authors should describe the extent to which the investigators had access to the database population used to create the study population.<br><br>RECORD 12.2: Authors should provide information on the data cleaning methods used in the study.	12.1 We described the extent to which the investigators had access to the database population in the "data access and cleaning methods" section<br>12.2 We provided information on the data cleaning methods in the "data access and cleaning methods" section
Linkage		..		RECORD 12.3: State whether the study included person-level, institutional-level, or other data linkage across two or more databases. The methods of	12.3 The methods of linkage and quality evaluation are detailed in the "study design and data

For peer review only - http://bmjopen.bmj.com/site/about/guidelines.xhtml

				linkage and methods of linkage quality evaluation should be provided.	source" section and the "study population and setting" section and the flowchart (Figure 1)
**Results**					
Participants	13	(a) Report the numbers of individuals at each stage of the study (*e.g.*, numbers potentially eligible, examined for eligibility, confirmed eligible, included in the study, completing follow-up, and analysed)<br>(b) Give reasons for non-participation at each stage.<br>(c) Consider use of a flow diagram	(a) Numbers of individuals at each study stage are provided in the flow diagrams (Figure 1)<br>(b) Reasons for exclusion are provided on the flowchart (Figure 1)<br>(c) We used a flowchart to illustrate selection of our study cohorts (Figure 1)	RECORD 13.1: Describe in detail the selection of the persons included in the study (*i.e.*, study population selection) including filtering based on data quality, data availability and linkage. The selection of included persons can be described in the text and/or by means of the study flow diagram.	13.1 We described in detail the selection of the persons included in the study in the "study population and setting" section and Figure 1
Descriptive data	14	(a) Give characteristics of study participants (*e.g.*, demographic, clinical, social) and information on exposures and potential confounders<br>(b) Indicate the number of participants with missing data for each variable of interest	(a) Characteristics of study participants are given in Table 2<br>(b) N/A<br>(c) N/A		

For peer review only - http://bmjopen.bmj.com/site/about/guidelines.xhtml

		(c) *Cohort study* - summarise follow-up time (*e.g.*, average and total amount)			
Outcome data	15	*Cohort study* - Report numbers of outcome events or summary measures over time<br>*Case-control study* - Report numbers in each exposure category, or summary measures of exposure<br>*Cross-sectional study* - Report numbers of outcome events or summary measures	Numbers of outcome events are reported in the results section in Table 4		
Main results	16	(a) Give unadjusted estimates and, if applicable, confounder-adjusted estimates and their precision (e.g., 95% confidence interval). Make clear which confounders were adjusted for and why they were included<br>(b) Report category boundaries when continuous variables were categorized	(a) We report unadjusted and adjusted estimates in the results section, and have explained why we adjusted for each factor<br>(b) N/A<br>(c) N/A		

For peer review only - http://bmjopen.bmj.com/site/about/guidelines.xhtml

		(c) If relevant, consider translating estimates of relative risk into absolute risk for a meaningful time period			
Other analyses	17	Report other analyses done—e.g., analyses of subgroups and interactions, and sensitivity analyses	We report on sensitivity analyses in the appendix		
**Discussion**					
Key results	18	Summarise key results with reference to study objectives	Key results are summarised at the beginning of the discussion		
Limitations	19	Discuss limitations of the study, taking into account sources of potential bias or imprecision. Discuss both direction and magnitude of any potential bias	Limitations are discussed in the 'Strengths and limitations' section including potential underestimation of our cohorts	RECORD 19.1: Discuss the implications of using data that were not created or collected to answer the specific research question(s). Include discussion of misclassification bias, unmeasured confounding, missing data, and changing eligibility over time, as they pertain to the study being reported.	We discussed the limitations of the Shielded Patient List in the 'Strengths and Limitations' section
Interpretation	20	Give a cautious overall interpretation of results considering objectives, limitations, multiplicity of analyses, results from	We have given a cautious and balanced interpretation of the results with reference to the previous literature in the discussion		

For peer review only - http://bmjopen.bmj.com/site/about/guidelines.xhtml

		similar studies, and other relevant evidence			
Generalisability	21	Discuss the generalisability (external validity) of the study results	Discussed in the "strengths and limitations" section		
**Other Information**					
Funding	22	Give the source of funding and the role of the funders for the present study and, if applicable, for the original study on which the present article is based	We have provided the funding source, and the role of the funders in the "Role of the funders" section		
Accessibility of protocol, raw data, and programming code		..		RECORD 22.1: Authors should provide information on how to access any supplemental information such as the study protocol, raw data, or programming code.	We have provided information on how to access data within the SAIL databank

*Reference: Benchimol EI, Smeeth L, Guttmann A, Harron K, Moher D, Petersen I, Sørensen HT, von Elm E, Langan SM, the RECORD Working Committee. The REporting of studies Conducted using Observational Routinely-collected health Data (RECORD) Statement. *PLoS Medicine* 2015; in press.

*Checklist is protected under Creative Commons Attribution (CC BY) license.

For peer review only - http://bmjopen.bmj.com/site/about/guidelines.xhtml

Demographic characteristics of the 2019 study population

		**General population 2019**	**CEV children 2019**	Chi² P value
N		438,924	599	
**Sex (%)**	Male	224,754 (51.2)	347 (57.9)	<0.001
	Female	214,170 (48.8)	252 (42.1)	
**Age group (%)**	2–7	162,730 (37.1)	231 (38.6)	0.412
	8–12	143,844 (32.8)	181 (30.2)	
	13–17	132,350 (30.2)	187 (31.2)	
**Deprivation quintile (WIMD 2019) (%)**	1 (most deprived)	111,381 (25.4)	134 (22.4)	0.003
	2	93,046 (21.2)	137 (22.9)	
	3	77,600 (17.7)	88 (14.7)	
	4	74,288 (16.9)	94 (15.7)	
	5 (least deprived)	82,609 (18.8)	146 (24.4)	
**Rural/Urban area (%)**	Rural	118,418 (27.0)	165 (27.5)	0.790
	Urban	320,506 (73.0)	434 (72.5)	
**Any history of anxiety or depression**	NO	421,659 (96.1)	565 (94.3)	0.037
	YES	17,265 (3.9)	34 (5.7)	

For peer review only

The RECORD statement – checklist of items, extended from the STROBE statement, that should be reported in observational studies using routinely collected health data.

	Item No.	STROBE items	Location in manuscript where items are reported	RECORD items	Location in manuscript where items are reported
**Title and abstract**					
	1	(a) Indicate the study's design with a commonly used term in the title or the abstract (b) Provide in the abstract an informative and balanced summary of what was done and what was found	(a) The study design is indicated as a data linkage study in the title on page 1<br>(b) We have provided an informative and balanced summary in the abstract on page 2	RECORD 1.1: The type of data used should be specified in the title or abstract. When possible, the name of the databases used should be included.<br><br>RECORD 1.2: If applicable, the geographic region and timeframe within which the study took place should be reported in the title or abstract.<br><br>RECORD 1.3: If linkage between databases was conducted for the study, this should be clearly stated in the title or abstract.	1.1 The type of data used is specified in the abstract on page 2. It was not possible to name all of the databases used in the abstract.<br>1.2 We have reported the geographic region within which the study took place in the title on page 1 and the abstract on page 2, and the timeframe in the abstract on page 2<br>1.3 It is clearly stated in the title (page 1) and the abstract (page 2) that linkage between databases was conducted for the study
**Introduction**					
Background rationale	2	Explain the scientific background and rationale	The scientific background and rationale for the investigation is explained		

		for the investigation being reported	in the introduction on pages 4-5		
Objectives	3	State specific objectives, including any prespecified hypotheses	The objectives are stated in the introduction on pages 5-6		
**Methods**					
Study Design	4	Present key elements of study design early in the paper	The study design is reported at the beginning of the methods section, under the subheading "study design and data source" on page 6		
Setting	5	Describe the setting, locations, and relevant dates, including periods of recruitment, exposure, follow-up, and data collection	The setting, locations, and relevant dates are described in the "study population and setting" section on pages 8-9		
Participants	6	*(a) Cohort study* - Give the eligibility criteria, and the sources and methods of selection of participants. Describe methods of follow-up *Case-control study* - Give the eligibility criteria, and the sources and methods of case ascertainment and control selection. Give the rationale for the choice of cases and controls	(a) Eligibility criteria, and the sources and methods of selection and follow-up of participants are given in the "study population and setting" section on pages 8-9 (b) N/A this was not a matched study	RECORD 6.1: The methods of study population selection (such as codes or algorithms used to identify subjects) should be listed in detail. If this is not possible, an explanation should be provided. RECORD 6.2: Any validation studies of the codes or algorithms used to select the population should be referenced. If validation was conducted for this study and	6.1 We have described in detail the methods of study population selection in the "study population and setting" section on pages 8-9, and the "measures" section on page 10 6.2 We have referenced the validation studies of the anxiety and depression codes we have used in our study

For peer review only

		Cross-sectional study - Give the eligibility criteria, and the sources and methods of selection of participants<br><br>*(b) Cohort study* - For matched studies, give matching criteria and number of exposed and unexposed<br>*Case-control study* - For matched studies, give matching criteria and the number of controls per case		not published elsewhere, detailed methods and results should be provided.<br><br>RECORD 6.3: If the study involved linkage of databases, consider use of a flow diagram or other graphical display to demonstrate the data linkage process, including the number of individuals with linked data at each stage.	in the "measures" section on page 10<br><br>6.3 We have provided a flow diagram with the number of individuals included in each cohort at each stage of linkage (Figure 1), referred to on page 8
Variables	7	Clearly define all outcomes, exposures, predictors, potential confounders, and effect modifiers. Give diagnostic criteria, if applicable.	We have clearly defined all relevant measures in the "measures" section on page 10	RECORD 7.1: A complete list of codes and algorithms used to classify exposures, outcomes, confounders, and effect modifiers should be provided. If these cannot be reported, an explanation should be provided.	7.1 We have provided a list of codes used in the study in the appendix (pages 10-14 of the appendix)
Data sources/ measurement	8	For each variable of interest, give sources of data and details of methods of assessment (measurement). Describe comparability of assessment methods if	We have described all data sources and measures in the "study design and data sources" section on pages 8-9 and the "measures" section on page10		

For peer review only

		there is more than one group			
Bias	9	Describe any efforts to address potential sources of bias	We have explained that we used a conservative approach to identify cohort participants in the "study population and setting" section on page 8		
Study size	10	Explain how the study size was arrived at	We have included a flowchart (Figure 1) to describe the creation of each of the study cohorts and explained the process of cohort creation, referred to in the "study population and setting" section on page 8		
Quantitative variables	11	Explain how quantitative variables were handled in the analyses. If applicable, describe which groupings were chosen, and why	Handling of quantitative variables is described in the "measures" section on page 10		
Statistical methods	12	(a) Describe all statistical methods, including those used to control for confounding<br>(b) Describe any methods used to examine subgroups and interactions	(a) Statistical methods are described in the "statistical analysis" section on page 11<br>(b) N/A<br>(c) N/A<br>(d) N/A<br>(e) Sensitivity analyses are described in the		

For peer review only

		(c) Explain how missing data were addressed<br>(d) *Cohort study* - If applicable, explain how loss to follow-up was addressed<br>*Case-control study* - If applicable, explain how matching of cases and controls was addressed<br>*Cross-sectional study* - If applicable, describe analytical methods taking account of sampling strategy<br>(e) Describe any sensitivity analyses	appendix (pages 7-9 of the appendix)		
Data access and cleaning methods		..		RECORD 12.1: Authors should describe the extent to which the investigators had access to the database population used to create the study population.<br><br>RECORD 12.2: Authors should provide information on the data cleaning methods used in the study.	12.1 We described the extent to which the investigators had access to the database population in the "data access and cleaning methods" section on page 7<br>12.2 We provided information on the data cleaning methods in the "data access and cleaning methods" section on page 7

For peer review only

Linkage		..		RECORD 12.3: State whether the study included person-level, institutional-level, or other data linkage across two or more databases. The methods of linkage and methods of linkage quality evaluation should be provided.	12.3 The methods of linkage and quality evaluation are detailed in the "study design and data source" section on pages 6-7, the "study population and setting" section on pages 8-9, and the flowchart (Figure 1), referred to on page 8
**Results**					
Participants	13	(a) Report the numbers of individuals at each stage of the study (*e.g.*, numbers potentially eligible, examined for eligibility, confirmed eligible, included in the study, completing follow-up, and analysed)<br>(b) Give reasons for non-participation at each stage.<br>(c) Consider use of a flow diagram	(a) Numbers of individuals at each study stage are provided in the flow diagrams (Figure 1, referred to on page 8)<br>(b) Reasons for exclusion are provided on the flowchart (Figure 1, referred to on page 8)<br>(c) We used a flowchart to illustrate selection of our study cohorts (Figure 1, referred to on page 8)	RECORD 13.1: Describe in detail the selection of the persons included in the study (*i.e.,* study population selection) including filtering based on data quality, data availability and linkage. The selection of included persons can be described in the text and/or by means of the study flow diagram.	13.1 We described in detail the selection of the persons included in the study in the "study population and setting" section on pages 8-9 and Figure 1 referred to on page 8
Descriptive data	14	(a) Give characteristics of study participants (*e.g.*, demographic, clinical, social) and information	(a) Characteristics of study participants are given in Table 2, page 13<br>(b) N/A<br>(c) N/A		

For peer review only

		on exposures and potential confounders<br>(b) Indicate the number of participants with missing data for each variable of interest<br>(c) *Cohort study* - summarise follow-up time (*e.g.*, average and total amount)			
Outcome data	15	*Cohort study* - Report numbers of outcome events or summary measures over time<br>*Case-control study* - Report numbers in each exposure category, or summary measures of exposure<br>*Cross-sectional study* - Report numbers of outcome events or summary measures	Numbers of outcome events are reported in the results section in Table 4, pages 14-15		
Main results	16	(a) Give unadjusted estimates and, if applicable, confounder-adjusted estimates and their precision (e.g., 95% confidence interval). Make clear which confounders were	(a) We report unadjusted and adjusted estimates in the results section on pages 15-18, and have explained why we adjusted for each factor<br>(b) N/A<br>(c) N/A		

For peer review only

		adjusted for and why they were included<br>(b) Report category boundaries when continuous variables were categorized<br>(c) If relevant, consider translating estimates of relative risk into absolute risk for a meaningful time period			
Other analyses	17	Report other analyses done—e.g., analyses of subgroups and interactions, and sensitivity analyses	We report on sensitivity analyses in the appendix (pages 7-9 of the appendix)		
**Discussion**					
Key results	18	Summarise key results with reference to study objectives	Key results are summarised at the beginning of the discussion on page 21		
Limitations	19	Discuss limitations of the study, taking into account sources of potential bias or imprecision. Discuss both direction and magnitude of any potential bias	Limitations are discussed in the 'Strengths and limitations' section on page 23 including potential underestimation of our cohorts	RECORD 19.1: Discuss the implications of using data that were not created or collected to answer the specific research question(s). Include discussion of misclassification bias, unmeasured confounding, missing data, and changing eligibility over time, as they pertain to the study being reported.	We discussed the limitations of the Shielded Patient List in the 'Strengths and Limitations' section on page 23

For peer review only

Interpretation	20	Give a cautious overall interpretation of results considering objectives, limitations, multiplicity of analyses, results from similar studies, and other relevant evidence	We have given a cautious and balanced interpretation of the results with reference to the previous literature in the discussion on pages 21-22		
Generalisability	21	Discuss the generalisability (external validity) of the study results	Discussed in the "strengths and limitations" section on page 23		
**Other Information**					
Funding	22	Give the source of funding and the role of the funders for the present study and, if applicable, for the original study on which the present article is based	We have provided the funding source on page 25, and the role of the funders in the "Role of the funders" section on pages 7-8		
Accessibility of protocol, raw data, and programming code		..		RECORD 22.1: Authors should provide information on how to access any supplemental information such as the study protocol, raw data, or programming code.	We have provided information on how to access data within the SAIL databank on pages 26-27

*Reference: Benchimol EI, Smeeth L, Guttmann A, Harron K, Moher D, Petersen I, Sørensen HT, von Elm E, Langan SM, the RECORD Working Committee. The REporting of studies Conducted using Observational Routinely-collected health Data (RECORD) Statement. *PLoS Medicine* 2015; in press.

*Checklist is protected under Creative Commons Attribution (CC BY) license.
